# Chemistry beyond the scale of exact diagonalization on a quantum-centric supercomputer

**DOI:** 10.1126/sciadv.adu9991

**Published:** 2025-06-18

**Authors:** Javier Robledo-Moreno, Mario Motta, Holger Haas, Ali Javadi-Abhari, Petar Jurcevic, William Kirby, Simon Martiel, Kunal Sharma, Sandeep Sharma, Tomonori Shirakawa, Iskandar Sitdikov, Rong-Yang Sun, Kevin J. Sung, Maika Takita, Minh C. Tran, Seiji Yunoki, Antonio Mezzacapo

**Affiliations:** ^1^ IBM Quantum, IBM T. J. Watson Research Center, Yorktown Heights, NY 10598, USA.; ^2^ IBM Quantum, IBM Research Cambridge, Cambridge, MA 02142, USA.; ^3^ IBM Quantum, IBM France Lab, Orsay, France.; ^4^ Department of Chemistry, University of Colorado, Boulder, CO 80302, USA.; ^5^ Computational Materials Science Research Team, RIKEN Center for Computational Science (R-CCS), Kobe, Hyogo 650-0047, Japan.; ^6^ Quantum Computational Science Research Team, RIKEN Center for Quantum Computing (RQC), Wako, Saitama 351-0198, Japan.; ^7^ RIKEN Interdisciplinary Theoretical and Mathematical Sciences Program (iTHEMS), Wako, Saitama 351-0198, Japan.; ^8^ RIKEN Center for Emergent Matter Science (CEMS), Wako, Saitama 351-0198, Japan.

## Abstract

A universal quantum computer can simulate diverse quantum systems, with electronic structure for chemistry offering challenging problems for practical use cases around the hundred-qubit mark. Although current quantum processors have reached this size, deep circuits and a large number of measurements lead to prohibitive runtimes for quantum computers in isolation. Here, we demonstrate the use of classical distributed computing to offload all but an intrinsically quantum component of a workflow for electronic structure simulations. Using a Heron superconducting processor and the supercomputer Fugaku, we simulate the ground-state dissociation of N_2_ and the ground state properties of [2Fe-2S] and [4Fe-4S] clusters, with circuits up to 77 qubits and 10,570 gates. The proposed algorithm processes quantum samples to produce upper bounds for the ground-state energy and sparse approximations to the ground-state wave functions. Our results suggest that, for current error rates, a quantum-centric supercomputing architecture can tackle challenging chemistry problems beyond sizes amenable to exact diagonalization.

## Teaser

Quantum-centric supercomputing enables electronic structure problems not amenable to exact diagonalization before fault tolerance.

## INTRODUCTION

The most common task in theoretical quantum chemistry is the computation of ground-state energies by solving the Schrödinger equation H∣Ψ〉=E∣Ψ〉 in the Born-Oppenheimer approximation. Exact numerical solutions in a finite basis set have a cost growing combinatorially in the number of electrons and orbitals. This limits exact diagonalization in the full configuration interaction (FCI) to system sizes close to 22 electrons in 22 orbitals (22e,22o) (*1*) and (26e,23o) (*2*). For system sizes beyond the reach of FCI, one must rely on approximate methods, e.g., diagrammatic techniques, wave function ansatzes, and Monte Carlo integration (*3*, *4*).

Progress in quantum computing has triggered a flurry of theoretical proposals for computational chemistry over the past decade [e.g., (*5*–*7*)]. At the same time, attempts have been made at implementations on prefault-tolerant quantum processors (*8*–*14*), but these have so far been limited to small systems for two main reasons. First, despite numerous efforts to improve on the measurement problem [e.g., (*15*–*17*)], runtime for energy expectation value estimation on interesting systems remains out of any reasonable timescale. Second, the depths of chemically motivated quantum circuits for computations of chemistry are very high. For unitary coupled cluster (*18*) and a single step of time evolution, these quantities scale as $M^{4}$ (*19*) on a system with M spin-orbitals. Although this scaling can be improved with various techniques (*20*), on prefault-tolerant devices, the signal emerging from circuits of such size is weakened by the accumulation of gate errors and qubit decoherence.

Here, we show that a quantum-centric supercomputing architecture and workflow—which we call sample-based quantum diagonalization (SQD)—allow us to tackle realistic electronic structure problems on system sizes beyond the reach of exact diagonalization on prefault-tolerant quantum processors. We conduct quantum experiments to study the ground-state properties of the N_2_ molecule and the [2Fe-2S] and [4Fe-4S] clusters using 58, 45, and 77 qubits, respectively, and a maximum number of 3.5 K two-qubit gates.

The manuscript is structured as follows. In the Results section, we provide a brief description of the problem statement, the concerted quantum-classical workflow, and the configuration recovery technique, as well as the quantum circuits run in the experiments. This section ends with the presentation of the experiment results on the ground-state properties of the N_2_ molecule in a correlation-consistent basis set and the active spaces of the [2Fe-2S] and [4Fe-4S] clusters. The Discussion section summarizes our findings and examines some conditions for the advantage with SQD or variations thereof. The Materials and Methods section provides detailed explanations on the subspace projection and diagonalization and approximate total spin symmetry restoration, the configuration recovery technique, and experimental details including the construction of the quantum circuits and the mapping into quantum processors.

## RESULTS

**Fig. 1.**
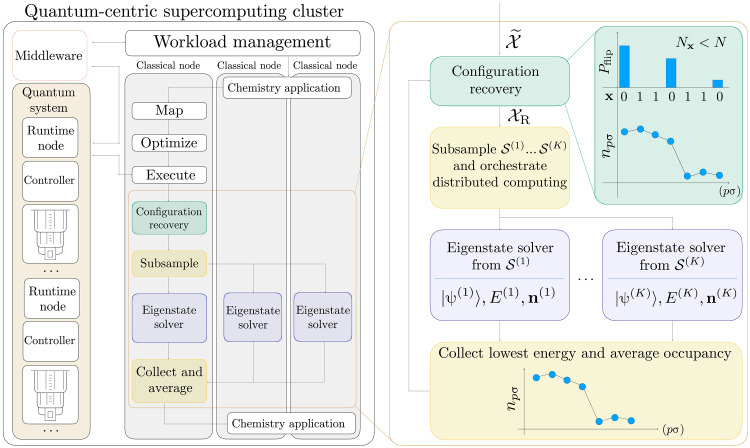
Quantum-centric supercomputing architecture and SQD workflow diagram. (**Left**) We illustrate a simplified architecture used to execute our workflow. The architecture has a cluster with a quantum system alongside classical runtime nodes within an isolated environment. A workload management system controls hybrid quantum-classical jobs through middleware. Our workflow is distributed on a set of classical nodes. It includes standard quantum chemistry application routines such as computing electronic integrals, mapping to qubits, and preparing circuits to be executed. (**Right**) Details of the classical postprocessing step. The input is a set of noisy samples $\tilde{X}$ from the quantum execution that are processed with our configuration recovery step, using information from a vector n of reference orbital occupancies. The green inset shows an example where a configuration with $N_{x}<N$ is corrected. The set of recovered configurations $X_{R}$ is subsampled and distributed for projection and diagonalization on parallel classical nodes. A new average reference occupancy vector **n** is computed from the results, and the configuration recovery loop is repeated self-consistently until convergence.

We set up the discussion of our results by considering the quantum-centric supercomputing architecture (*21*) schematized in Fig. 1. The architecture enables scaling of computational capacity by leveraging quantum processors for their natural task: executing a limited number of large quantum circuits. We follow the workflow in Fig. 1 to summarize our methods.

Our main goal is to find the ground state of chemistry Hamiltonians

$$\begin{array}{c} \hat{H}=\sum_{\begin{array}{c} \mathrm{pr}\\ \sigma \end{array}}h_{\mathrm{pr}}\;{\hat{a}}_{p\sigma }^{†}{\hat{a}}_{r\sigma }+\sum_{\begin{array}{c} \text{prqs}\\ \sigma \tau \end{array}}\frac{\left(\mathrm{pr}\;|\;\mathrm{qs}\right)}{2}\;{\hat{a}}_{p\sigma }^{†}{\hat{a}}_{q\tau }^{†}{\hat{a}}_{s\tau }{\hat{a}}_{r\sigma } \end{array}$$ (1)

expanded over a discrete basis set. Here, we have defined the fermionic creation/annihilation operator ${\hat{a}}_{p\sigma }^{†}/{\hat{a}}_{p\sigma }$ associated to the p-th basis set element and the spin σ, whereas $h_{\mathrm{pr}}$ and (pr∣qs) are the one- and two-body electronic integrals, obtained from standard chemistry software (*22*). Throughout this manuscript, we use molecular orbitals as basis set elements. We map the degrees of freedom of Eq. 1 to qubits with a Jordan-Wigner (JW) transformation (*23*). We then construct a quantum circuit to be executed on quantum hardware, preparing a state ∣Ψ〉 on M qubits, which represents a molecular wave function on M molecular spin-orbitals. In the JW mapping, the single-qubit basis states ∣0〉/∣1〉 represent empty/occupied spin-orbitals. These mapping and optimization steps are performed on classical nodes (see Fig. 1). We execute the circuit on a quantum computer and measure ∣Ψ〉 in the computational basis. Repeating this produces a set of measurement outcomes

$$\tilde{X}=\left\{\;x\;|\;x∼{\tilde{P}}_{\Psi }\left(x\right)\;\right\}$$ (2)

in the form of bitstrings $x\in {\left\{0,1\right\}}^{M}$ distributed according to some ${\tilde{P}}_{\Psi }$; the bitstrings represent electronic configurations, also referred to as Slater determinants (SDs).

### Configuration recovery

On a prefault-tolerant quantum computer, the action of noise alters the distribution from its ideal form $P_{\Psi }={|\;\langle x\;|\;\Psi \rangle \;|}^{2}$ to some other ${\tilde{P}}_{\Psi }$, which generates the noisy set of configurations $\tilde{X}$, accessible to us via quantum measurement. Noise in the quantum system broadens the distribution $P_{\Psi }$ over configurations that do not contribute to low-energy states, so-called deadwood (*24*). As a result, only a fraction of $\tilde{X}$ contains a meaningful quantum signal. To improve this scenario, we introduce a self-consistent configuration recovery technique, which allows a probabilistic partial recovery of noiseless configuration samples from $\tilde{X}$.

The configuration recovery scheme is inspired by the structure of chemistry problems. The Hamiltonian in Eq. 1 conserves the number of particles separately for each spin species. The recovery routine targets configurations x that have the wrong particle number $N_{x}\neq N$ due to the accumulation of errors in the execution of the quantum circuit.

Repeated rounds of recovery can be carried out self-consistently. The first step of each recovery round is to iterate through the set $\tilde{X}$ and find configurations x with $N_{x}\neq N$ particles. If $N_{x}>N$ (or $N_{x}<N$), $|\;N_{x}-N\;|$ bits are sampled to be flipped from the set of occupied (or empty) spin-orbitals, according to a distribution proportional to a monotonically increasing function (see Materials and Methods section for further information) of $|\;x_{p\sigma }-n_{p\sigma }\;|$, the distance from the current value of the bit to the average occupancy of the spin-orbital pσ, obtained from the previous recovery round. This generates a new set of recovered configurations $X_{R}$.

Following the next step of Fig. 1, we build K batches of d configurations $S^{\left(1\right)}\ldots ,S^{\left(K\right)}$ using samples from the set $X_{R}$, according to a distribution proportional to the empirical frequencies of each x in $X_{R}$. We project and diagonalize the Hamiltonian over each $S^{\left(k\right)}:k=1,\ldots ,K$, as proposed recently in the quantum selected configuration interaction (SCI) method (*25*, *26*), which draws inspiration from the classical SCI framework (*27*–*33*).

Each batch of sampled configurations spans a subspace $S^{\left(k\right)}$ in which the many-body Hamiltonian is projected

$$\begin{array}{c} {\hat{H}}_{S^{\left(k\right)}}={\hat{P}}_{S^{\left(k\right)}}{\hat{H}\hat{P}}_{S^{\left(k\right)}},\text{with}\;{\hat{P}}_{S^{\left(k\right)}}=\sum_{x\in S^{\left(k\right)}}|\;x\;\rangle \langle \;x\;|\; \end{array}$$ (3)

The ground states and energies of ${\hat{H}}_{S^{\left(k\right)}}$, which we label $|\;\psi^{\left(k\right)}\;\rangle$ and $E^{\left(k\right)}$, are then computed using the iterative Davidson method on multiple classical nodes. The computational cost—both quantum and classical—to produce $|\;\psi^{\left(k\right)}\;\rangle$ is polynomial in d, the dimension of the subspace.

The ground states are then used to obtain new occupancies

$$n_{p\sigma }=\frac{1}{K}\sum_{1\leq k\leq K}\langle \;\psi^{\left(k\right)}\;|\;{\hat{n}}_{p\sigma }\;|\;\psi^{\left(k\right)}\;\rangle$$ (4)

for each spin-orbital tuple (pσ), averaged on the K batches. These occupancies are sent back to the configuration recovery step, and this entire self-consistent iteration is repeated until convergence, realizing an SQD of the target Hamiltonian. The initial guess for n used for the first round of recovery comes from running SQD using the raw quantum samples in the correct particle sector. The configuration recovery routine can be seen effectively as a problem-informed clustering of a noisy signal around the occupations n. In general, the convergence of the configuration recovery procedure depends on the error rates and the physical properties of the system under consideration. With current error rates, and for the systems in this study, we have observed its convergence within three iteration steps in all systems. We always chose a maximum number of five recovery iterations. We foresee that the lowering of error rates in future quantum hardware will result in faster convergence. Additional details are provided in the Materials and Methods section.

**Fig. 2.**
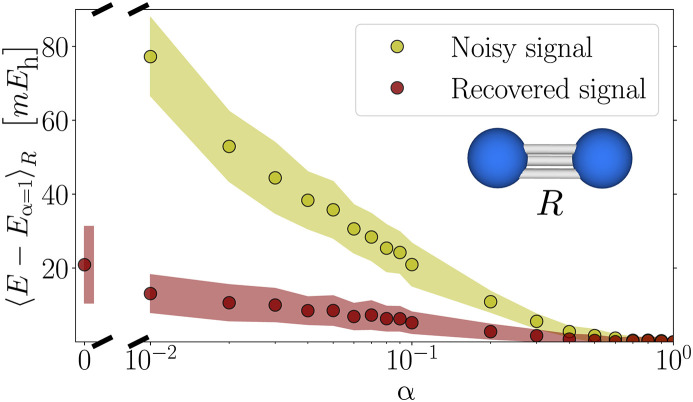
Self-consistent configuration recovery. Energy error (mean and SD) in the dissociation of N_2_ (6-31g), averaged over 24 bond lengths equally spaced between R=0.7Å and R=3.0Å, as a function of quantum signal to noise ratio, parameterized by α. The point at α=0 corresponds to sampling from the uniform distribution (no signal). The energies are obtained via projection and diagonalization of $d=10^{6}$ raw noisy samples against a subspace of the same size obtained by the configuration recovery routine.

To test the noise robustness, we perform numerical simulations that confirm the improvements of applying the configuration recovery routine to the dissociation of N_2_ (6-31G basis set). In this test, we sample from the exact ground state $P_{\Psi_{G}}\left(x\right)={|\;\langle x\;|\;\psi_{G}\rangle \;|}^{2}$ and we set a subspace dimension of $d=10^{6}$. We use a global depolarizing noise channel to model the effect of noise, ${\tilde{P}}_{\Psi_{G}}\left(x\right)=\alpha P_{\Psi_{G}}\left(x\right)+\left(1-\alpha \right)\frac{1}{2^{M}}$, with α∈[0,1] the parameter that controls the amount of noiseless quantum signal. Figure 2 shows the error in the ground-state energy relative to the noiseless case (α=1), as a function of the amount of signal α, for the estimator both with and without configuration recovery. On the N_2_ model, errors below 10*mE*_h_ can be obtained from ~20% signal using the raw noisy samples. However, by using configuration recovery, we can tolerate an ~2% signal to reach the same error. This numerical experiment hints that the use of configuration recovery will be crucial for large-scale experiments.

### Quantum circuits

**Fig. 3.**
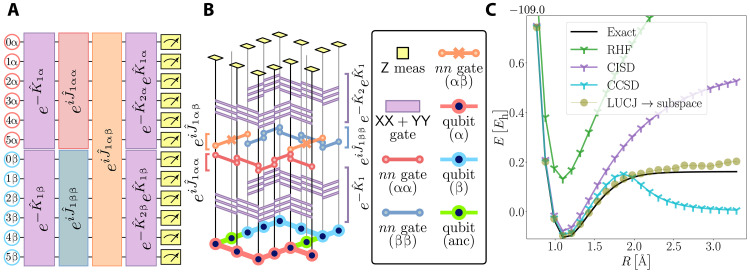
Quantum circuits for chemistry: LUCJ. (**A**) Schematic representation of the truncated LUCJ circuit used to generate the set of samples. The circuit is composed of orbital rotation unitaries $\text{exp}\left(-K_{\mu \sigma }\right)$, same-spin cluster operators $\text{exp}\left[i\left(J_{\mu \alpha \alpha }+J_{\mu \beta \beta }\right)\right]$, and opposite-spin cluster operators $\text{exp}\left({\mathrm{iJ}}_{\mu \alpha \beta }\right)$, with α/β denoting spin-up/down. (**B**) Single unit of the heavy-hex lattice with a compilation of the LUCJ circuit into single- and two-qubit gates, with the *nn* gate defined as $U_{\mathrm{nn}}\left(\varphi \right)=\text{exp}\left[i\varphi \left(1-Z_{0}\right)\left(1-Z_{1}\right)/4\right]$. (**C**) Comparison of the potential energy surface of N_2_ (with a 6-31G basis) obtained from a noiseless subspace simulation of the LUCJ circuit in (A) with L=1, and optimized parameters, against restricted CCSD. RHF, CISD, and exact energies are shown for reference.

Before presenting our experimental results, we discuss the circuits ∣Ψ〉 used to produce the candidate ground states. We use a truncated version of the local unitary cluster Jastrow (LUCJ) ansatz (*34*), shown in Fig. 3A

$$|\Psi \rangle =\prod_{\mu =1}^{L}e^{{\hat{K}}_{\mu }}e^{i{\hat{J}}_{\mu }}e^{-{\hat{K}}_{\mu }}\;|\;x_{\text{RHF}}\rangle$$ (5)

Here, ${\hat{K}}_{\mu }=\sum_{\mathrm{pr},\sigma }K_{\mathrm{pr}}^{\mu }\;{\hat{a}}_{p\sigma }^{†}{\hat{a}}_{r\sigma }$ are generic one-body operators, ${\hat{J}}_{\mu }=\sum_{\mathrm{pr},\sigma \tau }J_{p\sigma ,r\tau }^{\mu }\;{\hat{n}}_{p\sigma }{\hat{n}}_{r\tau }$ are density-density operators restricted to spin-orbitals that are mapped onto adjacent qubits (*34*), and $x_{\text{RHF}}$ is the bitstring representing the restricted Hartree-Fock (RHF) state in the JW mapping. Through this local approximation, the LUCJ ansatz allows for moderate circuit depths. Its accuracy derives from the connection with unitary coupled cluster theory and adiabatic state preparation (*34*–*36*). The moderate depths of LUCJ are due to the use of exponentials of one-body operators, implementable in linear depth and a quadratic number of two-qubit gates, and density-density operators, implementable in constant depth and a linear number of ZZ rotations (due to the locality approximation) (*34*). The LUCJ circuit, compiled into one- and two-qubit gates, is shown in Fig. 3B.

In Fig. 3C, we show a numerical experiment comparing the potential energy curve of N_2_ (6-31G basis) obtained by restricted coupled cluster with singles and doubles (CCSD) to one obtained from the LUCJ ansatz, numerically optimized using the subspace energy as the objective function (see Supplementary Materials for further information). The dimension of the diagonalization subspace for different bond lengths ranging from d=209764 to d=1340964, with a median of d=563250. Because of the presence of strong static correlation, CCSD fails in the description of the dissociation curve, whereas the optimized LUCJ ansatz produces a qualitatively correct dissociation curve. We simulated the LUCJ ansatz using the ffsim library (*37*).

Throughout our experiments, we use the truncated LUCJ circuit $|\;\Psi \;\rangle =e^{-{\hat{K}}_{2}}e^{{\hat{K}}_{1}}e^{i{\hat{J}}_{1}}e^{-{\hat{K}}_{1}}\;|\;x_{\text{RHF}}\;\rangle$, which is the result of considering the L=2 circuit and removing the last orbital rotation and Jastrow operations. We parameterize the LUCJ circuits converting the CCSD wave function in Jastrow form and imposing a locality approximation to the resulting $J^{\mu }$ tensors, i.e., zeroing out the components $J_{p\sigma ,r\tau }^{\mu }$ not corresponding to adjacent qubits (*34*). For systems where the $J_{p\sigma ,r\tau }^{\mu }$ parameters obtained from CCSD have small amplitude, the $e^{-{\hat{K}}_{1}}$ and $e^{{\hat{K}}_{1}}$ terms in the ansatz approximately cancel: In such a situation, without $e^{{\hat{K}}_{2}}$, the resulting wave function is overconcentrated around the Hartree-Fock configuration (see the Materials and Methods section for additional information). Although we initialize the LUCJ circuit with CCSD parameters, the nature of these two methods is very different. In particular, CCSD theory is nonvariational while SQD is. This adds an additional difficulty in the understanding of the relative performance of CCSD and SQD with LUCJ initialized from CCSD parameters. We have performed optimization-free experiments, exploiting the connection between LUCJ and classical coupled cluster theory, yet closing a quantum-classical optimization could further improve the quality of the solutions.

### Implementation on a quantum-centric supercomputing platform 

In the following, we present the experimental results obtained using the methods discussed so far, on Heron quantum processors and the Fugaku supercomputer. The largest experiment is run on a subset of 77 qubits of a 133-qubit Heron quantum processor. The median fidelities for this subset are 99.77% for two-qubits gates, 99.97% for single-qubit, and readout fidelity of 98.37%, with median coherence times $T_{1}=180\;\mu s$ and $T_{2}=150\;\mu s$. In SQD calculations, it is particularly important to have as many measurement outcomes with correct particle number as possible. However, qubits may be initialized imperfectly, i.e., not in the ∣000…000〉 state, resulting in more measurement outcomes with incorrect particle number. To mitigate this source of error, we use a reset-mitigation scheme by adding an additional measurement instruction before the circuit execution and postselecting outcomes based on this first measurement returning the initial state ∣000…000〉. This postselection results in ~1/3 retention rate of all the executions, i.e., qubits collapse initialized in the desired state upon the additional measurement with probability ~ 1/3.

The classical projection and diagonalizations are obtained with the Davidson method implemented in the library PySCF (*22*) on a single node, or DICE (*30*, *33*) for distributed computing on multiple nodes. Convergence to the most accurate solution can be obtained in two ways: increasing the accuracy per diagonalization with the subspace size d, and increasing the number of batches K, which will reduce statistical errors in the analysis. For our largest experiment on the [4Fe-4S] cluster, we use up to d=100M, distributing a single projection and diagonalization to 64 nodes of Fugaku, and K=100 batches, for a total of 6400 nodes. We analyze runtime performance as a function of d and K versus the number of nodes used in the Supplementary Materials. At 64 nodes per diagonalization on the largest experiments, classical runtimes are about 1.5 hours. The largest heat-bath configuration interaction (HCI) calculation that we performed on 16 nodes at d=2.3M took about 16 min.

**Fig. 4.**
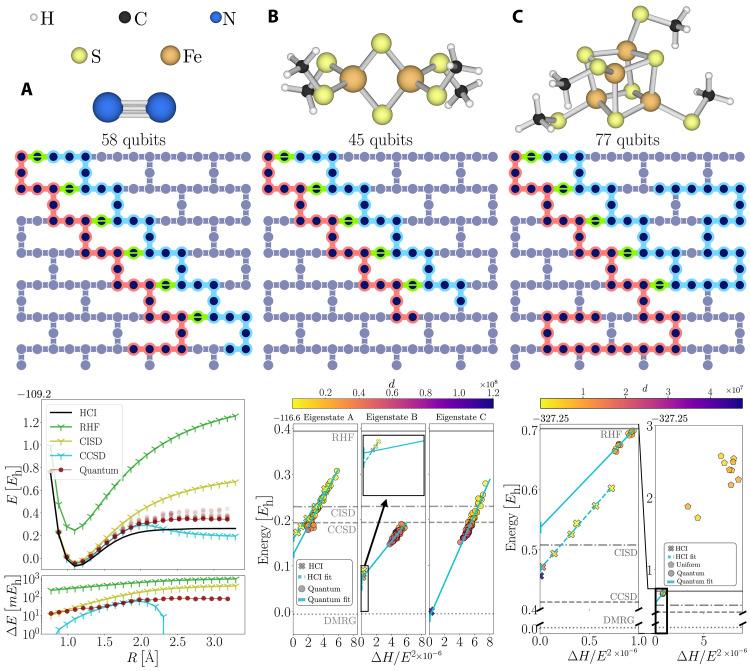
Experiments: Chemistry on large basis sets. (**A**) 58 qubits are used to model the N_2_ dissociation (cc-pVDZ basis set). (**B**) 45 qubits are used for the [2Fe-2S] cluster (TZP-DKH basis set) and (**C**) 77 qubits for the [4Fe-4S] cluster (TZP-DKH basis set). The top panels show a three-dimensional representation of the geometry of each molecule. The middle panels show the qubits selected on a Heron quantum processor layout, following the same color convention as (B) in Fig. 3. The bottom panel in (A) shows the potential energy surface comparison, as well as the energy difference ΔE between the HCI energy and the energies obtained from different methods. The brown scatterplot shows the value of $E^{\left(k\right)}$ for all batches of configurations, and the connected dots show ${\text{min}}_{k}\left(E^{\left(k\right)}\right)$. The bottom panel in (B) shows the energy-variance analysis for three different eigenstates that both HCI and our method find upon increasing the value of *d*, as labeled by the color bar. For each approximate eigenstate $|\;\psi^{\left(k\right)}\;\rangle$, the horizontal axis ΔH=〈ψ(k)∣H^2∣ψ(k)〉−〈ψ(k)∣H^∣ψ(k)〉2. The bottom panel in (C) shows a comparison of the energy-variance analysis applied to quantum measurement outcomes and bitstrings (with the correct particle number) sampled from the uniform distribution. The DMRG energy in (B) and (C) is from (*60*).

We perform two classes of experiments: the breaking of the triple bond of N_2_ (cc-pVDZ basis), in the top panel in Fig. 4A, and the ground states of [2Fe-2S] and [4Fe-4S] clusters (active spaces of the TZP-DKH basis), shown in the top panels in Fig. 4 (B and C). We study the ground-state properties of these molecular systems in the $S_{z}=0$ and $S^{2}=0$ subspace, where $S_{z}$ is the total $\hat{z}$ component of the spin and $S^{2}=S_{x}^{2}+S_{y}^{2}+S_{z}^{2}$. In this work, we used SDs to define the subspaces, which in general are not eigenfunctions of $S^{2}$ unlike configuration state functions (CSFs). In the closed-shell systems studied here, a source of spin contamination is the fact that sampled determinants are not closed under the spin inversion operation. Therefore, we achieved an approximate restoration of the $S^{2}$ symmetry by extending the set of sampled determinants to ensure closure under spin inversion, as detailed in the Materials and Methods section.

#### Triple bond breaking in N_2_

The breaking of the N_2_ bond is a well-known test of the accuracy of electronic structure methods in the presence of static electronic correlation (*38*, *39*). Restricted CCSD theory, a dominant paradigm for the accurate description of weakly correlated systems in quantum chemistry, fails in the description of N_2_ dissociation due to static correlation effects: As correlations become stronger, RHF becomes unstable toward a symmetry-broken unrestricted Hartree-Fock (UHF) state. CCSD built from RHF predicts an artificial barrier to binding and overcorrelates at dissociation, whereas CCSD built from a UHF reference dissociates correctly at the cost of spin contamination, a manifestation of Löwdin’s symmetry dilemma. We use a correlation-consistent cc-pVDZ basis set, to place emphasis on a theory’s ability to treat both dynamic and static correlation in an accurate and balanced manner. We map the N_2_ molecule onto a Heron processor as shown in the middle panel of Fig. 4A. We project and diagonalize a Hamiltonian using $d=16\times 10^{6}$ configurations. We consider K=10 batches of configurations, and each point in the dissociation curve has $|\;\overset{˜}{X}\;|=100\times 10^{3}$ measurement outcomes and 10 iterations of recovery. The combined quantum runtime for all points in the dissociation curve is ~45 min. The experimental data are reported in the bottom panel of Fig. 4A, showing the potential energy surface of N_2_ compared to classical approximate methods. The data from our experiments are consistent with other classical methods except for CCSD, which fails in the description of the dissociation as seen for the smaller basis set considered in Fig. 4B. Among the classical SCI methods, HCI (*30*) obtains the best results for N_2_ and will be our reference classical method in all the other experiments. The difference between our method and HCI energies is everywhere within tens of *mE*_h_. We further analyze the accuracy of our experiments as a function of d and the effect of orbital optimizations in the accuracy of the predictions in the Supplementary Materials. This first test demonstrates that we are capable of addressing multireference ground states and builds confidence for the next set of experiments, which will focus on assessing the ability of the quantum-classical architecture to do precision many-body physics.

#### [2Fe-2S] cluster: Precision many-body physics

Iron-sulfur (FeS) clusters are molecular ensembles of sulfide-linked 1- to 8-iron centers in variable oxidation states. They are important cofactors in biological processes ranging from nitrogen fixation to photosynthesis and respiration (*40*). Their electronic structure, with multiple low-lying states of differing electronic and magnetic character, is responsible for their rich chemistry. At the same time, they pose considerable challenges for experimental studies and numerical tools. For our experiments, we consider the synthetic [Fe_2_S_2_(SCH_3_)_4_]^−2^ cluster (*41*), abbreviated [2Fe-2S] and used in numerical studies to mimic the oxidized dimers prominently found in ferredoxins (*42*).

The qubit mapping of the LUCJ circuit on the Heron processor for [2Fe-2S] is shown in the middle panel of Fig. 4B. We consider K=10 batches of configurations and $|\;\overset{˜}{X}\;|=2.4576\times 10^{6}$ measurement outcomes. The quantum runtime for this system is ~45 min. For the [2Fe-2S] cluster, we perform an energy-variance analysis of the low-energy spectrum of the molecule. The energy-variance analysis is a tool routinely used in classical computational electronic structure to capture the convergence of the approximate eigenstate energy for different levels of accuracy of a computational method (*43*). Here, we use energy-variance analysis to assess the convergence as a function of d, which is directly related to quantum and classical accuracy, runtimes, and costs. If one can statistically sample from a good approximation of an eigenstate, points at finite number of samples will be distributed linearly in the energy-variance plane (*43*). This gives us a tool to detect eigenstates for both quantum and classical methods.

The bottom panel in Fig. 4B shows an energy-variance comparison of HCI and SQD run for different subspace dimensions. As the subspace dimension is increased, three eigenstates can be identified, which we label A, B and, C. The extrapolation of the quantum data to the zero-variance limit is in good agreement with the HCI extrapolations for the same eigenstates. The extrapolated value of the energy for eigenstate C is in good agreement with the density matrix renormalization group (DMRG) calculation from (*44*).

#### [4Fe-4S] cluster: A stress test for methodology and quantum processors

The circuits considered for the N_2_ experiments and [2Fe-2S] reached sizes of ~1 to 1.5k two-qubit gates. We now test the quality of the signal in noisy circuits that test the limits of Heron processors, using up to 6400 nodes of Fugaku for the classical processing. We consider the synthetic [Fe_4_S_4_(SCH_3_)_4_]^−2^ cluster (*41*), abbreviated [4Fe-4S], a representative of nature’s cubanes, whose ground-state deduction from experimental measurements was an early success of inorganic spectroscopy (*45*). The LUCJ circuit used for this molecular species contains ~3.5k two-qubit gates. The qubit mapping on the Heron processor for [4Fe-4S] is shown in the middle panel of Fig. 4C. As in the previous experiment, we consider K=10 batches of configurations and $|\;\overset{˜}{X}\;|\;=2.4576\times 10^{6}$ measurement outcomes. The quantum runtime for this system is ~45 min. For $d>250\times 10^{3}$, configuration recovery is warm started with the n obtained from the method at $d=250\times 10^{3}$, and only two iterations are then performed.

This last set of experiments sheds light on the quality of the quantum signal that is passed to the configuration recovery at these large circuit sizes. The bottom right panel in Fig. 4C shows a comparison of the energy-variance analysis from measurement outcomes obtained from the Heron processor and configurations sampled from the uniform distribution. We see that, even if the quantum solutions produced are worse than other classical methods, the energy and variance obtained from quantum data are notably lower than those obtained from uniformly distributed configurations (i.e., pure noise), on subspaces of the same size. This confirms that there is a valuable signal at circuit sizes of ~3.5k two-qubit gates.

## DISCUSSION

### Significance for quantum computing

Current quantum computers in isolation can perform calculations on systems sufficiently large that exact brute-force classical solutions are not available (*46*, *47*). However, these studies have targeted spin systems, leading to circuits that match the connectivity and the measurement and coherence budgets of the quantum devices. In this work, we present electronic structure calculations on active spaces beyond the scale where FCIs are available. Key to achieve this result is the use of classical and quantum computers in concert to implement the SQD method.

SQD makes economical use of quantum computing resources by drawing samples from a single quantum circuit. Although, in principle, other estimators, such as the standard ones used in variational quantum eigensolvers, have bounded variance for any wave function, the dire scaling of a number of measurements to estimate energies makes them impractical for the molecules targeted in this work (*48*). It is also more robust against quantum noise because reconstructing the exact ground-state probability distribution on a quantum computer is not required to get accurate energy approximations, as long as one is sampling relevant configurations (i.e., in the ground-state support).

We have used an LUCJ class of quantum circuits that can reproduce a low-rank decomposition and sparsification of the quantum unitary CCSD (qUCCSD), which allowed us to keep circuit depths manageable (*34*). Lower error rates on quantum operations will allow us to access deeper quantum circuits with higher connectivity, giving access to more general probability distributions. We have performed optimization-free experiments exploiting the connection between LUCJ and classical coupled cluster theory, for the purpose of assessing accuracy and scalability, yet closing a quantum-classical optimization in future work will further improve the quality of our samples.

### Generalization

The SQD method can be applied to simulation tasks other than quantum chemistry, if the target ground-state wave function can be accurately approximated by a sparse vector. Developing quantum circuits with polynomially sized support in the computational basis will be an important element of the generalization of SQD as these circuits are the sources of samples processed by classical computers.

To counter wave function broadening on current quantum hardware, we have used a self-consistent configuration recovery method, exploiting a problem-inspired clustering that leverages the average occupation numbers of the molecular orbitals. We foresee generalizations of our configuration recovery technique to problems other than quantum chemistry that are not informed by the physics of the problem. Conversely, for specific applications, one could use even more information about the problem.

### Implications in the search for quantum advantage

Computations can be ranked against three parameters: runtime, energy or cost, and accuracy. Although the first two are often easy to measure, ranking by accuracy is in general not straightforward. For methods that produce upper bounds to the ground-state energy, including SQD, the expectation value of the Hamiltonian defines an unconditional accuracy metric: A lower energy is ranked as a higher quality, all other conditions (e.g., total spin) being equal. Comparing SQD energies, for example, allowed us to benchmark our results against SCI and uniform configuration sampling on a 77-qubit experiment, without access to exact solutions. In addition, the approximate wave functions produced here can be stored in classical memory, which permits a classical prover to certify them, and allows their manipulation by further classical processing.

Using the expectation value of the Hamiltonian as an accuracy metric, one can easily and naturally rank SQD results along with those of variational classical methods, giving the search for this specific form of quantum advantage a quantitative meaning. Because every variational classical method has a specific domain of applicability (*49*), identifying areas where SQD may offer an accuracy advantage is a delicate problem. For example, variational quantum Monte Carlo methods (*50*, *51*), of paramount importance in many-body physics, are sensitive to the structures of the probability distributions they are modeling, not just to their supports. Similarly, methods based on tensor networks (*52*, *53*) are successfully used to tackle strongly correlated problems in chemistry, granted the ability to converge their energies with bond dimensions, but convergence can be challenging in some cases (*54*, *55*) because of its computational cost and its sensitivity to the nature and ordering of the basis-set orbitals. Developing an in-depth understanding of SQD through extensive numeric and methodological investigations is necessary to establish or rule out advantage in strictly variational ground-state simulations.

SQD shares with SCI the assumption that the ground state may be approximated by a sparse linear combination of determinants, i.e., with a number of determinants much smaller than the Hilbert space dimension. Note that this assumption does not necessarily underline variational Monte Carlo or tensor networks. Therefore, it is natural to look for conditions to improve over SCI. One such condition is the existence of a quantum circuit that produces subspaces of better quality, and more efficiently, than classical heuristic selection methods. In the search for ground states, the quality of a subspace can be determined by a lower variational energy. We conduct numerical experiments that suggest that there exist LUCJ circuits whose samples produce subspaces of better quality than the HCI classical selection heuristic. The LUCJ circuit under consideration shares the same depth and connectivity as the circuits used in the experiments. In this study, we consider a particular flavor of SCI, HCI (*29*), and the [2Fe-2S] cluster (20 orbitals → 40+ qubits).

**Fig. 5.**
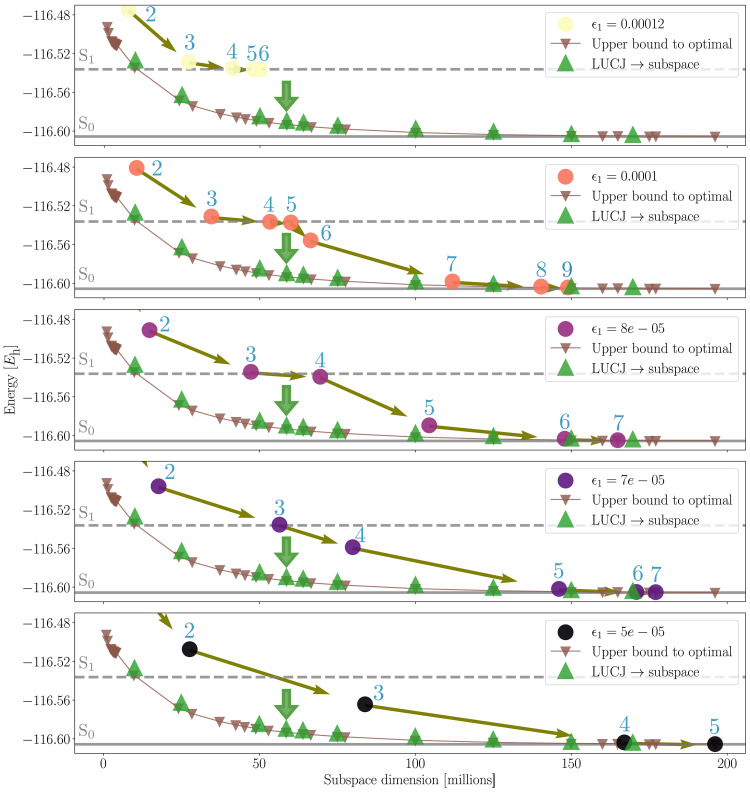
Comparison of the quality of subspaces generated by HCI and an optimized LUCJ circuit in for the description of the ground state of [2Fe-2S]. Different panels show the energy as a function of subspace dimension of the corresponding diagonalization. The dots connected by arrows show the trajectory of HCI in the energy-subspace dimension plane. Each dot is labeled by the iteration it corresponds to. Different panels correspond to HCI calculations carried out with different values of the selection cutoff $\epsilon_{1}$, as indicated in the legend. Brown triangles show energies and subspace dimensions obtained after truncating the most accurate HCI wave function by repeatedly removing the electronic configurations corresponding to the lowest wave function amplitudes. The green triangles show the energies for different subspace dimensions obtained from the optimized LUCJ circuit. The green arrows show the subspace dimension that the circuit was optimized for (see main text). The horizontal solid and dashed lines indicate the DMRG estimates for the S_0_ and S_1_ eigenstates.

We first perform HCI calculations with different values of the selection cutoff $\epsilon_{1}$ [see (*56*) for details on the definition of $\epsilon_{1}$], as shown in Fig. 5. We observe that the larger and more restrictive values of $\epsilon_{1}$ do not allow HCI to reach the DMRG energy reference. Instead, it converges to the first excited state (S_1_) energy. Decreasing the value of $\epsilon_{1}$ allows the subspace dimension to become larger and improve the energy beyond that first excited state, at the cost of a notable increase in subspace dimension, which results in a higher computational cost. From the largest HCI wave function (last point for $\epsilon_{1}=5\times 10^{-5}$), we repeatedly remove the electronic configurations whose wave function amplitude is below a given threshold and compute the energy in the resulting subspace, providing a collection of energy-subspace dimension pairs that we label as “upper bound to optimal” in Fig. 5. The removal of tens of millions of configurations (resulting in substantially smaller subspace dimensions) does not substantially deteriorate the quality of the ground-state approximation. These results indicate that HCI does not perform an optimal search of the relevant electronic configurations. Consequently, smaller subspaces exist that yield comparable energy values. In this particular molecule, HCI needs to explore and perform diagonalizations in subspaces larger than the optimal search.

We optimize an LUCJ circuit to produce samples in the identified optimal subspaces with high probability. The optimal parameters are found in a two-step optimization workflow. First, we optimize the Kullback-Leibler divergence between samples drawn from the LUCJ circuit and the amplitudes of 58 M-dimensional ground-state wave function estimation (green arrow in Fig. 5). Then, the circuit parameters are further fine-tuned to minimize the SQD energy using a differential-evolution strategy.

With the samples produced by the optimized LUCJ circuit, we proceed to evaluate the energy of resulting subspaces of varying dimensionality, including the 58 M-dimensional subspace that the circuit was optimized for. We observe good agreement between the LUCJ subspaces and those that are an upper bound to the optimal ones. It is worth noting that, despite being optimized for a 58 M-dimensional subspace, the resulting circuit produces subspaces of outstanding quality of dimensionalities both smaller and larger.

The active space considered for the [2Fe-2S] cluster is small enough for HCI to be able to find the relevant bitstrings by exploring and performing diagonalizations in subspaces notably larger than the optimal ones. This shows that, in some problem instances, the diagonalization itself is not the runtime bottleneck. Instead, the runtime bottleneck is a suboptimal proposal of electronic configurations. Larger and more strongly correlated systems will pose challenges for the classical heuristics. It is for these systems that one can look for a quantum advantage in sampling.

Characterizing the domain of applicability of different classical and quantum heuristics facilitates combining them in quantum-centric supercomputing environments, shifting the search for quantum advantage toward practical problems.

## MATERIALS AND METHODS

### Conventions and notation

#### Hamiltonian

Our starting point is the Born-Oppenheimer Hamiltonian, written in second quantization using a basis of $N_{\text{MO}}$ orthonormal orbitals ${\left\{\varphi_{p}\right\}}_{p=1}^{N_{\text{MO}}}$, as shown in Eq. 1. We define the number operator ${\hat{n}}_{p\sigma }={\hat{a}}_{p\sigma }^{†}{\hat{a}}_{p\sigma }$, which describes the number of electrons with spin σ on orbital p. For nonrelativistic all-electron calculations, the quantities

$$\begin{array}{c} \begin{array}{ll} E_{0} & =\sum_{a<b}^{N_{\mathrm{nuc}}}\frac{Z_{a}Z_{b}}{∥R_{a}-R_{b}∥}\\ h_{\mathrm{pr}} & =\int dr\;\varphi_{p}^{*}\left(r\right)\;\left[-\frac{1}{2}\;\frac{\partial^{2}}{\partial r^{2}}-\sum_{a=1}^{N_{\mathrm{nuc}}}\frac{Z_{a}}{∥r-R_{a}∥}\right]\;\varphi_{r}\left(r\right)\;\\ \left(\mathrm{pr}|\mathrm{qs}\right) & =\int dr_{1}\int dr_{2}\;\frac{\varphi_{p}^{*}\left(r_{1}\right)\varphi_{r}\left(r_{1}\right)\;\varphi_{q}^{*}\left(r_{2}\right)\varphi_{s}\left(r_{2}\right)}{∥r_{1}-r_{2}∥}\; \end{array} \end{array}$$ (6)

describe the internuclear electrostatic interaction energy and the one-electron and two-electron parts of the Hamiltonian, respectively [atomic units are used throughout, i.e., lengths and energies are measured in Bohr and Hartree units $a_{B}=\hbar^{2}/\left(m_{e}e^{2}\right)$ and $E_{h}=e^{2}/a_{B}$, respectively, where −e and $m_{e}$ are the electron charge and mass]. The symbols $N_{\mathrm{nuc}}$, $R_{a}$, and $Z_{a}$ denote the total number of nuclei and their positions and atomic numbers, respectively.

For relativistic and/or active-space calculations, the indices p,r,q,s label active-space orbitals, and the quantities $E_{0}$, $h_{\mathrm{pr}}$, and (pr∣qs) are modified to account for relativistic effects and/or the potential generated by the inactive-electron density.

In this work, we use orbitals from a restricted closed-shell Hartree-Fock calculation (also called molecular orbitals and denoted MOs) as the basis functions $\varphi_{p}$. Furthermore, we denote $N_{\sigma }$ the number of spin-σ electrons in the exact ground state. Furthermore $N_{↑}+N_{↓}=N$, the total number of electrons in the exact ground state.

#### Molecular species and active spaces

The molecules simulated in this work are listed in Table 1. For N_2_, we studied all non-core electrons and orbitals on quantum hardware. We computed the potential energy curve using (i) RHF at 6-31G, and cc-pVDZ level (*57*–*59*) using PySCF (*22*, *60*) and enforcing $D_{\infty h}$ symmetry and, after projecting the nonrelativistic Born-Oppenheimer Hamiltonian in the space spanned by all non-core RHF orbitals with standard functionalities, with (ii) restricted and symmetry-preserving Moller-Plesset second-order perturbation theory (MP2), CCSD, complete active-space configuration interaction (CASCI), for 6-31G and SCI, in its heat-bath flavor (HCI), for cc-pVDZ.

**Table 1.** Molecules studied in this work. For each molecule, we list the number of electrons and orbitals (N and $N_{\text{MO}}$, respectively) studied on quantum hardware, along with the underlying basis set and the dimension $D={\left(\begin{array}{c} N_{\text{MO}}\\ N/2 \end{array}\right)}^{2}$ of the Hilbert space of $N_{↑}=N_{↓}=N/2$ electrons in $N_{\text{MO}}$ spatial orbitals (not considering molecular point-group symmetries).

Molecule	Basis	$\left(N,N_{\text{MO}}\right)$	D
N_2_	6-31G	(10e,16o)	$1.91\times 10^{7}$
N_2_	cc-pVDZ	(10e,26o)	$4.32\times 10^{9}$
[2Fe-2S]	TZP-DKH	(30e,20o)	$2.40\times 10^{8}$
[4Fe-4S]	TZP-DKH	(54e,36o)	$8.86\times 10^{15}$

For [2Fe-2S] and [4Fe-4S], we used active spaces (*41*, *61*), spanned by Fe[3d] and S[3p] orbitals, derived from a localized density functional theory calculation with BP86 functional (*44*, *62*), TZP-DKH basis (*63*), and sf-X2C (spin-free exact two-component) Hamiltonian (*64*, *65*) to include scalar relativistic effects. We computed approximations to the ground-state energy with restricted RHF, MP2, CCSD, and HCI. The RHF, MP2, CCSD, configuration interaction singles and doubles (CISD), CASCI, and HCI calculations were carried out with the PySCF library (*22*).

#### Electron configurations and qubit mapping

In this work, we represent many-electron states using qubit states with the JW transformation, which maps an electronic configuration, i.e., an SD of the form

$$|\;x\;\rangle =\prod_{p\sigma }{\left({\hat{a}}_{p\sigma }^{†}\right)}^{x_{p\sigma }}|\;\emptyset \;\rangle$$ (7)

where ∣∅ ∅〉 is the vacuum state (i.e., the state with zero electrons) and $x_{p\sigma }\in \left\{0,1\right\}$, onto an element of the computational basis

$$|\;x\;\rangle =\underset{p\sigma }{⊗}|\;x_{p\sigma }\;\rangle$$ (8)

labeled by a bitstring $x=\left(x_{N_{\text{MO}}-1↓}\ldots x_{0↓}x_{N_{\text{MO}}-1↑}\ldots x_{0↑}\right)$. The first half of the bitstring is denoted by $x_{↓}=\left(x_{N_{\text{MO}}-1↓}\ldots x_{0↓}\right)$ and the second half of the bitstring is denoted by $x_{↑}=\left(x_{N_{\text{MO}}-1↑}\ldots x_{0↑}\right)$. The JW mapping uses $M=2N_{\text{MO}}$ qubits and allows computing the number of spin-σ electrons for a given configuration x as $N_{\mathrm{x\sigma}}=\sum_{p}x_{p\sigma }$. The total number of electrons, $N_{x}=\sum_{\sigma }N_{x\sigma }$, is the Hamming weight of x. At the single-configuration level, any x in the right particle sector must satisfy: $N_{x\sigma }=N_{\sigma }$. An important example is the RHF bitstring

$$\begin{array}{c} |\;x_{\text{RHF}}\;\rangle =\;\left|\;\underset{N_{\text{MO}}-N_{↓}}{\underbrace{0\ldots 0}}\underset{N_{↓}}{\underbrace{1\ldots 1}}\underset{N_{\text{MO}}-N_{↑}}{\underbrace{0\ldots 0}}\underset{N_{↑}}{\underbrace{1\ldots 1}}\right.\rangle \; \end{array}$$ (9)

which, by construction, has $N_{x_{\text{RHF}}\sigma }=N_{\sigma }$.

The Fock space is the vector space containing all possible electronic configurations for $N_{\text{MO}}$ orbitals with all possible filling factors for each spin sector. The terms determinant and electronic configuration are used interchangeably in the manuscript.

### Sample-based quantum diagonalization

This section provides a detailed description of the SQD procedure. We describe the subspace projection and diagonalization and the self-consistent configuration recovery scheme. We also motivate the energy-variance analysis presented in Fig. 4.

In what follows, we use X and $\tilde{X}$ to denote a set of configurations sampled from probability distributions $P_{\Psi }\left(x\right)=|\;\langle \;x\;{|\;\Psi \rangle \;|}^{2}$ and ${\tilde{P}}_{\Psi }\left(x\right)=\langle \;x\;|\;\overset{˜}{\rho }\;|\;x\;\rangle$, where $\tilde{\rho }$ is a density operator corresponding to a noisy counterpart of ∣Ψ〉〈Ψ∣. We denote $X_{N}\subset \tilde{X}$ the subset of configurations with the right particle number. The set of configurations recovered by the configuration recovery procedure is denoted by $X_{\to N}$ and $X_{R}=X_{N}\cup X_{\to N}$ the set of configurations output by self-consistent configuration recovery, and $S^{\left(k\right)}\subset X_{R}$ with k=1…K a set of approximately d configurations sampled from $X_{R}$. The wave function $|\psi^{\left(k\right)}\rangle$ in obtained by projection and diagonalization of $\hat{H}$ in the subspace spanned by the configurations in $S^{\left(k\right)}$.

#### Eigenstate solver

Given a set of d electronic configurations, the many-electron Hamiltonian is projected and diagonalized in the subspace spanned by the single-particle states defined by the electronic configurations, as proposed recently in (*25*). We begin by considering a set of configurations $X=\left\{\;x_{\left(i\right)}\;\right\}$, all with the right-particle number for each spin sector. Subindices between parenthesis label configurations in a set and not configuration components. We use the generic label X for a set of configurations with the right particle number. In practice, the configuration recovery procedure will execute the eigenstate solver on the sets $X_{N}$ and $X_{R}$.

##### Conservation of spin

In this study, we perform an approximate restoration of the total spin symmetry labeled by the ${\hat{S}}^{2}$ quantum number. In particular, we are interested on wave functions $|\;\psi^{\left(k\right)}\;\rangle$ that are as close as possible to singlet states, i.e., eigenfunctions of total spin with eigenvalue 0, ${\hat{S}}^{2}|\;\psi^{\left(k\right)}\;\rangle =0$. Conservation of symmetries is notoriously important because molecular eigenstates are joint eigenfunctions of $\hat{H}$ and its symmetries, including ${\hat{S}}_{z}$, $\hat{N}$, molecular point-group symmetries (if any), and ${\hat{S}}^{2}$. Conservation of ${\hat{S}}_{z}$ and $\hat{N}$ (and molecular point-group symmetries that are isomorphic to ${\mathbb{Z}}_{2}^{\times n}$) can be achieved in a relatively easy way because the eigenfunctions of these operators are SDs ∣x〉, e.g.

N^∣x〉=(Nαx+Nβx)∣x〉,S^z∣x〉=(Nαx−Nβx)∣x〉 (10)

Therefore, in this work, we ensure that the configurations used to span the subspace all have the desired eigenvalue of ${\hat{S}}_{z}$ and $\hat{N}$ as part of the configuration recovery procedure in the presence of noise. Conservation of ${\hat{S}}^{2}$ is more difficult to achieve in CI methods because the eigenfunctions of ${\hat{S}}^{2}$ are not SDs. The proper way of ensuring conservation of ${\hat{S}}^{2}$ is expanding $|\;\psi^{\left(k\right)}\;\rangle$ on a set of CSFs, i.e., spin symmetry–adapted linear combinations of SDs. The use of CSFs was common in early CI codes because, in addition to the obvious benefits of reducing the memory footprint of the CI vector, automatic conservation of total spin enhanced the stability of the CI iterations. Modern CI codes tend to use SDs as opposed to CSFs because the formation of the σ vector (the most memory- and rate-limiting step of CI algorithms) is considerably more efficient and easily parallelized in SD-based algorithms and in part because system memory and disk are more plentiful than in previous machines. However, in SD-based CI codes, the conservation of total spin is no longer guaranteed. In the case of SQD, sampling from a quantum computer may return sets of SDs that do not allow constructing eigenfunctions of total spin. For example, in a (2e,2o) system, one may sample the configuration ∣1001〉 (having a single spin-down excitation over the RHF state ∣0101〉), which is a linear combination of the open-shell singlet and triplet states, respectively $\left(|\;1001\;\rangle \pm |\;0110\;\rangle \right)/\sqrt{2}$. If the configuration ∣0110〉 is not sampled, one can construct neither eigenfunction of total spin, leading to spin contamination or redundancy (i.e., the configuration ∣1001〉 is involved in a CI calculation but has coefficient 0 in the CI vector). In this work, to facilitate conservation of total spin, we relied on the following procedure: Instead of collecting directly d independent and identically distributed (i.i.d.) samples from X to make the batch $S^{\left(k\right)}$, we collect $\sqrt{d}/2$ samples and identify all unique configurations $x^{u}$ (u for unique) of length M/2 obtained from $x_{↑\left(i\right)}$ and $x_{↓\left(i\right)}$ for $1\leq i\leq \sqrt{d}/2$, forming the set $U^{\left(k\right)}=\left\{\;x^{u}\;\right\}$. The size of the set is upper bounded by $|\;U^{\left(k\right)}\;|\leq \sqrt{d}$. From $U^{\left(k\right)}$, we obtain the batch set $S^{\left(k\right)}$ as

$$\begin{array}{c} S^{\left(k\right)}=\left\{\;x|x=x_{\left(i\right)}^{u}⊕x_{\left(j\right)}^{u}\text{for}\;\text{all}\;x_{\left(i\right)}^{u},x_{\left(j\right)}^{u}\in U^{\left(k\right)}\;\right\} \end{array}$$ (11)

The size of the set above is upper bounded by $|S^{\left(k\right)}|\leq d$. This procedure facilitates total spin conservation (for example, from the configuration ∣1001〉, one can build the set {∣1001〉,∣1010〉,∣0101〉,∣0110〉}, which contains two closed-shell configurations and allows constructing an open-shell singlet state) but does not enforce it. Therefore, in combination with the sampling strategy mentioned above, we achieve the conservation of total spin by a soft constraint in the eigenstate solver, i.e., by adding a penalty term to mitigate spin contamination

$$\left\{H+\lambda {\left[S^{2}-s\left(s+1\right)\right]}^{2}\right\}|\;\psi \;\rangle =E\;|\;\psi \;\rangle$$ (12)

where λ can be understood as a Lagrange multiplier that penalizes contributions from $S^{2}\neq s\left(s+1\right)$. In this work, we used a soft constraint with λ=0.2.

##### Projection and diagonalization

For each subsampled set $S^{\left(k\right)}$, the Hamiltonian is projected into the corresponding subspace spanned by the configurations in $S^{\left(k\right)}$ in Eq. 3.

We then diagonalize this projected Hamiltonian (solving ${\hat{H}}_{S^{\left(k\right)}}|\;\psi^{\left(k\right)}\;\rangle =E^{\left(k\right)}\;|\;\psi^{\left(k\right)}\;\rangle$), and its ground state forms an approximation to the ground state of $\hat{H}$. The approximate ground state $|\psi^{\left(k\right)}\rangle$ is defined by its amplitudes in the subspace

$$|\;\psi^{\left(k\right)}\;\rangle =\sum_{x\in S^{\left(k\right)}}c_{x}^{\left(k\right)}|\;x\;\rangle$$ (13)

The *k*th estimate of the ground-state energy is given by

$$E^{\left(k\right)}=\langle \;\psi^{\left(k\right)}\;|\;{\hat{H}}_{S^{\left(k\right)}}\;|\;\psi^{\left(k\right)}\;\rangle$$ (14)

#### Self-consistent configuration recovery

After a quantum state $\hat{\rho }$, corresponding to a noiseless state ∣Ψ〉, is prepared in our prefault-tolerant quantum processor, we measure it in the computational basis, obtaining the set of measurements

$$\tilde{X}=\left\{\;x\;|\;x∼{\tilde{P}}_{\Psi }\;\right\}$$ (15)

The class of noiseless states ∣Ψ〉 considered in this work are eigenstates to the total particle number operator and the total number operator for each spin species

$$\begin{array}{c} \sum_{p=1}^{N_{\text{MO}}}\sum_{\sigma }{\hat{n}}_{p\sigma }|\;\Psi \rangle =N\;|\;\Psi \rangle ,\;\sum_{p=1}^{N_{\text{MO}}}{\hat{n}}_{p↑}|\;\Psi \rangle =N_{↑}|\;\Psi \rangle ,\;\sum_{p=1}^{N_{\text{MO}}}{\hat{n}}_{p↓}|\;\Psi \rangle =N_{↓}\;|\;\Psi \rangle \end{array}$$ (16)

From the measurements on the quantum processors, we observe that there are a number of configurations in $\tilde{X}$ whose $N_{x↑}$ and $N_{x↓}$ do not match the $N_{↑}$ and $N_{↓}$ of the ground state. In table S1, we report typical values of the fraction of sampled configurations with the wrong particle number. Because the circuits we use to produce ∣Ψ〉 are particle-number preserving, we are certain that configurations with wrong particle numbers have been corrupted by noise. It is this subset of configurations that the configuration recovery scheme is applied to. The configurations in $\tilde{X}$ whose $N_{x↑}=N_{↑}$ and $N_{x↓}=N_{↓}$ are not subject to the configuration recovery subroutine.

We then probabilistically flip bits and restore the correct particle number using information obtained from observables of the system. We use the spin-orbital occupancy averaged over all collected batches of subsamples n, whose components are defined in Eq. 4.

Consider a configuration x with $N_{x↑}$ spin-up electrons, where $N_{x↑}>N_{↑}$. From the set of occupied spin-orbitals in the first half of x, $|\;N_{x↑}-N_{↑}\;|$ bits are sampled to be flipped. If instead $N_{x↑}<N_{↑}$, the bits to be flipped are instead sampled from the unoccupied spin-orbitals. The same procedure applies to the spin-down orbitals.

The probability of flipping bit $x_{p\sigma }$ depends on the distance between the value of the bit and the reference orbital occupancy $n_{p\sigma }$. The simplest approach would be to define the distribution proportional to $|\;x_{p\sigma }-n_{p\sigma }\;|$. However, this introduces an undesirable effect: If for some spin-orbital pσ, $n_{p\sigma }\approx 0.5$, then $|\;x_{p\sigma }-n_{p\sigma }\;|\approx 0.5$ as well, i.e., we assign roughly 50% probability weight to flip the bit, regardless of its initial value. On the other hand, the initial value $x_{p\sigma }$ will, in general, retain some correlation with the other values in the bitstring, even in the presence of noise. Hence, a better approach is to deweight the probability of flipping when $|\;x_{p\sigma }-n_{p\sigma }\;|$ is small by using a modified rectified linear unit (ReLU) function $w\left(|\;x_{p\sigma }-n_{p\sigma }\;|\right)$, defined as

$$w\left(y\right)=\left\{\begin{array}{c} \delta \frac{y}{h}\;\text{if}\;y\leq h\\ \delta +\left(1-\delta \right)\frac{y-h}{1-h}\;\text{if}\;y>h \end{array}\right.$$ (17)

The parameter h∈(0,1) defines the location of the “corner” of the ReLU function, whereas the parameter δ∈[0,c) defines the value of the ReLU function at the corner. w becomes a true ReLU function when δ=0, and for values of δ>0, the ReLU is modified so that it is not identically zero except at y=0 In the specific cases in this work, we chose the values δ=0.01 and h=N/M (the filling factor) in all experiments.

We do not assume that we know n a priori, and instead, we compute it and improve it self-consistently, following the procedure:

1) Setup phase: (i) Find the subset of configurations of $\tilde{X}$ that live in the correct particle sector for both spin species, which we denote by $X_{N}$: $X_{N}\subset \tilde{X}$. (ii) Obtain batches of samples $\left(S^{\left(1\right)},\ldots ,S^{\left(K\right)}\right)$ from $X_{N}$ as described in the previous section. (iii) Run the eigenstate solver on the batches and obtain approximate eigenstates $|\;\psi^{\left(1\right)}\;\rangle ,\;\ldots ,|\;\psi^{\left(K\right)}\;\rangle$ (Eqs. 3 and 13). (iv) From the approximate eigenstates construct the first guess for n, according to Eq. 4.

2) Self-consistent iterations (repeat until stopping criterion is met): (i) n is used to correct the configurations with the wrong particle number in $\tilde{X}$ (we give this subset the label $X_{/N}$). The resulting set of recovered configurations is labeled $X_{\to N}$. (ii) From $X_{R}=X_{N}\cup X_{\to N}$, batches of samples $\left(S^{\left(1\right)},\ldots ,S^{\left(K\right)}\right)$ are obtained as described in Discussion. (iii) Run the eigenstate solver on the batches and obtain approximate eigenstates $|\;\psi^{\left(1\right)}\;\rangle ,\;\ldots ,|\;\psi^{\left(K\right)}\;\rangle$ (Eqs. 3 and 13). (iv) From the approximate eigenstates construct refined guess for n, according to Eq. 4. (v) If the stopping criterion is not met, go back to step 2a.

We direct the reader to Fig. 2 in the main text for a numerical emulation of the effect of the configuration recovery on the accuracy of SQD in the presence of noise. This method is implemented in the package (*66*).

#### Energy-variance extrapolation

It is guaranteed that the accuracy of SQD (with or without configuration recovery) increases as the number of configurations used for the subspace expansion d is increased. However, the convergence of the ground-state properties with d is not expected to follow any specific functional relation. Therefore, attempting to analyze the convergence as a function of d is not well motivated. Instead, the different eigenstate approximations obtained for different values of d and different batches of samples $S^{\left(k\right)}$ are used for an energy-variance extrapolation (*43*, *67*–*71*). Consider the approximate eigenstate ∣ψ〉, whose energy is given by E=〈ψ∣H^∣ψ〉, and consider the exact eigenstate energy $E_{T}$. The difference between the approximate energy and $E_{T}$

$$\delta E=\langle \;\hat{H}\;\rangle -E_{T}$$ (18)

vanishes linearly with the Hamiltonian variance divided by the square of the variational energy (*43*)

ΔHE2=〈ψ∣H^2∣ψ〉−〈ψ∣H^∣ψ〉2E2 (19)

This linear relation is satisfied as long as ∣ψ〉 is sufficiently close to an eigenstate of the Hamiltonian, as measured by the state fidelity. The least-squares fit of a collection of energy-variance points yields an estimate of $E_{T}$, as the intersect of the fit with the ordinates. This point is the extrapolation of the estimate of the energy to the limit where ∣ψ〉 coincides with the exact eigenstate. Besides the energy extrapolation, the energy-variance analysis may also reveal the existence of multiple eigenstates close in energy to the ground state.

In the main text and in the Supplementary Materials, we apply the energy-variance analysis to two different sets of energy-variance pairs. The first one is the energy-variance pairs obtained by the HCI procedure where different energies and variances are obtained by changing the cutoff parameter that indirectly controls the number of determinants in the subspace projection and diagonalization, resulting in different levels of accuracy. The second set of energy-variance pairs are those obtained from SQD for different numbers of configurations d as well as for different batches of sampled configurations $S^{\left(k\right)}$.

### Experimental details

In this section, we describe the quantum circuits that are used to produce the configurations to which we apply the eigenstate solver. In addition, we provide details on the setting of circuit parameters from an efficient classical CCSD calculation and the mapping of the circuits to quantum processors with heavy-hex connectivity.

#### Quantum circuits

In this work, we used the LUCJ ansatz (*34*) to sample randomly distributed electronic configurations. LUCJ derives from the UCJ ansatz, which has the form (*35*) of a product of L layers, as defined in Eq. 5. We recall that

$${\hat{K}}_{\mu }=\sum_{\mathrm{pq},\sigma }K_{\mathrm{pq}}^{\mu }\;{\hat{a}}_{p\sigma }^{†}{\hat{a}}_{\mathrm{q\sigma}}\;,\;{\hat{J}}_{\mu }=\sum_{\mathrm{pr},\sigma \tau }J_{p\sigma ,q\tau }^{\mu }\;{\hat{n}}_{p\sigma }{\hat{n}}_{q\tau }\;$$ (20)

In Eq. 21, $p,q=0\ldots N_{\text{MO}}-1$ label molecular spatial orbitals and σ,τ label spin polarizations (α,β for spin-up and spin-down electrons, respectively). $K_{\mathrm{pq}}^{\mu }/J_{\mathrm{pq},\sigma \tau }^{\mu }$ has complex/real matrix elements and is anti-Hermitian/symmetric. The UCJ ansatz can be derived from a twice-factorized low-rank decomposition of the qUCCD ansatz (*35*, *72*), and the L-product form is such that the exact FCI wave function can be obtained via Eq. 5 (*35*, *36*).

The local UCJ or LUCJ (*34*) introduces a “local” approximation of the UCJ ansatz, which makes the following modifications for opposite-spin and same-spin number-number terms

$$\begin{array}{ll} \sum_{\mathrm{pq}}J_{\mathrm{p\alpha},\mathrm{q\beta}}{\hat{n}}_{\mathrm{p\alpha}}{\hat{n}}_{\mathrm{q\beta}} & \to \sum_{p\in S}J_{\mathrm{p\alpha},\mathrm{p\beta}}{\hat{n}}_{\mathrm{p\alpha}}{\hat{n}}_{\mathrm{p\beta}}\\ \sum_{\mathrm{pq}}J_{p\sigma ,\mathrm{q\sigma}}{\hat{n}}_{p\sigma }{\hat{n}}_{\mathrm{q\sigma}} & \to \sum_{\mathrm{pq}\in S\prime }J_{p\sigma ,\mathrm{q\sigma}}{\hat{n}}_{p\sigma }{\hat{n}}_{\mathrm{q\sigma}} \end{array}$$ (21)

where σ=α,β and the sets S,S′ are such that the quantum circuit implementing $e^{i{\hat{J}}_{\mu }}$ has depth O(1) and only comprises O(∣S∣+∣S′∣) number-number “*nn* gates,” i.e., two-qubit unitaries of the form $U_{\mathrm{nn}}\left(\varphi \right)=e^{-i\frac{\varphi }{4}\left(Z_{p}+Z_{q}-Z_{p}Z_{q}\right)}$, acting on adjacent qubits p,q in the topology of a certain processor. For example, on a heavy-hex processor, $S=\left\{\;4k\;,\;k=0\ldots \left(N_{\text{MO}}-1\right)/4\;\right\}$ and S′={(p,p+1),p=0…N−2} (*34*). The circuits $e^{\pm {\hat{K}}_{\mu }}$, on the other hand, can be implemented by a Bogolyubov circuit acting on $N_{\text{MO}}$ qubits and comprising $O\left[N_{\alpha }\left(N_{\text{MO}}-N_{\alpha }\right)\right]$ gates and depth $O\left(N_{\text{MO}}\right)$ (*73*–*75*). Through the local approximation, the LUCJ Ansatz balances hardware friendliness and accuracy, the latter ultimately deriving from its connection to coupled cluster theory and adiabatic state preparation (*34*).

Unless otherwise specified, we use the truncated LUCJ circuit $|\;\Psi \;\rangle =e^{{\hat{K}}_{2}}e^{-{\hat{K}}_{1}}e^{i{\hat{J}}_{1}}e^{{\hat{K}}_{1}}|\;x_{\text{RHF}}\;\rangle$, which is the result of considering the two-layer LUCJ circuit and removing the last orbital rotation and last Jastrow operations. The resulting state is implemented by a circuit whose depth is identical to the single-layer LUCJ circuit. The addition of the $e^{{\hat{K}}_{2}}$ operation to the circuit can have a large impact on the configurations generated by the circuit when the parameters are set from the $t_{2}$ tensor from a classical restricted closed-shell CCSD calculation (as described in the next section). For dynamically correlated species, the $J_{p\sigma ,q\tau }^{\mu }$ parameters obtained from $t_{2}$ can have a small amplitude, resulting in the approximate cancellation of the $\text{exp}\left(-{\hat{K}}_{1}\right)$ and $\text{exp}\left({\hat{K}}_{1}\right)$ terms in the ansatz. Without $\text{exp}\left({\hat{K}}_{2}\right)$, the resulting wave function can be overconcentrated around the Hartree-Fock configuration. Therefore, for dynamically correlated species, the action of $\text{exp}\left({\hat{K}}_{2}\right)$ is to remove some of the excessive concentration of the ∣Ψ〉 wave function, when parameters are set from a restricted closed-shell CCSD calculation.

#### Initialization of LUCJ parameters

In our experiments, we parameterize the LUCJ circuits using the following procedure:

1) First, we carry out a classical restricted closed-shell CCSD calculation, yielding amplitudes $t_{1,\mathrm{ai}}$ and $t_{2,\text{aibj}}$, where ij/ab labels occupied/unoccupied orbitals in the RHF state.

2) We reshape the $t_{2}$ tensor into the matrix ${\left(t_{2}\right)}_{\mathrm{ai},\mathrm{bj}}$ and diagonalize it, ${\left(t_{2}\right)}_{\mathrm{ai},\mathrm{bj}}=\sum_{y}\tau_{y}U_{\mathrm{ai},y}U_{\mathrm{bj},y}$, where the eigenvectors $\tau_{y}$ are sorted in decreasing order of absolute value.

3) We extend the unitaries to the following matrices

$${\tilde{U}}_{y,\mathrm{pr}}=\delta_{\mathrm{pa}}\delta_{\mathrm{ri}}U_{\mathrm{ai},y}$$ (22)

i.e., matrices where only the occupied/unoccupied block is nonzero.

4) We define the Hermitian operators

$$X_{\pm ,y}=\frac{1\mp i}{2}\left({\tilde{U}}_{y}\pm i{\tilde{U}}_{y}^{T}\right)$$ (23)

and their eigenpairs

$$X_{\pm ,y}V_{\pm ,y}=g_{\pm ,y}V_{\pm ,y}$$ (24)

5) We define the operators

$$\begin{array}{ll} {\left(J_{2y}\right)}_{\mathrm{pr}}^{\sigma \tau } & =\tau_{y}{\left(g_{+,y}\right)}_{p}{\left(g_{+,y}\right)}_{r}\\ {\left(K_{2y}\right)}_{\mathrm{pr}}^{\sigma } & =\text{log}{\left(V_{+,y}\right)}_{\mathrm{pr}}\\ {\left(J_{2y+1}\right)}_{\mathrm{pr}}^{\sigma \tau } & =-\tau_{y}{\left(g_{-,y}\right)}_{p}{\left(g_{-,y}\right)}_{r}\\ {\left(K_{2y+1}\right)}_{\mathrm{pr}}^{\sigma } & =\text{log}{\left(V_{-,y}\right)}_{\mathrm{pr}} \end{array}$$ (25)

6) We retain the first L matrices J, K. This allows us to refine a nonlocal UCJ wave function (*35*, *72*).

7) We zero out the entries of the J matrices leading to quantum gates acting on nonadjacent qubits. For a heavy-hex lattice, we retain the following elements

$$\begin{array}{l} {\left(J_{\mu }\right)}_{p,p+1}^{\alpha \alpha }\;,\;p=0\ldots N_{\text{MO}}-2\\ {\left(J_{\mu }\right)}_{p,p+1}^{\beta \beta }\;,\;p=0\ldots N_{\text{MO}}-2\\ {\left(J_{\mu }\right)}_{p,p}^{\alpha \beta }\;,\;p=0\ldots N_{\text{MO}}-1\;,\;p\%4=0 \end{array}$$ (26)

The final sparsification allows us to construct an LUCJ wave function. In a conventional LUCJ calculation, these parameters are the starting point of a variational optimization. For the hardware experiments reported in this study, we used these parameters, without further optimization, to define an LUCJ circuit, which we used to sample randomly distributed configurations.

#### Mapping to heavy-hex processors

The choice of retaining the elements ${\left(J_{\mu }\right)}_{p,p}^{\alpha \beta }$ with $p=0\ldots N_{\text{MO}}-1\;,\;p\%4=0$ when implementing LUCJ on a heavy-hex processor has an important technical motivation: On such devices, assuming a number of qubits greatly exceeding $N_{\text{MO}}$, spin-up and spin-down orbitals can be mapped on two segments of adjacent qubits forming a “zigzag” pattern and connected through an auxiliary qubit for p=0,4,8,… as shown in the three rightmost panels of Fig. 4. Such a qubit layout allows implementing LUCJ with a minimal overhead of SWAP gates (two per auxiliary qubit and layer of LUCJ). However, on current processors with up to 133 qubits, for $N_{\text{MO}}>21$ one cannot couple $N_{\text{MO}}/4$ spin-orbitals with opposite spins through auxiliary qubits without incurring a substantial overhead of SWAP gates, for the simple reason that a chain of 22 or more qubits is longer than the “diagonal” of the processor. In such a situation, as shown in the rightmost panel of Fig. 4, the segments on which spin-up and spin-down qubits are mapped form two “tails” that are not connected by auxiliary qubits. This fact has two implications: (i) no more than six spin-orbitals with opposite spins can be coupled through auxiliary qubits, and (ii) if one retains the elements ${\left(J_{\mu }\right)}_{p,p}^{\alpha \beta }$ with $p=0\ldots N_{\text{MO}}-1\;,\;p\%4=0$ and p≤16, the largest elements of $J_{\mu }$ may be discarded, yielding a lower-accuracy wave function. The first problem is a fundamental one, which can only be resolved with a substantially different mapping of fermionic degrees of freedom onto qubits and/or through the availability of larger processors. The second problem, on the other hand, has a simple solution, which we now describe. First, for any pair of spatial orbitals $p,r=0\ldots N_{\text{MO}}-1$, the orbital rotation

$${\hat{S}}_{\mathrm{pr}}=e^{-i\frac{\pi }{2}\sum_{\sigma }\left({\hat{a}}_{p\sigma }^{†}{\hat{a}}_{r\sigma }+{\hat{a}}_{r\sigma }^{†}{\hat{a}}_{p\sigma }\right)}$$ (27)

implements the permutation $S_{\mathrm{pr}}\in S_{N_{\text{MO}}}$ exchanging orbitals p and r, in the sense that

$${\hat{S}}_{\mathrm{pr}}^{†}\left(\sum_{\mathrm{qs},\tau }M_{\mathrm{qs}}{\hat{a}}_{q\tau }^{†}{\hat{a}}_{s\tau }\right){\hat{S}}_{\mathrm{pr}}=\sum_{\mathrm{qs},\tau }M\prime_{\mathrm{qs}}{\hat{a}}_{q\tau }^{†}{\hat{a}}_{s\tau }\;,\;M\prime_{\mathrm{qs}}=M_{S_{\mathrm{pr}}\left(q\right),S_{\mathrm{pr}}\left(s\right)}$$ (28)

Three immediate implications of this fact are as follows:

1) ${\hat{S}}_{\mathrm{pr}}^{†}{\hat{n}}_{q\tau }{\hat{S}}_{\mathrm{pr}}={\hat{n}}_{S_{\mathrm{pr}}\left(q\right)\tau }$

2) for any density-density operator ${\hat{J}}_{\mu }=\sum_{\mathrm{qs},\sigma \tau }{\left(J_{\mu }\right)}_{q,s}^{\sigma \tau }{\hat{n}}_{\mathrm{q\sigma}}{\hat{n}}_{s\tau }$, one has

$$\begin{array}{c} {\hat{S}}_{\mathrm{pr}}^{†}{\hat{J}}_{\mu }{\hat{S}}_{\mathrm{pr}}=\sum_{\mathrm{qs},\tau }{\left(J_{\mu }^{\prime }\right)}_{q,s}^{\sigma \tau }{\hat{n}}_{\mathrm{q\sigma}}{\hat{n}}_{s\tau }={\hat{J\prime }}_{\mu },\;{\left(J_{\mu }^{\prime }\right)}_{q,s}^{\sigma \tau }={\left(J_{\mu }\right)}_{S_{\mathrm{pr}}\left(q\right),S_{\mathrm{pr}}\left(s\right)}^{\sigma \tau } \end{array}$$ (29)

3) that for any permutation $S\in S_{N_{\text{MO}}}$ there exists an orbital rotation $e^{{\hat{K}}_{S}}$ implementing the permutation S (this is true because permutations can be written as products of exchange permutations, an exchange permutation can be implemented by an orbital rotation, and orbital rotations are closed under multiplication).

Consider now the tensor ${\left(J_{\mu }\right)}_{q,s}^{\sigma \tau }$ resulting from the low-rank decomposition of the CCSD operator described in the previous subsection. Let $p_{0}\ldots p_{\ell }$ be the ℓ elements of ${\left(J_{\mu }\right)}_{q,s}^{\alpha \beta }$ with the largest absolute values, and let $S\in S_{N_{\text{MO}}}$ be the permutation such that $S\left(p_{0}\right)=S\left(p_{0}\right)=0\ldots S\left(p_{\ell }\right)=4\ell$. Then

$$e^{-{\hat{K}}_{S}}{\hat{J}}_{\mu }e^{{\hat{K}}_{S}}={\hat{J^{\prime }}}_{\mu }$$ (30)

where the elements of ${\left(J_{\mu }^{\prime }\right)}_{q,s}^{\alpha \beta }$ with the largest absolute values are at positions 0,…,4ℓ. Before sparsifying the tensor ${\tilde{J}}_{\mu }$, one can use the identity

eK^μeiJ^μe−K^μ=eK^μeK^SeiJ^μ′e−K^Se−K^μ=eK^μ′eiJ^μ′e−K^μ′ (31)

to obtain a UCJ operator with transformed orbital rotations e−K^′μ and an opposite-spin density-density interaction whose largest elements in absolute value act on spatial orbitals p=0,4,8,…,4ℓ. Sparsification of ${\left(J_{\mu }^{\prime }\right)}_{q,s}^{\alpha \beta }$ then leads to retaining the l dominant opposite-spin density-density interaction terms (as many as allowed by the size and topology of the available heavy-hex processor) with a minimal overhead of SWAP gates.

In this work, we retained the elements ${\left(J_{\mu }\right)}_{p,p}^{\alpha \beta }$ with $p=0\ldots N_{\text{MO}}-1\;,\;p\%4=0$ and p≤16. In future work, the procedure described here could be used to modify the LUCJ wave function and the resulting probability distribution for electronic configurations.
