# Supplementary material for: Chemistry beyond the scale of exact diagonalization on a quantum-centric supercomputer
Source: Sci Adv. 2025 Jun 18;11(25):eadu9991. doi: 10.1126/sciadv.adu9991 (PMC13109957; doi:10.1126/sciadv.adu9991)
Supplement: Supplementary file 1 — Supplementary Text Figs. S1 to S20 Table S1 [file sciadv.adu9991_sm.pdf]

Science Advances
AAAS

Supplementary Materials for

Chemistry beyond the scale of exact diagonalization on a quantum-centric supercomputer

Javier Robledo-Moreno *et al.*

Corresponding author: Javier Robledo-Moreno, j.robledomoreno@ibm.com; Mario Motta, mario.motta@ibm.com; Antonio Mezzacapo, mezzacapo@ibm.com

Sci. Adv. **11**, eadu9991 (2025)
DOI: 10.1126/sciadv.adu9991

This PDF file includes:

Supplementary Text
Figs. S1 to S20
Table S1

1 Convergence properties of the components of sample-based quantum diagonalization

1.1 Wavefunction concentration and accuracy

1.1.1 Sample complexity

Under the assumption that the ground state is sufficiently concentrated, we show here that the energy obtained from the estimator converges to the ground-state energy exponentially fast in the number of samples. Suppose we sample from the ground state $|G\rangle = \sum_I c_{I(\mathbf{x})} |I(\mathbf{x})\rangle$, where $I = 0, \ldots, 2^M - 1$ label 2^M computational configurations and c_I are the corresponding coefficients. In other words, the symbol $I(\mathbf{x})$ denotes the integer with binary representation $\mathbf{x}$ for a given binary string $\mathbf{x}$. To abbreviate the notation we drop the explicit dependence of $\mathbf{x}$ on I. Let $P_I = |c_I|^2$ be the probability of each bitstring and, without loss of generality, assume the configurations are ordered by P_I: $P_0 \geq P_1 \geq P_2 \geq \ldots$. To quantify the concentration of the configurations , we define parameters α_m and β_m for a given m such that

$$\sum_{I=0}^{m-1} P_I \geq \alpha_m, \quad \text{and} \quad P_1 \geq \cdots \geq P_m \geq \beta_m. \tag{S1}$$

The first condition imposes that the total probability of the first m configurations must be at least α_m while the second lower bounds the individual P_I by β_m. We first prove general statements using α_m and β_m and, after that, apply these statements to different distributions of P_x.

If we draw N_s samples from $|G\rangle$, we can show that the probability of *not* obtaining all of the first m configurations among the N_s samples decays exponentially with N_s. The probability of not seeing a given bitstring I in any of the N_s samples is $(1 - P_I)^{N_s}$. Therefore, the probability p_{fail} of having at least one of the first m configurations not appearing in the N_s samples is upper bounded by

$$\sum_{I=0}^{m-1} (1 - P_I)^{N_s} \leq \sum_{I=0}^{m-1} (1 - \beta_m)^{N_s} \leq m e^{-N_s \beta_m}. \tag{S2}$$

Choosing N_s large enough so that $m \leq e^{N_s \beta_m / 2}$, the bound further simplifies to $p_{\text{fail}} \leq e^{-N_s \beta_m / 2}$.

Therefore, if we would like the probability of failure to be at most η, it is sufficient to choose

$$N_s \geq \frac{2}{\beta_m} \log \frac{1}{\eta}. \tag{S3}$$

Next, to understand how m should scale with the system size, we look at the error in the energy of the approximate ground state constructed from diagonalizing the Hamiltonian in the sampled basis. First, let $\mathcal{S} = \{|I\rangle : I = 0, \ldots, m-1\}$ be the truncated basis consisting of exactly the first m configurations . Let

$$|G_m\rangle = \frac{1}{\sqrt{\mathcal{N}}} \sum_{I=0}^{m-1} c_I |x\rangle \tag{S4}$$

be the normalized state constructed by truncating $|G\rangle$ to the subspace $\mathcal{S}$. Here, $\mathcal{N} = \sum_{I=1}^{m} P_I$ is a normalization constant. We have

$$\||G\rangle - |G_m\rangle\|^2 = 2 - \langle G|G_m\rangle - \langle G_m|G\rangle = 2 - 2\sqrt{\sum_{I=0}^{m-1} P_I} \leq 2 - 2\sqrt{\alpha_m}. \tag{S5}$$

Intuitively, if $\alpha_m \approx 1$, $|G\rangle$ is close to $|G_m\rangle$. In particular, it implies an upper bound on the energy of $|G_m\rangle$:

$$\begin{aligned} \langle G_m|\hat{H}|G_m\rangle &\leq \langle G|\hat{H}|G\rangle + 2\|\hat{H}\| \, \||G\rangle - |G_m\rangle\| \\ &\leq \langle G|\hat{H}|G\rangle + 2\sqrt{2}\|\hat{H}\|(1 - \sqrt{\alpha_m})^{1/2}. \end{aligned} \tag{S6}$$

Now, let $\tilde{H}$ be the $m \times m$ matrix that represents the Hamiltonian in the truncated subspace $\mathcal{S}$, i.e. the top left $m \times m$ block of H, and let $|\tilde{G}\rangle$ be the ground state of $\tilde{H}$. We have

$$\langle \tilde{G}|\hat{H}|\tilde{G}\rangle = \langle \tilde{G}|\tilde{H}|\tilde{G}\rangle \leq \langle G_m|\tilde{H}|G_m\rangle = \langle G_m|\hat{H}|G_m\rangle, \tag{S7}$$

where the inequality follows from $|\tilde{G}\rangle$ being the ground state of $\tilde{H}$. Combining Eqs. (S6) and (S7), we have a bound on the difference between the ground-state energy of $\tilde{H}$ and the original Hamiltonian H:

$$\langle \tilde{G}|\tilde{H}|\tilde{G}\rangle - \langle G|\hat{H}|G\rangle \leq 2\sqrt{2}\|\hat{H}\|(1 - \sqrt{\alpha_m})^{1/2}. \tag{S8}$$

Earlier, we assumed that $\mathcal{S}$ consists of exactly the first m bit strings. Note that $\langle \tilde{G}|\tilde{H}|\tilde{G}\rangle$ cannot increase if we add more configurations to $\mathcal{S}$. Therefore, combining with the earlier statement about the probability of seeing all m bit strings in N samples, we have that for any $\eta \in (0, 1)$, choosing

$$N_s \geq \max\left\{\log \frac{2m}{\beta_m}, \frac{2}{\beta_m} \log \frac{1}{\eta}\right\} \tag{S9}$$

guarantees

$$\langle\tilde{G}|\tilde{H}|\tilde{G}\rangle - \langle G|\hat{H}|G\rangle \leq 2\sqrt{2}\|\hat{H}\|(1-\sqrt{\alpha_m})^{1/2} \tag{S10}$$

with probability at least $1-\eta$. This result allows us to estimate the sufficient number of samples N_s given a distribution of the configurations in the ground state. For example, if $P_I \propto e^{-I}$ decays exponentially, we have $1-\sqrt{\alpha_m} \propto e^{-m}$ and $\beta_m \propto e^{-m}$. At $m \approx 2c\log M$, where c is a parameter to be determined, choosing $N_s = \Omega(M^{2c})$ is sufficient to guarantee an energy error at most $O\left(\|\hat{H}\|/M^c\right)$. We have the freedom to choose c large enough so that $\|\hat{H}\|/M^c \ll 1$. In particular, if $\|\hat{H}\| = O\left(M^4\right)$, we would choose $c > 4$. In short, the sufficient number of samples N_s scales only polynomially with the number of qubits M.

This favorable scaling persists even if $P_I \propto 1/I^\gamma$ decays algebraically for some constant $\gamma > 1$. In this case, we have $1-\sqrt{\alpha_m} \propto 1/m^{\gamma-1}$ and $\beta_m \propto 1/m^\gamma$. Therefore, choosing $N_s = \Omega(m^\gamma)$, we can guarantee the error is at most $O\left(\|\hat{H}\|/m^{(\gamma-1)/2}\right)$. Again, we have the freedom to choose $m \propto M^c$ for c large enough so that $\|\hat{H}\|/M^{c(\gamma-1)/2} \ll 1$. With this choice of m, the number of samples only needs to scale polynomially with M as $O\left(M^{c\gamma}\right)$.

Recall that in our procedure, further subsample from the set of samples $\mathcal{S}$. If the size of the subsamples is large enough, one can also prove that at least one of them contains all the m configurations . For simplicity, we assume that we will draw K subsamples uniformly from $\mathcal{S}$. This subsampling probability is the worst case for the success probability and, in practice, we would instead draw these subsamples based on their frequencies in the N_s samples. Assume that each subsample has d configurations . Additionally, we are interested in the limit $m \ll d \ll N_s$. We will calculate the probability that none of these K subsamples has all of the m correct configurations and show that this probability decays exponentially with K.

First, consider a subsample of d samples. There are $\binom{N_s}{d}$ ways to choose this subsample. Among them, $\binom{N_s-m}{d-m}$ contain the m configurations . So the probability of *not* having all m configurations is

$$p_{\text{fail}}^{(1)} \equiv 1 - \frac{d!(N_s-m)!}{N_s!(d-m)!} \leq 1 - \left(\frac{d-m}{N_s}\right)^m . \tag{S11}$$

So the probability that none of the K subsamples has all of the m configurations is bounded by

$$p_{\text{fail}}^{(K)} \leq \left[1 - \left(\frac{d-m}{N_s}\right)^m\right]^K , \tag{S12}$$

which indeed decays exponentially with K.

In the first example above where $P_I \propto e^{-I}$, if we choose $d \propto M^c$ such that $N_s/d = \kappa$ is a constant, we have

$$p_{\text{fail}}^{(K)} \lesssim \left(1 - \frac{1}{\kappa^{c \log M}}\right)^K = \left(1 - \frac{1}{M^{c \log \kappa}}\right)^K . \tag{S13}$$

Therefore, a polynomially large number of subsamples $K \propto M^{c \log \kappa}$ would ensure that the failure probability is at most a constant. Although the scaling of d with M is the same as that of N_s, choosing a large κ reduces the dimension of the matrices that we need to diagonalize. Instead of diagonalizing a large $N_s \times N_s$ matrix, we diagonalize K different $d \times d$ matrices, which can be performed in parallel.

1.1.2 Assessing wavefunction concentration

A-priori it is not possible to know if the ground-state wavefunction of a given system is concentrated. In this subsection, we present a number of tests that can be carried out to study the concentration of the ground state wavefunction, having access only to the eigenstates $|\psi^{(k)}\rangle$ produced by the estimator with or without configuration recovery.

Assume that the distribution over the space of electronic configurations generated by the wave function $|\Psi\rangle$ coincides with that of the ground state. The study of the convergence of the approximate ground state properties obtained from $|\psi^{(k)}\rangle$, as a function of d, can reveal if the ground state wavefunction is concentrated. When the ground state is not concentrated, the convergence of observables like the energy with d can be slow, or not converge at all. In the particular case of the energy, as the number of configurations d is increased, the value for the approximate ground-state energy decreases. If the approximate ground state energy has not converged when reaching the largest d amenable by the available computational resources, it is clear that the size of the support of the ground-state wavefunction exceeds the value of d.

Furthermore, when the ground state wave function is not concentrated, different batches of sampled configurations $\mathcal{S}^{(k)}$ will likely share only a few common configurations, resulting in a large variance between the properties extracted from the different $|\psi^{(k)}\rangle$.

The expectation values of observables being close to their extremal values can also be a good indicator of the concentration. For example, the closer the occupations are to a corner of the

hypercube $[0,1]^M$, the more concentrated the wavefunction is on the bitstring that corresponds to the corner.

We remark that while these tests can indicate the presence of a non-concentrated ground state wavefunction, they do not guarantee a definitive answer to whether the ground-ground state wavefunction was indeed concentrated.

1.1.3 A numerical study of the accuracy: Hubbard model

To illustrate the dependence of the accuracy of the Selected Configuration Interaction (SCI)-based eigensolver as a function of the ground-state wavefunction concentration we consider the N_{MO}-site Hubbard model in a fully-connected lattice with random hopping amplitudes:

$$\hat{H} = -\frac{1}{\sqrt{L}} \sum_{\substack{p,q=1 \\ p\neq q}}^{N_{\mathrm{MO}}} \sum_{\sigma} t_{pq}\, \hat{a}^{\dagger}_{p\sigma}\hat{a}_{q\sigma} + U \sum_{p=1}^{N_{\mathrm{MO}}} \hat{n}_{p\uparrow}\hat{n}_{p\downarrow}. \tag{S14}$$

The hopping amplitudes $t_{pq} = t_{qp}$ are independent random variables drawn from the Gaussian distribution with mean 0 and standard deviation 1, i.e. $\overline{t_{pq}} = 0$ and $\overline{t^2_{pq}} = t^2 = 1$. The results presented in this section are obtained from the average of results obtained from twenty disorder realizations. In the infinite-volume, this model is known to possess *self-averaging* properties (*77*). However, on finite-size systems, there are sample-to-sample fluctuations. The numerical study in this section does not consider the effect of noise, so we run the estimator without configuration recovery. For the discussion that follows, we span the wavefunction amplitudes in terms of electronic configurations in the localized basis, i.e. the basis used to define the Hamiltonian in Eq. (S14), contrary to the molecular systems considered where the reference basis is that of Hartree-Fock orbitals.

We choose the Hubbard model for this analysis because the ground-state properties, and the wavefunction concentration, depend on a single parameter: U. Furthermore, the study of the Hubbard model with various flavors of Configuration Interaction approaches has been considered by previous works (*78–81*). The onsite Hubbard repulsion U controls the nature of the ground state of the system. In the $N_{\mathrm{MO}} \to \infty$ limit, and for $0 \leq U < U_c$, the ground state is a Fermi liquid metal. For $U > U_c$, the ground state is a Mott insulator. The metal to insulator transition in this case is of second order (*82, 83*). *Dynamical Mean Field Theory* studies report a transition point

of $U_c = 5.82...$ (*84*). The transition on finite-size systems is harder to identify (*85*). The change in the nature of the ground state is reflected in the concentration of the wavefunction amplitudes. In the Mott phase, electrons tend to localize and there is a preference for configurations with low double occupancy (*85*). For $U = 0$ and small U, and given the disordered nature of the model, it is expected that all electronic configurations will be equivalent, especially after considering a number of disorder realizations. It is therefore expected that the eigenstate solver will show better accuracy for larger values of U.

Since the goal of this numerical experiment is to isolate the performance of SQD as a function of the wavefunction concentration, the electronic configurations used to run the SCI eigenstate solver are the $d = 10^3$ configurations of highest amplitude in the exact ground state. This choice removes any effects in the accuracy coming from the choice of the quantum circuit generating the configurations. This procedure is equivalent to the QSCI proposal (*26*), where the configurations are sampled from the exact ground-state wavefunction. As a measure of concentration, we consider the entropy of the distribution defined by the wavefunction amplitudes:

$$S = -\sum_{\mathbf{x}} |c_{\mathbf{x}}|^2 \log_2 |c_{\mathbf{x}}|^2 \,. \tag{S15}$$

To measure the accuracy in the ground state we consider the relative error in the ground-state energy, a common metric in variational many-body calculations. The relative error is refined as: $\left|E_{\mathrm{SQD}} - E_{\mathrm{exact}}\right| / E_{\mathrm{exact}}$. For the numerical experiments we consider $N_{\mathrm{MO}} = 10$, and 31 equally-spaced values of U, between $U = 1$ and $U = 16$, both included.

Fig. S1 (A) shows the relative error in the ground-state energy obtained by SQD as a function of the entropy of the distribution of the wavefunction amplitudes. The more concentrated the ground-state wavefunction (lower entropy), the lower the relative error becomes. As expected, SQD performs better for large U deep in the Mott phase. For small U in the metallic phase, the SCI trial wavefunction with $d = 10^3$ does not have enough expressive power to accurately represent the ground state of the system, whose amplitudes anti-concentrate.

Panels (B) and (C) in Fig. S1 show a comparison between the exact and SQD wavefunction amplitudes ordered by their magnitude for $U = 1$ (metal) and $U = 16$ (Mott insulator). For more concentrated wavefunctions ($U = 16$) the SQD amplitudes are in better agreement with the exact amplitudes. For $U = 1$ the number of relevant electronic configurations in the exact wavefunction

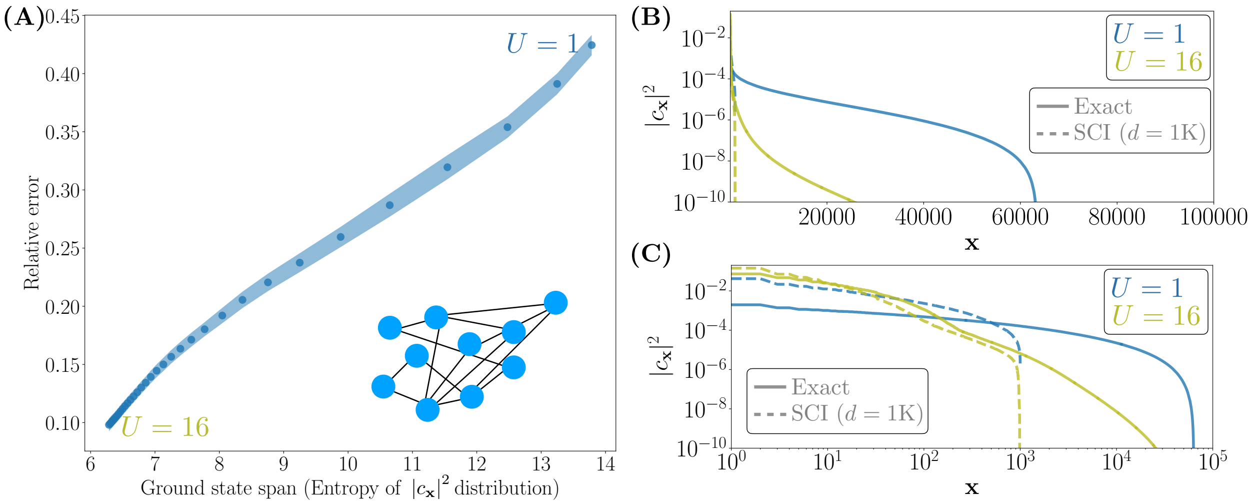

Figure S1: **Numerical study on the dependence of the performance of SCI with the ground-state wavefunction concentration.** The the fully connected Hubbard model with random hopping amplitudes as depicted in the inset of panel (A) is the subject of the study. Quantities are averaged over twenty disorder realizations. **(A)** Relative error in the ground-state energy as a function of the exact ground-state wavefunction span, as defined in Eq. (S15). Different points correspond to different values of the onsite interaction between $U = 1$ and $U = 16$ as indicated. The shaded region indicates the standard error in the mean. **(B)** Wavefunction amplitudes in decreasing order of magnitude for two values of U as indicated in the legend. Solid lines show the exact ground amplitudes while the dashed lines show the approximate ground state amplitudes obtained by SCI with 10^3 configurations (see supplementary text). **(C)** Same as **(B)** with a logarithmic scale in the horizontal axis to better show the structure of the SCI-constructed amplitudes.

greatly exceeds the $d = 10^3$ configurations that SQD is allowed to use, explaining the poor performance of SCI in the prediction of the ground-state energy as shown in Fig. S1 (A).

1.2 An analytical lower bound to the probability of recovering families of configurations

Without loss of generality, we reorder the orbital labels such that $n_1 \leq n_2 \leq \ldots \leq n_M$. Note that in the previous expression we have combined the spin-orbital multi index $(p\sigma)$ in to a single index p running from $1 \leq p \leq M$. In the following we assume that there exists a good reference configuration $\mathbf{x}_r$, obtained by assigning value 1 to the N bits of $\mathbf{n}$ which have the largest magnitude, and zero otherwise. The average deviation of $\mathbf{x}_r$ from $\mathbf{n}$ is quantified by ε: $|\mathbf{x}_r - \mathbf{n}|_1 = \varepsilon M$. ε represents the average distance between each bit in $\mathbf{x}_r$ and the corresponding entry in $\mathbf{n}$. The bits p for which $(x_r)_p = 1$ are referred to as the *1-sector* of $\mathbf{x}_r$, while the bits for which $(x_r)_p = 0$ are referred to as the *0-sector* of $\mathbf{x}_r$. For this analysis we take the probability of flipping a bit in $\mathbf{x}$ proportional to $|x_p - n_p|$, which on average takes the value:

- ε; if p is in the 1-sector of $\mathbf{x}_r$ and $x_p = 1$.
- $1 - \varepsilon$; if p is in the 1-sector of $\mathbf{x}_r$ and $x_p = 0$.
- ε; if p is in the 0-sector of $\mathbf{x}_r$ and $x_p = 0$.
- $1 - \varepsilon$; if p is in the 0-sector of $\mathbf{x}_r$ and $x_p = 1$.

We use these average values for our analytical derivations.

Let $\mathcal{U} = \mathcal{U}_L \cdots \mathcal{U}_2 \cdot \mathcal{U}_1$ denote the ideal unitary channel that produces $\psi = \mathcal{U}(|0\rangle\langle 0|)$. Here, $\mathcal{U}_i$ denotes a two qubit unitary. Let us analyze the effect of noise on $\mathcal{U}$. Suppose that each two-qubit gate $\mathcal{U}_i$ is followed by a Pauli noise channel that preserves the state with probability p, i.e, $\mathcal{P}(\rho) = p\rho + (1-p)P\rho P$ with P denoting a Pauli operator. Then after L quantum operations, the noisy output state $\widetilde{\rho}$ is given by

$$\widetilde{\rho} = p^L \rho + \sigma \,, \tag{S16}$$

where σ denotes the component of the state affected from non-identity Pauli paths.

From Eq. (S16), it follows that the number of experiments needed to sample from the ideal state ψ scales as $1/p^L$. Let $p = e^{-\lambda}$, where λ denotes the noise rate. Then the number of shots needed for sampling bitstrings from ψ scales as $e^{\lambda L}$, which is exponential in the number of noisy operations and the noise rate. In general, without performing quantum error correction, any quantum algorithm employed on noisy devices is limited to a maximum number of operations L that scales inversely proportional to the noise rate λ. Note that for our application, one might also get correct bitstrings from σ, as defined in Eq. (S16), which can decrease the number of experiments needed to obtain good bitstrings.

Above we discussed the effect of noise in obtaining samples from the ideal state. We now analyze the probability of recovering configurations using the configuration recovery scheme described earlier, as a function of their Hamming distance from $\mathbf{x}_r$. In general, this analysis depends on the noise model considered. To simplify the analysis, we consider a simple global depolarizing noise model, whose effect on the probability of sampled configurations is described by:

$$\widetilde{P}_\Psi(\mathbf{x}) = \alpha P_\Psi(\mathbf{x}) + (1-\alpha)\frac{\mathbb{I}}{2^M}, \tag{S17}$$

where $\alpha \in [0, 1]$ is a parameter that quantifies the amount of quantum signal. Note that at $\alpha = 1$, we exactly sample from the ideal state and, therefore, we do not need to perform the recovery process. While we use for simplicity a global depolarizing noise model, we expect that the analysis can be generalized to account for more realistic noise models, including the one defined in Eq. (S16).

The configurations that can be sampled from such a probability distribution fall into three categories: configurations with the right particle number that are in the support of the ideal distribution $P_\Psi(\mathbf{x})$, configurations with the right particle number that are not the support of $P_\Psi(\mathbf{x})$, and configurations with the wrong particle number. Configuration recovery does not apply to the first two categories. The third category, configurations with incorrect particle numbers, are guaranteed to have come from the noisy part of $\widetilde{P}_\Psi(\mathbf{x})$. We now derive a lower bound on the probability of drawing a configuration with wrong number of particles from $\widetilde{P}_\Psi(\mathbf{x})$ and then converting it (via the configuration recovery scheme) to a particular configuration $\mathbf{x}_{\text{target}}$ with the right particle number, whose Hamming distance to $\mathbf{x}_r$ is $2b$.

Since $\mathbf{x}_{\text{target}}$ is Hamming distance $2b$ from $\mathbf{x}_r$, it contains b 1s in the 0-sector and b 0s in the 1-sector. There are two cases for initial configurations with wrong number of particles: those that

have too many 1s or too many 0s.

1. Too many 1s. In this case only bits with the value 1 will be flipped by the configuration recovery. Consider the set of initial noisy configurations with Hamming weight $N + g + h$. Here, g is the number of 1s in the 0-sector of $\mathbf{x}_r$ in the initial configuration that need to be flipped to reach $\mathbf{x}_{\text{target}}$, while h is the number of 1s in the 1-sector that need to be flipped to reach $\mathbf{x}_{\text{target}}$. All possible combinations of g and h are considered in this analysis. Starting from the initial noisy configuration, the probability of flipping one of the g 1s that need to be flipped to reach $\mathbf{x}_{\text{target}}$ (which we will call a "successful" bit-flip) is given by

$$\frac{g(1-\varepsilon)}{(1-\varepsilon)(b+g)+\varepsilon(N-b+h)}; \tag{S18}$$

the denominator reflects the total number of 1s weighted by their probabilities of being flipped. After flipping the first 1 in the 0-sector, the probability of another successful bit-flip in the the same sector is similarly

$$\frac{(g-1)(1-\varepsilon)}{(1-\varepsilon)(b+g-1)+\varepsilon(N-b+h)}. \tag{S19}$$

Thus, the probability of flipping all g of the incorrect 1s in the 0-sector is

$$\prod_{i=0}^{g-1} \frac{(g-i)(1-\varepsilon)}{(1-\varepsilon)(b+g-i)+\varepsilon(N-b+h)}. \tag{S20}$$

The same argument can be applied to the bit-flips performed in the 1-sector. The probability of a successful first bit-flip in the 1-sector is given by:

$$\frac{h\varepsilon}{(1-\varepsilon)b+\varepsilon(N-b+h)}, \tag{S21}$$

by essentially the same argument as above. The probability of a successful second flip is

$$\frac{(h-1)\varepsilon}{(1-\varepsilon)b+\varepsilon(N-b+h-1)}. \tag{S22}$$

One can apply the same iterative argument in this case. Hence, the overall probability of obtaining the target bitstring $\mathbf{x}_{\text{target}}$ via configuration recovery is

$$\begin{aligned} &\prod_{i=0}^{g-1}\left(\frac{(g-i)(1-\varepsilon)}{(1-\varepsilon)(b+g-i)+\varepsilon(N-b+h)}\right)\prod_{j=0}^{h-1}\left(\frac{(h-j)\varepsilon}{(1-\varepsilon)b+\varepsilon(N-b+h-j)}\right) \\ &=\prod_{i=1}^{g}\left(\frac{i(1-\varepsilon)}{(1-\varepsilon)(b+i)+\varepsilon(N-b+h)}\right)\prod_{j=1}^{h}\left(\frac{j\varepsilon}{(1-\varepsilon)b+\varepsilon(N-b+j)}\right). \end{aligned} \tag{S23}$$

2. For the set of configurations with Hamming weight $N - g - h$ that came from g and h instances of $1 \to 0$ bit-flip errors in the one- and zero-sectors of the $\mathbf{x}_r$ state (respectively), a symmetric argument applies. We omit the derivation for this case.

The above gives the probabilities of recovering configurations with excess 1s or 0s back to a particular bitstring $\mathbf{x}_{\text{target}}$ of Hamming distance $2b$ from $\mathbf{x}_r$. We now integrate this probability over different values of g and h to obtain the probability of recovering a particular bitstring of Hamming distance $2b$ to $\mathbf{x}_r$, from a sample drawn from the uniform distribution. We obtain the lower bound

$$
\begin{aligned}
&P_{\text{recovery}}(M,N,b,\varepsilon) \geq \\
&\sum_{g=0}^{M-N-b}\sum_{h=0}^{N-b}\frac{\binom{M-N-b}{g}\binom{b}{h}}{2^M}\prod_{i=1}^{g}\left(\frac{i(1-\varepsilon)}{(1-\varepsilon)(b+i)+\varepsilon(N-b+h)}\right)\prod_{j=1}^{h}\left(\frac{j\varepsilon}{(1-\varepsilon)b+\varepsilon(N-b+j)}\right) \\
&+\sum_{g=0}^{N-b}\sum_{h=0}^{M-N-b}\frac{\binom{N-b}{g}\binom{b}{h}}{2^M}\prod_{i=1}^{g}\left(\frac{i(1-\varepsilon)}{(1-\varepsilon)(b+i)+\varepsilon(M-N-b+h)}\right)\prod_{j=1}^{h}\left(\frac{j\varepsilon}{(1-\varepsilon)b+\varepsilon(M-N-b+j)}\right) \\
&\equiv F(M,N,b,\epsilon).
\end{aligned}
\tag{S24}
$$

The two terms correspond to the two cases above, with the combinatorial fractions in each giving the probabilities of obtaining a configuration equivalent to the particular bitstring $\mathbf{x}_{\text{target}}$ plus g random $0 \to 1$ bit-flips in the 0-sector of $\mathbf{x}_r$ and h random $0 \to 1$ bit-flips in the 1-sector (first term), or g random $1 \to 0$ bit-flips in the one-sector of the HF state and h random $1 \to 0$ bit-flips in the zero-sector (second term). In summary, the probability of configuration recovery to output a configuration without error is lower bounded by $\alpha + (1-\alpha)F(M,N,b,\epsilon)$, where $F(M,N,b,\epsilon)$ is defined in Eq. (S24). Note that this lower bound approaches one as $\alpha \to 1$.

Fig. S2 shows the lower bound in Eq. (S24) to the recovery probability, for $\varepsilon = 0.1$ and $N = 10$, as a function of the number of qubits M. We observe that the lower bound decays as an exponential of the number of qubits and that the recovery of configurations with small values of b is more likely than the recovery of configurations defined by large b values. We remark that the configuration recovery probability for configurations close to $\mathbf{x}_r$ is substantially larger than the probability of obtaining the same samples by sampling from the uniform distribution, which would be the limit of no quantum signal. We argue that this feature helps in improving molecular energies over using simple post-selection over correct particle sector. Additionally, Fig. S2 shows the fraction

of configurations obtained from the configuration recovery procedure for different values of b on actual hardware data on the N_2 molecule on different bases sets and correspondingly different qubit numbers. The fractions are orders of magnitude larger than the lower bound and do not appear to have a strong dependence on the system size. This observation highlights that the lower bound is quite loose and in practice the performance is better.

2 Optimization of the circuit parameters

The optimization of circuit parameters to produce better distributions in the space of electronic configurations is an avenue of improvement of the SQD framework. We develop a formalism for parameter optimization using the subspace energy as the cost function, in contrast to previous experiments that rely on standard VQE optimization frameworks (*26, 27*). The HPC quantum estimator is constructed by running a diagonalization procedure in K subspaces defined by different batches of sampled configurations, as described in the main text. The number of subspaces considered K can go from $K = 1$ to $K = K_{\max}$, where $K_{\max} = \binom{D}{d}$. Recall that D is the dimensionality of the subspace of the Fock space spanned by configurations with the correct particle number and d is the dimension of the SCI subspace. Note that $K_{\max}$ is double-combinatorial in the number of spin-orbitals and electrons. Therefore, computing the HPC quantum estimator (with or without configuration recovery) for all possible sets of d configurations is not efficient. Instead, we propose the use of a Monte Carlo estimator over batches of configurations $\mathcal{S}^{(k)}$ to optimize the energy of the HPC quantum estimator averaged over different sets of configurations.

Recall that each configuration $\mathbf{x}$ is sampled with probability $P_\Psi(\mathbf{x}) = |\langle \mathbf{x}|\Psi\rangle|^2$, in the noiseless case. The following discussion applies to the noisy distribution $\tilde{P}_\Psi$. The method presented in this section can be applied also in conjunction with the configuration recovery technique. Since each configuration is i.i.d. sampled, the joint distribution that describes the probability of sampling the configurations in a batch $\mathcal{S}^{(k)}$ with d configurations is given by

$$P_\Psi\left(\mathcal{S}^{(k)}\right) = \prod_{\mathbf{x}_{(i)} \in \mathcal{S}^{(k)}} P_\Psi\left(\mathbf{x}_{(i)}\right) . \tag{S25}$$

We consider the situation where the quantum circuit that prepares $|\Psi\rangle$ is characterized by a set of variational parameters θ. The explicit dependence on variational parameters is denoted by Ψ_θ. A

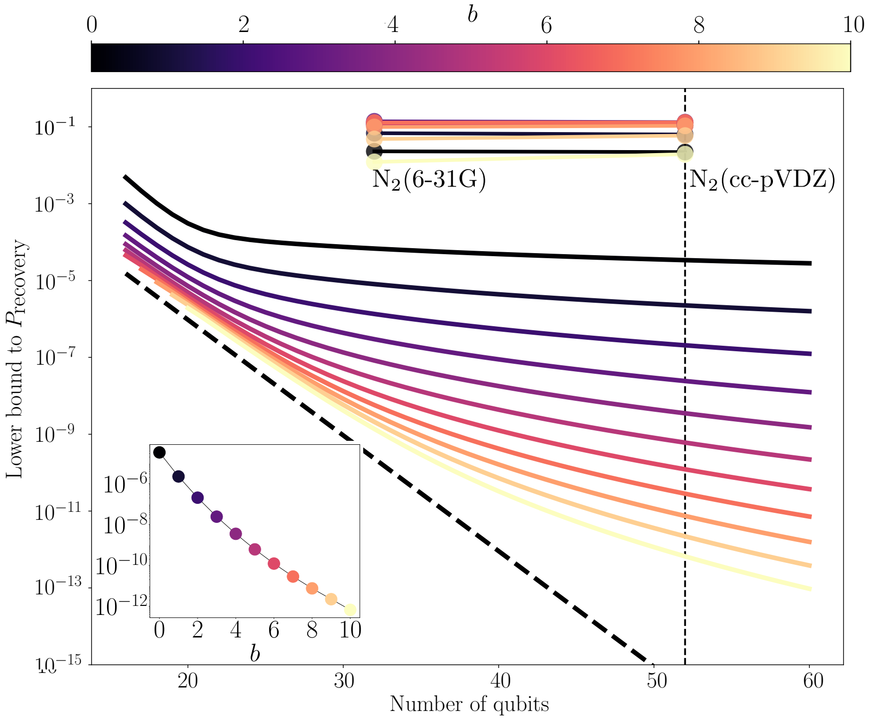

Figure S2: **Lower bound to the probability of recovering a particular bitstring with Hamming distance $2b$ from a reference approximation $\mathbf{x}_r$ bistring from a bistring sampled from the uniform distribution**. The lower bound is shown as a function of the number of qubits. Different values of b are shown, as indicated by the colorbar. The dashed black curve shows $1/2^M$, which is the probability of obtaining the desired bitsting directly from the uniform distribution. The circles connected by lines show the fraction of configurations for different families of bitstrings (as indicated by the colorbar) obtained by running the configuration recovery procedure on the samples from the quantum processor on the N_2 molecule for the 6-31-G and cc-pVDZ bases sets. The inset shows the lower bound as a function of b for $M = 52$ qubits.

cost function is formulated for the circuit optimization:

$$\mathcal{E}(\theta) = \sum_{k=1}^{K_{\max}} P_{\Psi_\theta}\left(\mathcal{S}^{(k)}\right) E^{(k)}, \tag{S26}$$

where $E^{(k)}$ is defined in the main text, Eq. (15), as the SQD ground-state energy for the configurations in $\mathcal{S}^{(k)}$. An estimator to the double combinatorially-large summation in $\mathcal{E}(\theta)$ can be obtained by the Monte-Carlo unbiased estimator:

$$\mathcal{E}(\theta) \approx \frac{1}{K} \sum_{k=1}^{K} E^{(k)} \tag{S27}$$

with $K \ll K_{\max}$ and where the $E^{(k)}$ are obtained from $\mathcal{S}^{(k)}$ sampled according to $P_{\Psi_\theta}\left(\mathcal{S}^{(k)}\right)$. The gradient of the cost function with respect to the variational parameters is given by:

$$\partial_\theta \mathcal{E}(\theta) = \sum_{k=1}^{K_{\max}} \left\{ \sum_{\mathbf{x}_{(i)} \in \mathcal{S}^{(k)}} \left[\partial_\theta P_{\Psi_\theta}\left(\mathbf{x}_{(i)}\right)\right] \left(\prod_{\substack{\mathbf{x}_{(j)} \in \mathcal{S}^{(k)} \\ \mathbf{x}_{(j)} \neq \mathbf{x}_{(i)}}} P_{\Psi_\theta}\left(\mathbf{x}_{(j)}\right) \right) \right\} \cdot E^{(k)} \tag{S28}$$

Multiplying each term in the sum from the product rule by $1 = P_{\Psi_\theta}\left(\mathbf{x}_{(i)}\right) / P_{\Psi_\theta}(\mathbf{x}_{(i)})$ we obtain:

$$\partial_\theta \mathcal{E}(\theta) = \sum_{k=1}^{K_{\max}} P_{\Psi_\theta}\left(\mathcal{S}^{(k)}\right) \left(\sum_{\mathbf{x}_{(i)} \in \mathcal{S}^{(k)}} \frac{\left[\partial_\theta P_{\Psi_\theta}\left(\mathbf{x}_{(i)}\right)\right]}{P_{\Psi_\theta}\left(\mathbf{x}_{(i)}\right)} \right) E^{(k)}, \tag{S29}$$

whose Monte-Carlo estimator is given by:

$$\partial_\theta \mathcal{E}(\theta) \approx \frac{1}{K} \sum_{k=1}^{K} \left(\sum_{\mathbf{x}_{(i)} \in \mathcal{S}^{(k)}} \frac{\left[\partial_\theta P_{\Psi_\theta}\left(\mathbf{x}_{(i)}\right)\right]}{P_{\Psi_\theta}\left(\mathbf{x}_{(i)}\right)} \right) E^{(k)} \tag{S30}$$

with $K \ll K_{\max}$ and where again the $E^{(k)}$ are obtained from $\mathcal{S}^{(k)}$ sampled according to $P_{\Psi_\theta}\left(\mathcal{S}^{(k)}\right)$. The evaluation of $\left[\partial_\theta P_{\Psi_\theta}(\mathbf{x}_{(i)})\right]$ may be achieved by:

$$\partial_\theta P_{\Psi_\theta}\left(\mathbf{x}_{(i)}\right) = \partial_\theta \left(\left\langle \mathbf{x}_{(i)} | \Psi_\theta \right\rangle \left\langle \Psi_\theta | \mathbf{x}_{(i)} \right\rangle \right) = 2\mathrm{Re}\left\{ \left\langle \mathbf{x}_{(i)} | \Psi_\theta \right\rangle \left\langle \partial_\theta \Psi_\theta | \mathbf{x}_{(i)} \right\rangle \right\}. \tag{S31}$$

We remark that the limit where $K = 1$ is equivalent to the stochastic gradient-descent optimization technique.

For most classes of variational circuits, the gradient of the circuits $|\partial_\theta \Psi_\theta\rangle$ may be implemented by parameter-shift rules or similar techniques. The reader may have noticed that the estimator for the

gradient in the cost function of Eq. (S30) is biased if the support of $\left[\partial_\theta P_{\Psi_\theta}\right]$ does not coincide with the support of P_{Ψ_θ}, as a direct consequence of writing $1 = P_{\Psi_\theta}\left(\mathbf{x}_{(i)}\right)/P_{\Psi_\theta}(\mathbf{x}_{(i)})$ for configurations outside the support of P_{Ψ_θ}, where that expression is ill-defined.

Another practical issue with the gradient-based optimization of $\mathcal{E}(\theta)$ is the requirement to evaluate $\langle \mathbf{x}_{(i)}|\Psi_\theta\rangle$ and $\langle \partial_\theta \Psi_\theta|\mathbf{x}_{(i)}\rangle$, which rely in the Hadamard test for their evaluation. The implementation of the Hadamard test requires controlled versions of the circuits that realize the variational states. The implementation of such controlled unitaries requires circuit depths beyond the reach of current quantum processors.

An alternative approach is to optimize $\mathcal{E}(\theta)$ by explicit-gradient-free methods like COBYLA (*86*) or simulated annealing. Gradient-free optimization of the circuit parameters is used in the numerical study of Sec. 3.0.2.

The optimization of the circuit parameters on quantum experiments to minimize the estimator energy will be the subject of future studies, which should consider the effect of noise in different gradient-free optimizers. Furthermore, the goal of this work is not to show that the optimization of the circuit parameters can improve the accuracy of the estimator, but to show that the quantum centric supercomputing estimator allows to tackle electronic structure problems described by an unprecedented number of qubits. Moreover, we show that the estimator run on circuits with fixed parameters already achieves good levels of accuracy.

2.1 Orbital optimization

Orbital or single-particle basis rotations are a common approach to improve the accuracy of variational calculations of electronic structure systems. Orbital optimizations can be applied in conjunction with a wide variety of electronic structure methods, including complete active space diagonalizations (*87–90*), the *density matrix renormalization group* (*91–93*), *variational Monte Carlo* (*94*) techniques, and quantum computing variational approaches (*95–97*). Moreover, orbital optimizations are common practice on SCI-based approaches as well (*57*). More generally, orbital optimizations make variational approaches invariant under orbital rotations. For notation clarity we replace in this section the notation of the two-body integral $(pr|qs)$ by the abbreviation h_{pqrs}.

The implementation of the orbital optimizations in conjunction with SQD (with or without

configuration recovery) follows the procedure presented in Ref. (*94*). Orbital rotations consist of the application of the similarity transformation:

$$\hat{\tilde{H}} = \hat{U}^\dagger(\kappa)\hat{H}\hat{U}(\kappa), \tag{S32}$$

where

$$\hat{U}(\kappa) = \exp\left(\sum_{\substack{pq\\ \sigma}} \kappa_{pq}\hat{a}^\dagger_{p\sigma}\hat{a}_{q\sigma}\right). \tag{S33}$$

In this work, we choose the matrix that parametrizes the rotation to be real: $\kappa \in \mathbb{R}^{N_{\mathrm{MO}}\times N_{\mathrm{MO}}}$. To enforce unitarity in $\hat{U}(\kappa)$ we require that $\kappa_{pq} = -\kappa_{qp}$. According to Thouless's Theorem (*98*), the action of the similarity transformation defined by $\hat{U}(\kappa)$ transforms the creation operators according to:

$$\hat{a}^\dagger_{p\sigma} \mapsto \hat{U}(\kappa)^\dagger \hat{a}^\dagger_{p\sigma}\hat{U}(\kappa) = \sum_t \Omega_{tp}\hat{a}^\dagger_{t\sigma}, \tag{S34}$$

where $\Omega = \exp(\kappa) \in \mathbb{R}^{N_{\mathrm{MO}}\times N_{\mathrm{MO}}}$. The Hamiltonian in the rotated basis can therefore be written in terms of the reference creation and annihilation operators:

$$\hat{\tilde{H}} = \sum_{\substack{pq\\ \sigma}} \tilde{h}_{pq}\hat{a}^\dagger_{p\sigma}\hat{a}_{q\sigma} + \sum_{\substack{pqrs\\ \sigma\tau}} \frac{\tilde{h}_{pqrs}}{2}\hat{a}^\dagger_{p\sigma}\hat{a}^\dagger_{q\tau}\hat{a}_{s\tau}\hat{a}_{r\sigma} \tag{S35}$$

where the one- and two-body integrals have been transformed according to the tensor transformations:

$$\begin{aligned} \tilde{h}_{pq} &= h_{tu}\Omega_{tp}\Omega_{uq}, \\ \tilde{h}_{pqrs} &= h_{tuvw}\Omega_{tp}\Omega_{uq}\Omega_{vr}\Omega_{ws}, \end{aligned} \tag{S36}$$

using Einstein's summation convention.

A variational procedure is used to search for the single-particle basis that yields the optimal description of the ground state, given the collection of all possible $K_{\max}$ *bare* variational trial states $\left|\psi^{(k)}_{\Theta}\right\rangle$. The variational procedure is similar to the method presented in Sec. 2. In this setting, $\left|\psi^{(k)}_{\Theta}\right\rangle$ is the SCI ansatz given as the linear combination of the d determinants in $\mathcal{S}^{(k)}$ (see Eq. (14)). The set of variational parameters Θ is composed of the circuit parameters θ defining $|\Psi_\theta\rangle$ together with the wavefunction amplitudes $c^{(k)}_{\mathbf{x}}$ in the subspace defined by the SCI configurations. Each variational state is *dressed* by the same single-particle orbital rotation

$$\left|\psi^{(k)}_{\{\kappa,\Theta\}}\right\rangle = \hat{U}(\kappa)\left|\psi^{(k)}_{\Theta}\right\rangle. \tag{S37}$$

The loss function to be optimized in the variational setting is defined by the average, over batches of configurations, of the Rayleigh quotient:

$$\mathcal{E}(\kappa,\Theta) = \sum_{k=1}^{K_{\max}} P_{\Psi_\theta}\left(\mathcal{S}^{(k)}\right)\left\langle \psi^{(k)}|\hat{U}^{\dagger}(\kappa)\hat{H}\hat{U}(\kappa)|\psi^{(k)}\right\rangle = \sum_{k=1}^{K_{\max}} P_{\Psi_\theta}\left(\mathcal{S}^{(k)}\right)\left\langle \psi^{(k)}|\hat{\tilde{H}}(\kappa)|\psi^{(k)}\right\rangle . \quad \text{(S38)}$$

Gradient descent and its variants can be used to minimize both κ and θ in $\mathcal{E}(\kappa,\Theta)$, while the optimization of $c_{\mathbf{x}}^{(k)}$ is carried out by the diagonalization procedure (see Sec. 1). Note that the sum in the average contains double-combinatorially many terms. Consequently, a Monte-Carlo based estimator is used to obtained an unbiased estimate (see Sec. 2). Gradients with respect to θ have the same expression as in Eq. (S29) replacing $\hat{H}$ by $\hat{\tilde{H}}$. Gradients with respect to the orbital rotations can be computed by the contraction of the bare one- and two-body reduced density matrices (1- and 2-RDMs) with the gradients of the one- and -two body integrals with respect to κ_{pq} in Eq. (S38):

$$\partial_\kappa \mathcal{E}(\kappa,\Theta) = \sum_{k=1}^{K_{\max}} P_{\Psi_\theta}\left(\mathcal{S}^{(k)}\right)\left[\sum_{\substack{pq\\ \sigma}} \tilde{h}'_{pq}\Gamma^{(k)}_{pq;\sigma} + \sum_{\substack{pqrs\\ \sigma}} \frac{\tilde{h}'_{pqsr}}{2}\left(\Gamma^{(k)}_{pqrs;\sigma,\sigma} + \Gamma^{(k)}_{pqsr;\sigma,-\sigma}\right)\right]. \quad \text{(S39)}$$

In the previous expression the gradients of the integrals in the rotated basis are given by

$$\begin{aligned} \tilde{h}'_{pq} &= h_{tu}\frac{\partial}{\partial\kappa}\left(\Omega_{tp}\Omega_{uq}\right) \\ \tilde{h}'_{pqrs} &= h_{tuvw}\frac{\partial}{\partial\kappa}\left(\Omega_{tp}\Omega_{uq}\Omega_{vr}\Omega_{ws}\right), \end{aligned} \quad \text{(S40)}$$

and the bare 1-RDMs and 2-RDMs are defined as

$$\begin{aligned} \Gamma^{(k)}_{pq;\sigma} &= \left\langle \psi^{(k)}|\hat{a}^{\dagger}_{p\sigma}\hat{a}_{q\sigma}|\psi^{(k)}\right\rangle \\ \Gamma^{(k)}_{pqsr;\sigma,\tau} &= \left\langle \psi^{(k)}|\hat{a}^{\dagger}_{p\sigma}\hat{a}^{\dagger}_{q\tau}\hat{a}_{s\tau}\hat{a}_{r\sigma}|\psi^{(k)}\right\rangle . \end{aligned} \quad \text{(S41)}$$

The gradients of the integrals with respect to the rotation parameters are computed using the automatic differentiation (AD) tools of the software package Jax (*99*). Since the evaluation of the gradients with respect to κ requires the evaluation of an intractable sum, we use an unbiased Monte-Carlo estimator for its evaluation:

$$\partial_\kappa \mathcal{E}(\kappa,\Theta) \approx \frac{1}{K}\sum_{k=1}^{K}\left[\sum_{\substack{pq\\ \sigma}} \tilde{h}'_{pq}\Gamma^{(k)}_{pq;\sigma} + \sum_{\substack{pqrs\\ \sigma}} \frac{\tilde{h}'_{pqsr}}{2}\left(\Gamma^{(k)}_{pqrs;\sigma,\sigma} + \Gamma^{(k)}_{pqsr;\sigma,-\sigma}\right)\right], \quad \text{(S42)}$$

with $K \ll K_{\max}$ and where the batches of configurations labelled by (k) are sampled according to $P_{\Psi_\theta}\left(\mathcal{S}^{(k)}\right)$ (see Eq. (S25)).

For a full orbital optimization calculation, all sets of variational parameters κ, θ, and $c_{\mathbf{x}}^{(k)}$ should be updated. Since κ and θ sets rely on gradients for their optimization, they can be updated simultaneously. However, the wavefunction amplitudes are not updated by gradients. In this case, the optimization strategy consists of the alternation of a number of gradient-descent steps optimizing κ and θ followed by the eigenstate solver that now uses samples from an updated distribution according to the change in θ with a rotated Hamiltonian according to κ. This alternation is repeated for a number of iterations N_{SCF}. In this work, we do not consider the optimization of circuit parameters θ and therefore the effect of the orbital optimization is not maximized, since the quantum circuit is not allowed to respond to the change in the Hamiltonian.

See Sec. 4.3 to see the effect of orbital optimizations in the accuracy of SQD (with configuration recovery). The section shows results run on measurement outcomes obtained from the Heron processor, to study the dissociation of N_2 at cc-pVDZ level of theory.

3 Additional information about experimental details

In this section we show examples of the structure of the compiled circuits. We show numerical experiments comparing the performance of the circuits we use against the performance of a classically efficient reduction of the LUCJ circuits.

We also describe the setup for all of the experiments conducted in this work, highlighting the quantum and classical hardware used. We show the quantum processor mappings, depth and number of gates in the circuits, and the number of measurement outcomes sampled. We also provide details about the classical computing resources.

3.0.1 Compiled circuits

In Figures S3, S4, S5, and S6, we show the specific LUCJ circuits considered in this work. Each circuit is compiled into single-qubit

$$\mathsf{U}_3(\theta, \phi, \lambda) = \begin{pmatrix} \cos(\theta/2) & -e^{i\lambda}\sin(\theta/2) \\ e^{i\phi}\cos(\theta/2) & e^{i(\phi+\lambda)}\cos(\theta/2) \end{pmatrix} \tag{S43}$$

and two-qubit CNOT gates. For readability, the displayed circuits contain barriers separating orbital rotations and density-density interactions, which we removed in the final compilation to

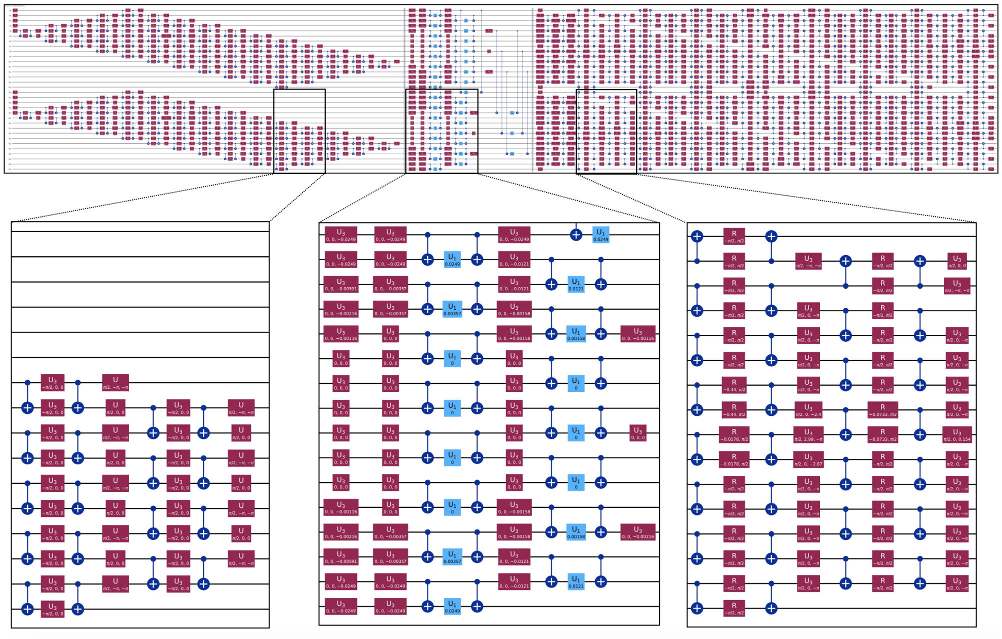

Figure S3: **LUCJ circuit used to simulate the ground state of N_2/6-31G at the equilibrium bondlength.** The circuit is compiled into single-qubit (red, purple blocks) and CNOT (light blue symbols) gates. The bottom shows zoomed-out views of gates from the first orbital rotation, density-density interaction, and second orbital rotation (left to right).

ibm_nazca/ibm_torino to reduce circuit depth. It is worth noting that the leftmost part of the quantum circuit does not implement the Bogolyubov transformation $\exp(\hat{K}_1)$, but prepares the Slater Determinant $\exp(\hat{K}_1)|\mathbf{x}_{\mathrm{RHF}}\rangle$. In other words, it reproduces the action of $\exp(\hat{K}_1)$ on a specific Slater determinant, which allows for a drastic reduction in circuit size without information loss. Subsequent orbital rotations are not amenable to such a circuit simplification.

3.0.2 Effect of the Jastrow terms in the accuracy

In this section we study the impact of the two-body terms, $\exp(i\hat{J}_\mu)$, of the LUCJ ansatz in the performance of SQD. We compare the case where the two-body parameters are zero, $J_{p\alpha,p\beta} = J_{p\alpha,p\alpha} = 0$, against the most general case where they can take arbitrary real values. Without the density-density interactions, the remaining circuit is a free-fermion evolution, i.e. the wavefunction amplitudes remain those of a single Slater determinant (*98*), so it can be efficiently simulated by a classical computer.

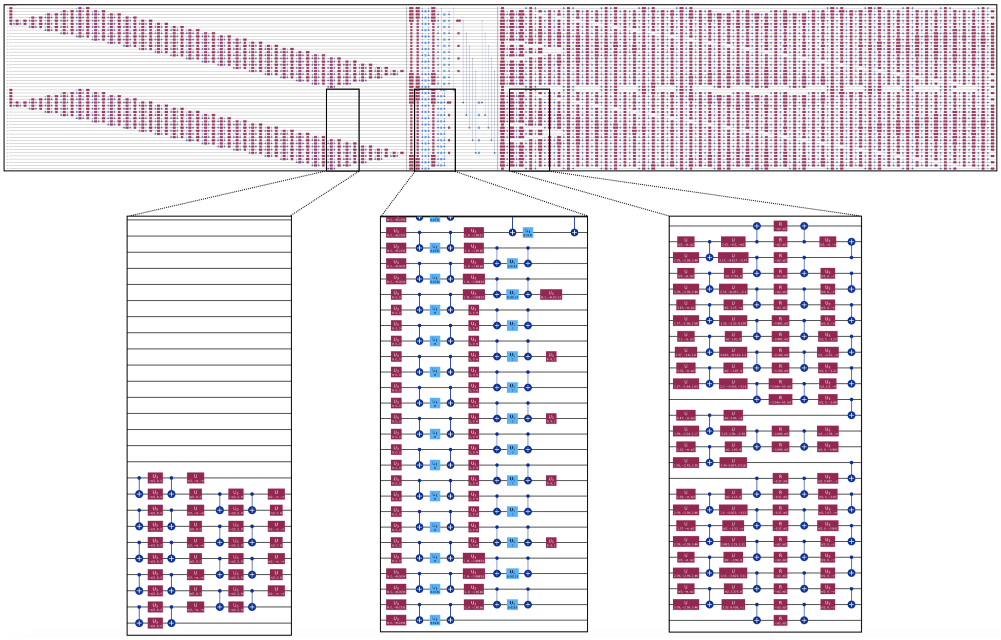

Figure S4: **LUCJ circuit used to simulate the ground state of N_2/cc-pVDZ at the equilibrium bondlength.** The circuit is compiled into single-qubit (red, purple blocks) and CNOT (light blue symbols) gates. The bottom shows zoomed-out views of gates from the first orbital rotation, density-density interaction, and second orbital rotation (left to right).

We present a numerical study of that emulates the procedure without taking into account the effect of noise. Thus, there is no configuration recovery. For the quantum circuit simulations, we used the software library `ffsim` (*38*). `ffsim` performs the state vector simulation within a subspace of fixed particle number and total $\hat{z}$ component of the spin, making the simulations much more efficient than a generic quantum circuit simulator.

Three circuits are considered: LUCJ($L = 1$), LUCJ($L = 4$), and the LUCJ circuit without two-body terms, which we refer to as “Determinant”. We study the accuracy of the estimator in the dissociation of N_2 (6-31G). For the LUCJ circuits, we consider those with heavy-hex-like connectivity in the density-density interactions; see Eq. (27). The circuits are optimized to minimize the estimator energy, as described in Sec. 2 using the COBYLA (*86*) optimizer. At each optimization step we consider $K = 1$ batches of samples obtained from the set of sampled configurations $\mathcal{X}$, where $|\mathcal{X}| = 10^7$. The value of d at each point in the dissociation curve is chosen to coincide with the number of configurations resulting from a converged HCI calculation, carried out on `PySCF`, in

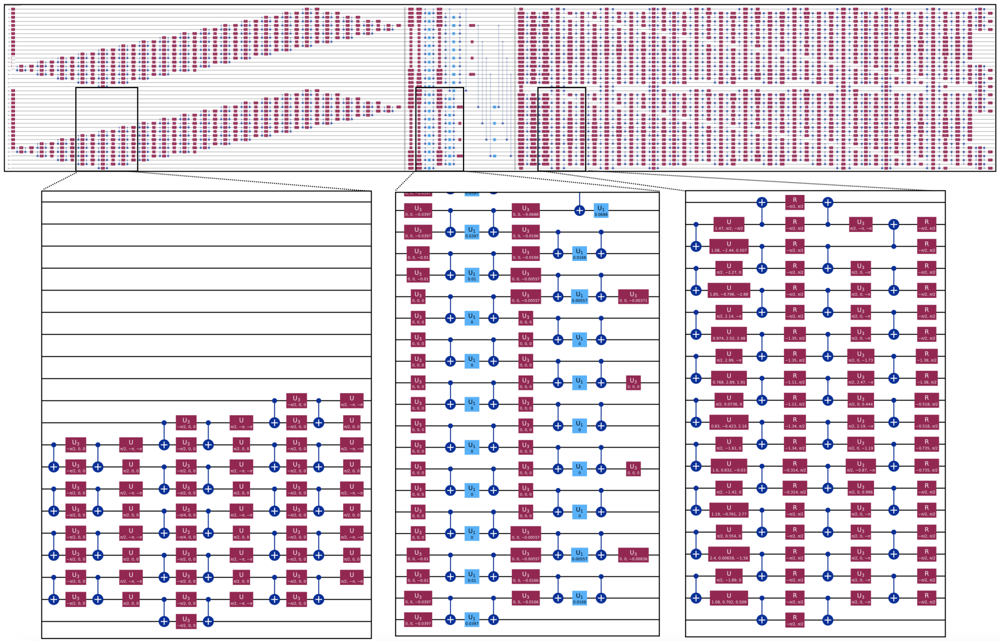

Figure S5: **LUCJ circuit used to simulate the ground state of the [2Fe-2S] cluster.** The circuit is compiled into single-qubit (red, purple blocks) and CNOT (light blue symbols) gates. The bottom shows zoomed-out views of gates from the first orbital rotation, density-density interaction, and second orbital rotation (left to right).

the same system.

Fig. S7 (A) shows the potential energy surface obtained from SQD run on configurations generated by LUCJ($L = 1$), LUCJ($L = 4$) and the determinant circuits. Panel (B) in Fig. S7 shows the energy error from the three different circuits. For most bond lengths, the error of SQD is largest for the determinant circuit, showing that the density-density interactions play a crucial role for the generation of relevant electronic configurations. This claim is also supported by the decrease of the error (on average) when increasing the number of LUCJ layers. We note that the optimization of the LUCJ circuits takes more iterations to converge than the optimization of the determinant circuits.

3.1 Quantum and classical computational resources

3.1.1 processor layouts

This section provides technical details about the experiments carried out on the quantum processors. The circuits that we use come from the truncation of two layers of the LUCJ circuits (as described

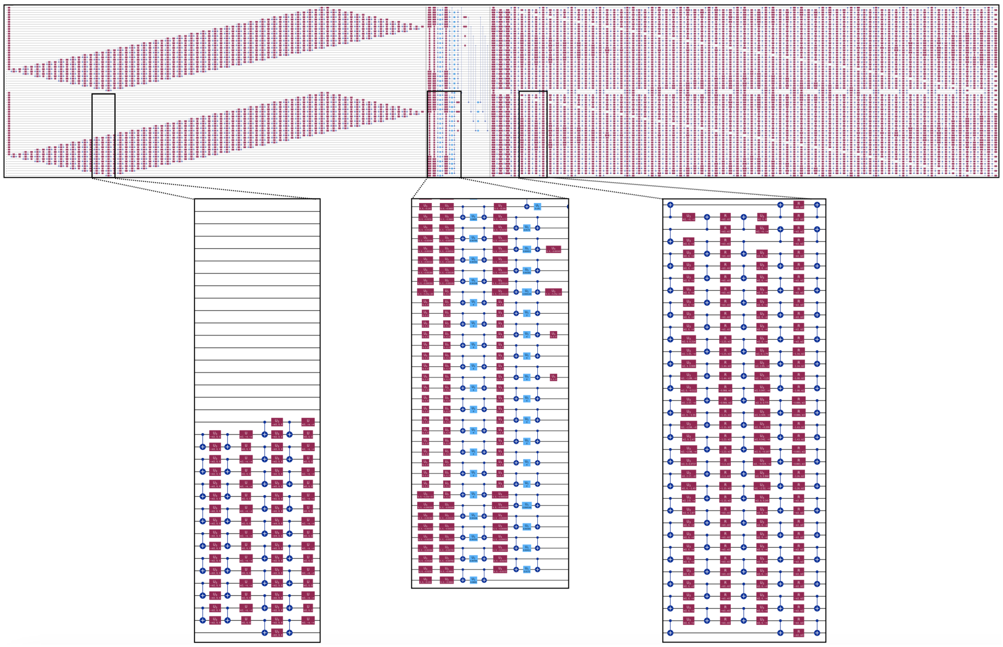

Figure S6: **LUCJ circuit used to simulate the ground state of the [4Fe-4S] cluster.** The circuit is compiled into single-qubit (red, purple blocks) and CNOT (light blue symbols) gates. The bottom shows zoomed-out views of gates from the first orbital rotation, density-density interaction, and second orbital rotation (left to right).

in Sec. 1). For the quantum runs, the circuit parameters are not optimized and instead are obtained from a classical CCSD calculation, as described in Sec. 1. Note that the circuits themselves are still classically challenging to simulate.

Table S1 lists the different molecular species, together with the number of qubits used to encode the $|\Psi\rangle$ that generates samples of electronic configurations. The same table also provides details about the total circuit depths, and numbers of one- and two-qubit gates, as well as the IBM quantum processor that was used to run the experiments. The specific qubit layouts are also provided in Table S1 and depicted in Fig. S8.

In the experiments that study the dissociation of N_2 , the number of measurement outcomes collected is $|\tilde{\mathcal{X}}| = 1 \cdot 10^5$ and $9.8304 \cdot 10^4$ per point in the dissociation curve at 6-31G and cc-pVDZ level of theory respectively. In the experiments to study the ground-state properties of [2Fe-2S] and [4Fe-4S], the number of measurement outcomes collected is $|\tilde{\mathcal{X}}|$ =2.4576 $\cdot$ 10^6. Out of the total number of measurement outcomes, a fraction of the configurations live in the subspace of the Fock

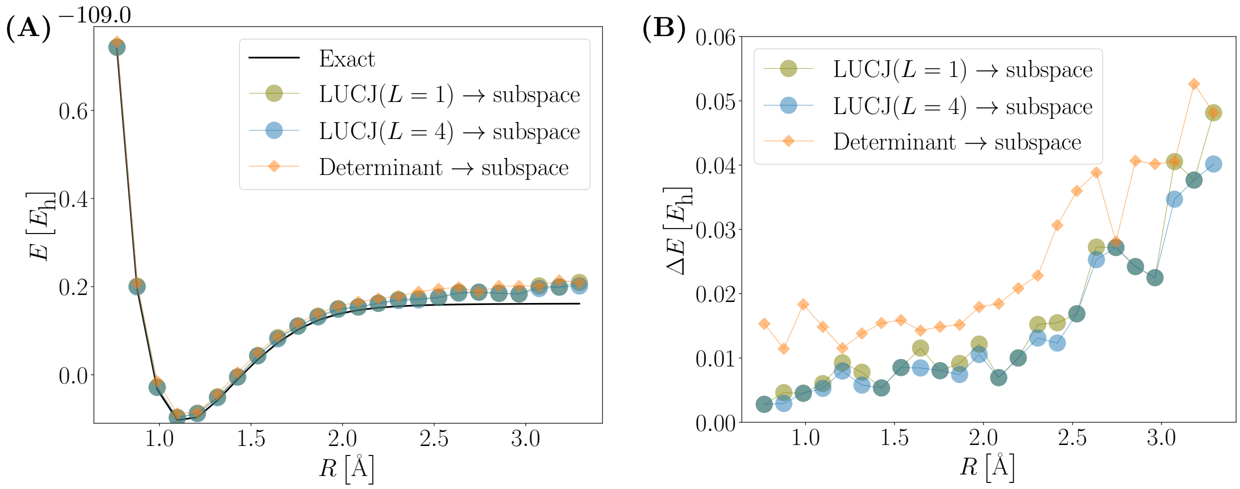

Figure S7: **Numerical and noiseless study of the accuracy of SQD obtained from different levels of approximation of the LUCJ circuit and samples obtained from an Slater determinant.** The system considered is the N_2 dissociation in the 6-31G basis. The circuits are optimized to minimize the estimator energy. In the legend, L refers to the number of layers of the LUCJ ansatz. **(A)** ground-state energy as a function of the bond length. **(B)** Difference between the exact diagonalization energy and the SQD energy as a function of the bond length.

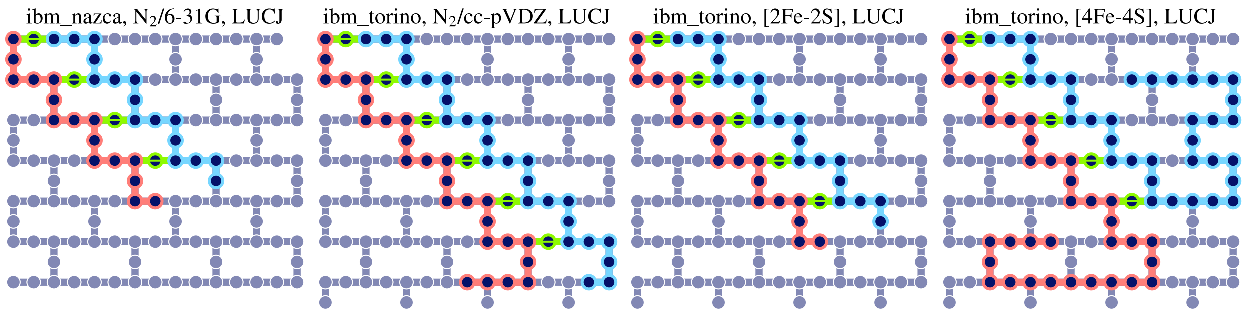

Figure S8: **Schematics of the processors used to carry out experiments.** Left to right corresponds to the N_2 molecule with 6-31G basis, the N_2 molecule with cc-pVDZ basis, the [2Fe-2S] cluster, and the [4Fe-4S] cluster, using local unitary cluster Jastrow (LUCJ) quantum circuits. Qubits used in the calculation are shown in red (for qubits associated with α spin-orbitals), blue for qubits associated with β spin-orbitals), and green (for auxiliary qubits).

space with the correct particle number, defined as

$$p_N^{\mathrm{hw}} = \frac{1}{N_s} \sum_{\ell=1}^{N_s} \delta_{N_{\mathbf{x}_\ell \alpha}, N_\alpha} \delta_{N_{\mathbf{x}_\ell \beta}, N_\beta} \ , \quad N_s = |\tilde{\mathcal{X}}| \ . \tag{S44}$$

Table S1 shows the 95% confidence interval for that fraction. Recall that the measurement outcomes that correspond to configurations with the correct particle number are used in the setup phase of the configuration recovery procedure. Table S1 also shows the fraction of sampled configurations with the correct particle number if samples were collected from the uniform distribution over configurations of length M,

$$p_N^{\mathrm{unif}} = \binom{N_{\mathrm{MO}}}{N_\alpha} \binom{N_{\mathrm{MO}}}{N_\beta} 2^{-2N_{\mathrm{MO}}} \ . \tag{S45}$$

As shown in the Table, the two probabilities are statistically distinguishable for all the experiments we carried out. Our experiments used twirled readout error mitigation (ROEM) (*100*) to mitigate errors arising from qubit measurement, and dynamical decoupling (DD) (*101–104*) to mitigate errors arising from quantum gates. We employed the implementation of ROEM and DD available on the `Runtime` library of Qiskit (*105*), through the `Sampler` primitive. DD is implemented by sequences of X control pulses, whose effect is to protect qubits from decoherence due to low-frequency system-environment coupling. Here, we applied sequences of two X pulses (as in Ramsey echo experiments) to idle qubits.

system	q (JW, Tot.)	(d, CNOT, u)	device	layout	p_N^{hw} [95% c.i.]	p_N^{unif}	$\lvert\tilde{\mathcal{X}}\rvert$
N_2, (10e,16o)	(32, 36)	(148,762,1408)	ibm_nazca	[0→82]+[2→73]	(0.0069,0.0071)	0.0044	$1 \cdot 10^5$
N_2, (10e,26o)	(52, 58)	(223,1792,3412)	ibm_torino	[0→121]+[2→127]	(0.0016,0.0017)	$9.6 \cdot 10^{-7}$	$9.8304 \cdot 10^4$
[2Fe-2S], (30e,20o)	(40, 45)	(173,1100,2070)	ibm_torino	[0→104]+[2→94]	(0.0044,0.0045)	0.00022	$2.4576 \cdot 10^6$
[4Fe-4S], (54e,36o)	(72, 77)	(301,3590,6980)	ibm_torino	[0→100]+[2→28]	($4.69 \cdot 10^{-5}$,$6.61 \cdot 10^{-5}$)	$1.88 \cdot 10^{-6}$	$2.4576 \cdot 10^6$

Table S1: Details of hardware simulations. For each active space (column 1 from the left), we list the details of the LUCJ quantum circuit executed on hardware, specifically: its number of qubits (q) both for the Jordan-Wigner encoding (JW) and the total number of qubits used including auxiliary ones (Tot.) (column 2) and count of quantum operations (circuit depth d, number of CNOT and single-qubit u gates, column 3), the device used (column 4) and the qubit layout chosen (column 5, where $[a \to b]$ indicates the shortest path between qubits a and b in the device topology, also visible in Fig. S8). In columns 6 and 7 we report a 95% confidence interval for the fraction p_N^{hw} of configurations with the correct particle number and the corresponding value for configurations with uniform probability distribution (see the text for the definition of these quantities). In column 8, we list the number of noisy configurations sampled from quantum hardware.

3.1.2 Classical resource details

The SCI-based eigenstate solver used by SQD is the one implemented in `PySCF` (*22, 61*) for the N_2 (6-31G, cc-pVDZ), and [2Fe-2S] systems. For the [4Fe-4S] molecule we use the SCI-based eigenstate solver implemented in `DICE` (*31, 34*) because of its ability to run across multiple computer nodes.

For the [4Fe-4S] experiments, the eigenstate solver can be distributed across a number of classical nodes. The workflow for [4Fe-4S] is run on the supercomputer Fugaku. The supercomputer Fugaku has a total of 158,976 nodes with Armv8.2-A SVE 512 bit architecture. Each node has 48 compute cores and 32 GiB of memory. The largest calculation in this work uses 6400 nodes of the Fugaku supercomputer running in parallel.

4 Additional experimental results

This section contains the results from the experiments that are run on the quantum processors, as described in the previous section. We show a collection of investigations that are complementary

to those shown in the main text.

We first visualize the prediction individual wave function components obtained by SQD with configuration recovery in a system size where we have access to the exact ground state wave function amplitudes (N_2 in the 6-31G basis).

We then study the scaling and runtime of the estimator. In particular, we study the effect and cost that more intensive classical resources have on the quality of the estimator. We choose the N_2 (cc-pVDZ) and [2Fe-2S] molecules for this analysis.

We also study the effect of orbital optimizations in conjunction with the HPC quantum eigensolver in the quality of the predictions. We choose N_2 in the cc-pVDZ basis as the case of study.

The results reported in Figure 4 of the main text, for the [2Fe-2S] cluster show the resolution of three different eigenstates. In this section we provide additional information about their nature. In particular, we study the average orbital occupancy for the three eigenstates in the MO and localized bases.

Lastly, we investigate the amount of signal that can be extracted and amplified from our experiments. For this study we consider the dissociation of N_2 (cc-pVDZ) as well as the study of the low-energy spectrum of the [2Fe-2S] molecule. The same study for the [4Fe-4S] molecule is in the main text.

4.1 Visualization of the wave function amplitudes

The aim of this subsection is to show the accuracy of individual wavefunction amplitudes obtained with SQD (with configuration recovery). Configuration recovery is run on measurement outcomes obtained from a quantum processor. We consider the dissociation of N_2 in a basis set that is amenable to exact diagonalization techniques but larger than a minimal basis set: the 6-31G basis. The exact diagonalization wavefunction serves as the reference to asses the accuracy of the amplitudes produced by the estimator. For each point of the dissociation curve we collect $|\tilde{\mathcal{X}}| = 100 \cdot 10^3$ measurement outcomes and run the configuration recovery procedure with $K = 10$ batches of samples. The configuration recovery method is run for ten sefl-consistent iterations. The value of the subspace dimension for the SCI eigenstate solver is $d = 4\text{M}$. For the potential energy surface we report $\min_k \left(E^{(k)}\right)$. We show the amplitudes of the wavefunction from the batch of configurations

whose energy is the lowest.

Panel (A) in Fig. S9 shows the potential energy surface of the molecule obtained by the estimator as well as the error in the estimation of the ground-state energy, as a function of the bond length. The error in the estimation of the ground-state energy remains below 10 mE_{h} for all points in the dissociation curve, thus showing good agreement with the exact results.

Panel (B) in Fig. S9 shows a comparison between the exact ground state wavefunction amplitudes and the wavefunction amplitudes obtained by the estimator, for different points in the dissociation curve. The agreement is exceptional for the larger wavefunction amplitudes and slowly deteriorates in the description of the tails of the wavefunction. We also observe that for larger bond lengths, where higher levels of static correlation are present, the agreement in the tails is worse than for bond lengths closer to the equilibrium geometry. This is to be expected for two reasons. First, for larger bond lengths the wavefunction amplitudes are less concentrated than close to equilibrium. As discussed in Sec. 1.1, the accuracy of the estimator using a SCI-based eigenstate solver deteriorates as the wavefunction becomes less concentrated. The second reason is that for bond lengths close to equilibrium, the wavefunction has a strong mean field character, but not for larger bond lengths. This directly impacts the shape of the reference spin-orbital occupancy $\mathbf{n}$ that is used to recover configurations. When the wavefunction is mean-field dominated, $\mathbf{n}$ takes the shape of a sharp step function shape with all spin-orbital average occupancies close to 0 or 1. One possible effect of static correlations is to soften the step function, moving average occupancies of some spin-orbitals away from 0 or 1. The recovery of configurations used in this work is more effective when the components of $\mathbf{n}$ are close to 0 or 1.

In summary, SQD with configuration recovery produces accurate representations of the ground state wavefunction (on experimental data), even at an individual amplitude level. Less concentrated wavefunctions tend to challenge the procedure more, both because the accuracy of the SCI solver is decreased, and because the configuration recovery becomes less effective due to softer profiles of $\mathbf{n}$.

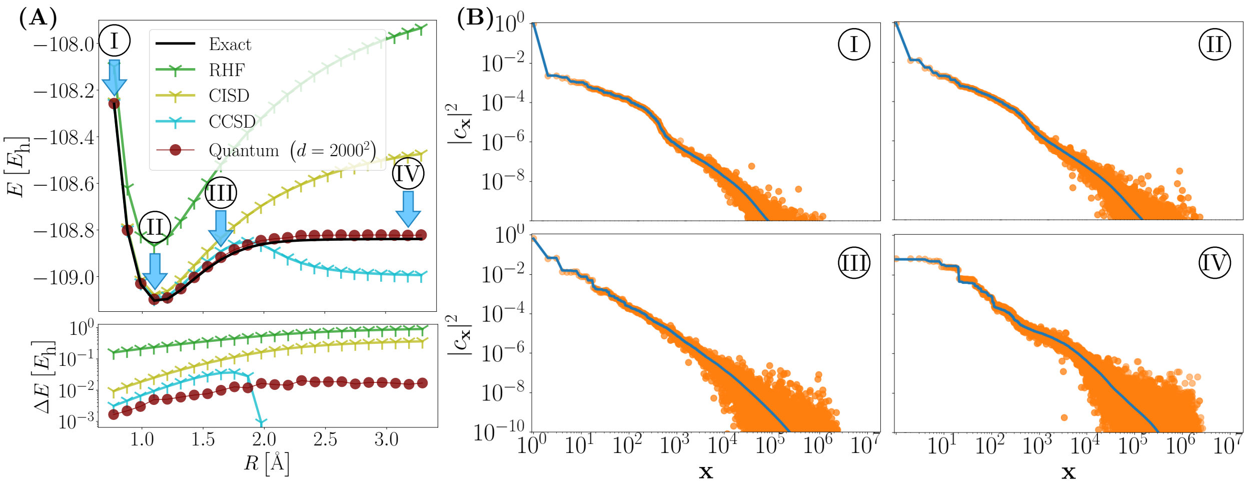

Figure S9: **Visualization of the wavefunctions produced by SQD in the dissociation of N_2 (6-31G), and the comparison against the exact wavefunctions.** **(A)** Shows the energy and energy error as a function of the bond length for the estimator with configuration recovery run from measurement outcomes from the quantum processor. Classical approximate methods are shown for reference. **(B)** Shows the wavefunction amplitudes $|c_{\mathbf{x}}|^2$ for all possible electronic configurations $\mathbf{x}$. The configurations in the horizontal axis are ordered according to the magnitude of the amplitudes in the exact wavefunction. The blue solid lines correspond to the exact ground state amplitudes while the orange dots correspond to the wavefunction amplitudes obtained from the estimator with configuration recovery. Different points in the dissociation curve are shown and labelled I, II, III, IV, following the same encoding as in panel **(A)**.

4.2 Scaling and runtime

We begin this section by studying the improvement of the accuracy of SQD with the number d of determinants used for the subspace expansion and diagonalization. It is clear that larger d yields more expressivity in the variational wavefunction, since we are increasing the possible size of the support of the state. It is also clear that increasing d increases the HPC quantum estimator runtime since both the projection and diagonalization processes become more computationally intensive. To show the improvement of the accuracy with d we consider the dissociation of N_2 (cc-pVDZ), where the measurement outcomes are generated by the quantum processor. The estimator is applied to 10^5 measurement outcomes and we take $K = 10$ batches of samples and report $\min_k \left(E^{(k)}\right)$ for the potential energy surface for different values of d. Ten self-consistent iterations are considered for the recovery of configurations.

Fig. S10 shows the potential energy surfaces obtained by the estimator with different values of d, the number of determinants used for the subspace expansion and diagonalization. Increasing the value of d improves the results both qualitatively and quantitatively. For the smaller values of d, the potential energy surface shows unphysical oscillations when the bond length is large. For larger values of d, not only the energy values are lower, but also the oscillations in the energy as a function of the bond length are substantially decreased in amplitude. Improvement of the ground state approximation with an increasing amount of computational resources is also observed in the energy-variance analyses of the main text (Fig. 4), and the supplementary materials (Figs. S19 and S20).

We analyze the vertical and horizontal scaling of the workflow, in the case where the subspace diagonalization is run on a single computer node, using the solver provided in the `PySCF` library. The [2Fe-2S] iron-sulfur cluster is used as the system of reference. Fig. S11 shows vertical scaling (increasing resources within a single node) of the eigenstate solver, and relative runtimes and schematics of horizontal scaling (adding nodes to cluster) of different blocks of the estimator (with configuration recovery). The eigenstate solver contributes the most to total runtime of the algorithm. Vertical scaling of the eigenstate solver shows noticable decrease in runtime up to a point. Experiments showed that for [2Fe-2S], 30+ CPUs per node is the optimal configuration. A good strategy for classical scaling would be to find the optimal vertical configuration and then scale

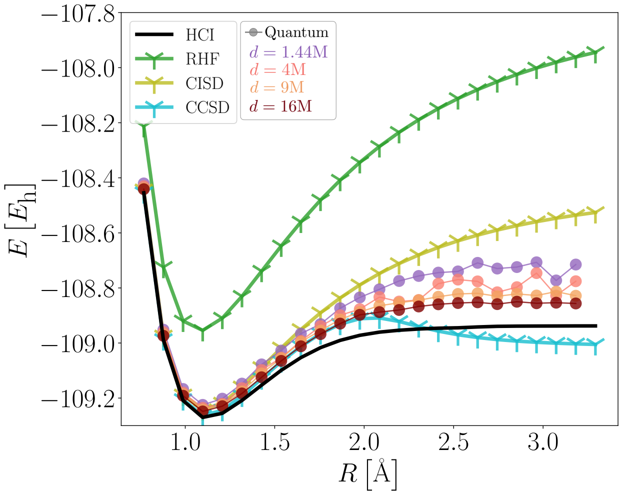

Figure S10: **Improvement of the estimator accuracy (with configuration recovery) by increasing the number of determinants for the subspace expansion and diagonalization.** Potential energy surface of the N_2 molecule (cc-pVDZ) obtained from the HPC quantum estimator estimator using measurements outcomes from the quantum processor. The energies reported correspond to the lowest energy amongst the K batches of configurations, i.e. $\min_k \left(E^{(k)}\right)$. Different subspace dimensions d are considered, as indicated in the legend. Different classical methods are shown for reference.

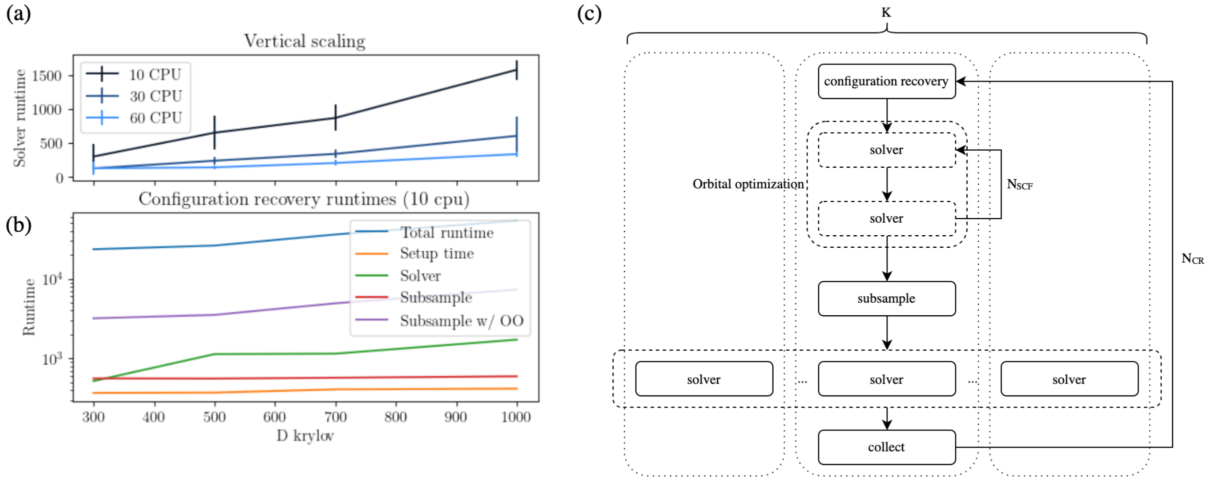

Figure S11: **Classical-resource scaling of the HPC estimator with configuration recovery and orbital optimizations. (A)** Vertical scaling of the solver (increase number of CPUs per node) for [2Fe-2S]. **(B)** Relative runtimes of blocks of the estimator for [2Fe-2S]. **(C)** Horizontal scaling of the estimator. Total runtime without horizontal scaling is $\mathrm{S} \cdot (N_{\mathrm{SCF}} + K) \cdot N_{\mathrm{CR}}$. The total runtime with horizontal scaling is $\mathrm{S} \cdot (N_{\mathrm{SCF}} + 1) \cdot N_{\mathrm{CR}}$, where S is the runtime of the solver, K is the number of nodes in a cluster, $\mathrm{N_{CR}}$ is the number of iterations, and $\mathrm{N_{SCF}}$ is the number of orbital optimization steps.

horizontally for better utilization of resources.

To conclude this section, we study the dependence of the runtime and accuracy of the estimator in the [4Fe-4S] system as a function of the amount of classical compute. The classical compute can be increased by increasing the size of the subspace dimension d, or by collecting more batches of configurations K. The `DICE` library allows to distribute the subspace diagonalization across a number of independent computer nodes. It is expected that more classical nodes will yield faster runtimes per diagonalization. For this experiment the Heron processor Montecarlo is used, with $|\mathcal{X}| = 3163742$ collected measurement outcomes, and $p_N^{hw} = 5.4 \cdot 10^{-4}$.

Fig. S12 (A) shows the runtime of the diagonalization of a single batch of configurations as a function of the number of classical nodes. Different subspace dimensions are considered. As expected, with an increased number of nodes, the runtime of the diagonalization decreases. After a certain number of nodes, the speedup saturates, due to the overhead in inter-node communications. This panel allows us to identify the number of classical nodes necessary to have a fixed runtime

for the diagonalization for different subspace dimensions. To study improvement of the accuracy of the estimator with the number of batches of configurations K, we run the workflow with $K = 100$. From the $K = 100$, batches, we subsample the energy of smaller groups of batches and collect the minimum energy for each subsample. The subsampled minimum energies are averaged. Different sizes of the smaller groups of batches are considered until reaching the group of $K = 100$ batches of configurations. Panel (B) in Fig. S12 shows the average of the minimum energies as a function of the total number of nodes for different values of d, such that the runtime of each digonalization is fixed to 1.44 hours. The total number of classical nodes is given by the product of the size of the groups of batches of configurations and the number of nodes required to obtain a runtime of 1.44 hours per subspace projection and diagonalization.

4.3 Effect of orbital optimizations in the dissociation of N_2 (cc-pVDZ)

We apply orbital optimizations (OO) to improve the accuracy of SQD, in the study of the dissociation of N_2 (cc-pVDZ). The estimator with configuration recovery and its orbital-optimized counterpart are applied to the same set of noisy measurement outcomes $\tilde{\mathcal{X}}$ obtained from the quantum processor. We use $K = 10$ batches of samples in each case and $N_{\text{SCF}} = 10$ iterations of the alternation between the optimization of κ and running of the eigenstate solver to update $c_{\mathbf{x}}^{(k)}$. After each $c_{\mathbf{x}}^{(k)}$ update, 5000 iterations of gradient descent with momentum are used to optimize κ. Before applying orbital optimizations, the configuration recovery procedure is run for 10 iterations on the reference basis (molecular orbitals). Various sizes d of the batches are considered.

Fig. S13 compares the ground-state energy obtained by the estimator without and with orbital optimizations. We observe that close to the equilibrium bond length the effect of the orbital optimizations is negligible. However, upon dissociation, the estimator with orbital optimizations obtains noticeably lower ground state energies. For smaller values of d, the estimator without orbital optimizations shows unphysical oscillations in the potential energy surface. The orbital optimizations decrease the amplitude of those oscillations.

In summary, orbital optimizations allow finding the single-particle basis in which the estimator is most accurate, thus improving the quality of its predictions. Furthermore, the optimization of the circuit parameters θ would allow the circuit to respond to the change of basis to produce electronic

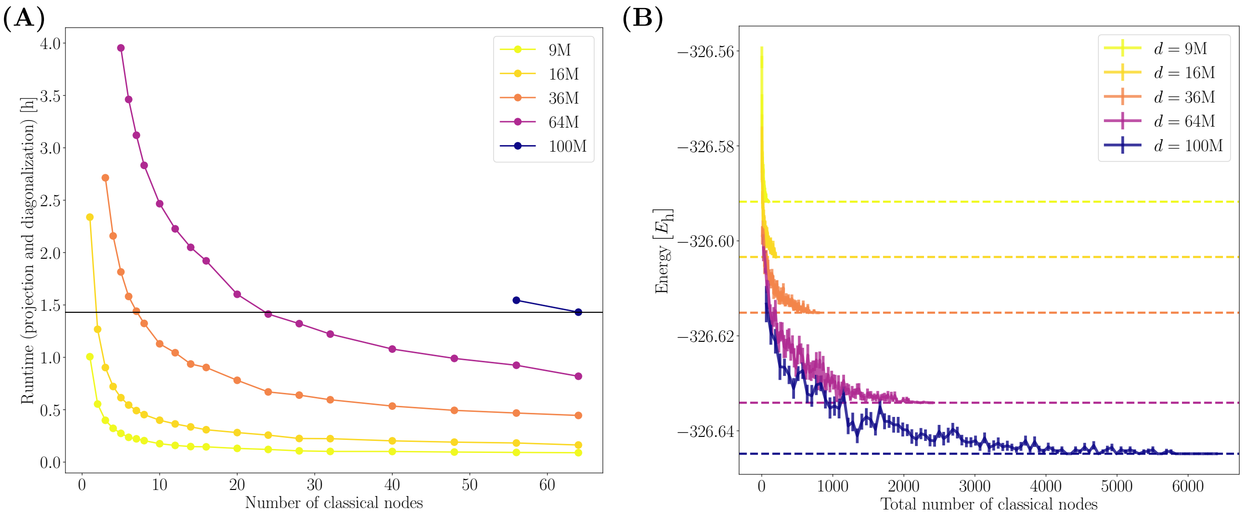

Figure S12: **Accuracy and runtime of SQD for the [4Fe-4S] iron-sulfur cluster.** **(A)** Runtime of a single subspace projection and diagonalization as a function of the number of nodes that are used to distribute the calculation. Different colors correspond to different subspace dimensions. The smallest number of nodes per curve corresponds to the limit where the calculation runs out of memory on a single node. The subspace projection and diagonalization are handled by the `DICE` library. The horizontal line shows a fixed runtime of 1.44 hours for the different subspace sizes. **(B)** Statistical convergence of SQD as the number of batches of samples K is increased. The vertical axis shows the the average minimum energy obtained by a fixed number of batches of samples. Each batch of samples is run on fixed number of nodes and the diagonalization for the different batches are run in parallel. The number of nodes for the diagonalization of a single batch is chosen to obtain a fixed runtime of 1.44 hours for all of the different subspace dimensions d. The horizontal axis shows the total number of nodes corresponding to different numbers of batches. The largest number of batches corresponds to $K = 100$.

configurations better suited for the new basis. As mentioned earlier, the optimization of the circuit parameters will be the subject of follow-up projects. Note that the unrestricted optimization of κ can break symmetries that may be desirable to preserve, so it is up to the user to decide whether to use orbital optimizations or not.

4.4 Orbital occupation numbers for the low-energy spectrum of [2Fe-2S]

The calculations reported in Figure 4 of the main text, for the [2Fe-2S] cluster, identified three distinct eigenstates that we labeled A, B, and C. In this section, we provide additional information about the nature of the three identified eigenstates. The purpose of this analysis is to illustrate the robust classification of the wavefunctions produced by our estimator into three families (A, B, and C) but also to shed light on the nature of the corresponding eigenstates and the ability of our method to accurately capture a multireference character in electronic eigenstates, pinpointing areas of possible future improvement. In particular, we show the spatial orbital occupancy for the three eigenstates that SQD finds for [2Fe-2S] both in the MO basis and in a basis of spatially local orbitals. The MO basis helps highlight deviations from the mean-field states or lack thereof, and the basis of localized orbitals (identifiable with e.g. Fe $3d$ and S $2p$ orbitals) helps characterize our method's ability to capture local antiferromagnetic correlations between transition-metal atoms.

The spatial orbital occupancy is given by $n_p = \sum_\sigma n_{p\sigma}$. Therefore $n_p \in [0, 2]$. For Eigenstate A, the occupancy comes from the estimator run with $d = 9$M. For Eigenstates B and C, the occupancy comes from the estimator run with $d = 16$M. We cannot compare the occupancy for the same value of d since the description of eigenstates A and B require different numbers of configurations (see Sec. 4.5.2 and Fig. S20). For Eigenstates B and C, we use the same number of determinants to make the comparison as even as possible. The number of batches of configurations is $K = 10$.

Fig. S14 shows the n_p profile for the three eigenstates identified from the energy-variance analysis, in the basis of MOs. The occupancy profile for Eigenstate A is the typical profile of an eigenstate with a strong mean-field character. The occupancy of the lowest-energy $N_\sigma = 15$ molecular orbitals is close to its maximum value, while the remaining orbitals have lower occupancy. The character of the occupancy profile for Eigenstate B is different from that of Eigenstate A. The occupancy of the third and fourth orbitals is depleted to give a more prevalent presence of electrons

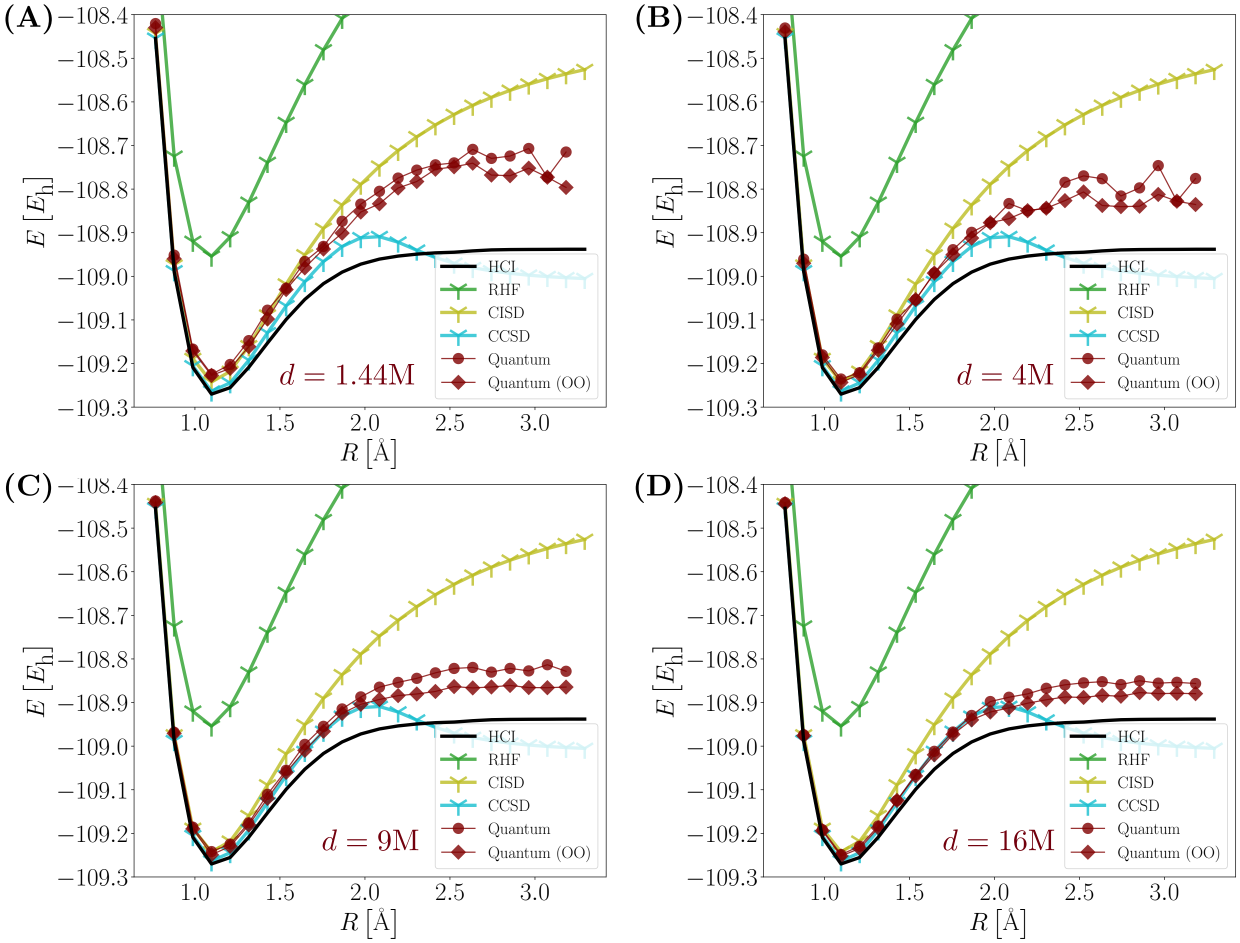

Figure S13: **Effect of orbital optimizations (OO) in the performance of SQD with configuration recovery in the dissociation of N_2 (cc-pVDZ).** The ground-state energy is shown as a function of the bondlength. The estimator is run on measurement outcomes from a quantum processor. The energies shown correspond to the lowest energy amongst the K batches of configurations, i.e. $\min_k \left(E^{(k)}\right)$. Energies from different classical methods are shown for reference, as indicated in the legend. Results are shown for the estimator run with different numbers of configurations d, as indicated in each panel **(A)**-**(D)**.

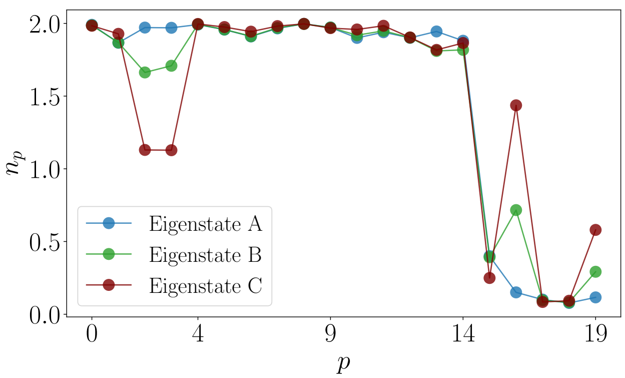

Figure S14: **Orbital occupancy profile n for the [2Fe-2S], produced by SQD with configuration recovery.** The increasing index p labels molecular orbitals of increasing energy. The occupancy profile is shown for the three eigenstates identified in the energy-variance analysis (see Fig. 4 (B) in the main text).

in orbitals $p = 16$ and $p = 19$. This effect is even more noticeable for eigenstate C, where the occupancy of the third and fourth orbitals is depleted by half and the occupancy of orbital $p = 16$ is now over the half-filling factor.

To further understand the nature of Eigenstates B and C, we study the occupancy in the basis of localized orbitals $\tilde{\mathbf{n}}$ (see Sec. 1 for details about the basis of localized orbitals). The reported occupancies are constructed as follows: first, we obtain the one-body density matrix for the approximate ground state obtained from each batch of configurations, expressed in the basis of MOs: $\Gamma^{(k)}_{pq,\sigma}$ (see Eq. S41). We then rotate the density matrix to the basis of localized orbitals with a similarity transformation denoted by Ω,

$$\tilde{\Gamma}^{(k)}_{tu,\sigma} = \Gamma^{(k)}_{pq,\sigma}\Omega_{pt}\Omega_{qu}, \tag{S46}$$

where $\tilde{\Gamma}^{(k)}_{tu,\sigma}$ is the one-body density matrix in the basis of localized orbitals. The occupancy of localized orbital t is given by

$$\tilde{n}_t = \frac{1}{K}\sum_{k=1}^{K}\sum_{\sigma}\tilde{\Gamma}^{(k)}_{tt,\sigma}. \tag{S47}$$

Fig. S15 shows a comparison of the value of $\tilde{\mathbf{n}}$ for Eigenstates A, B, and C. The reflection symmetry along the plane that separates the left and right sides of the molecule is broken for the three approximate eigenstates. This is highlighted by the observation that the occupancy of the equivalent orbitals for the two Fe atoms are not the same. We also observe that the occupancy of

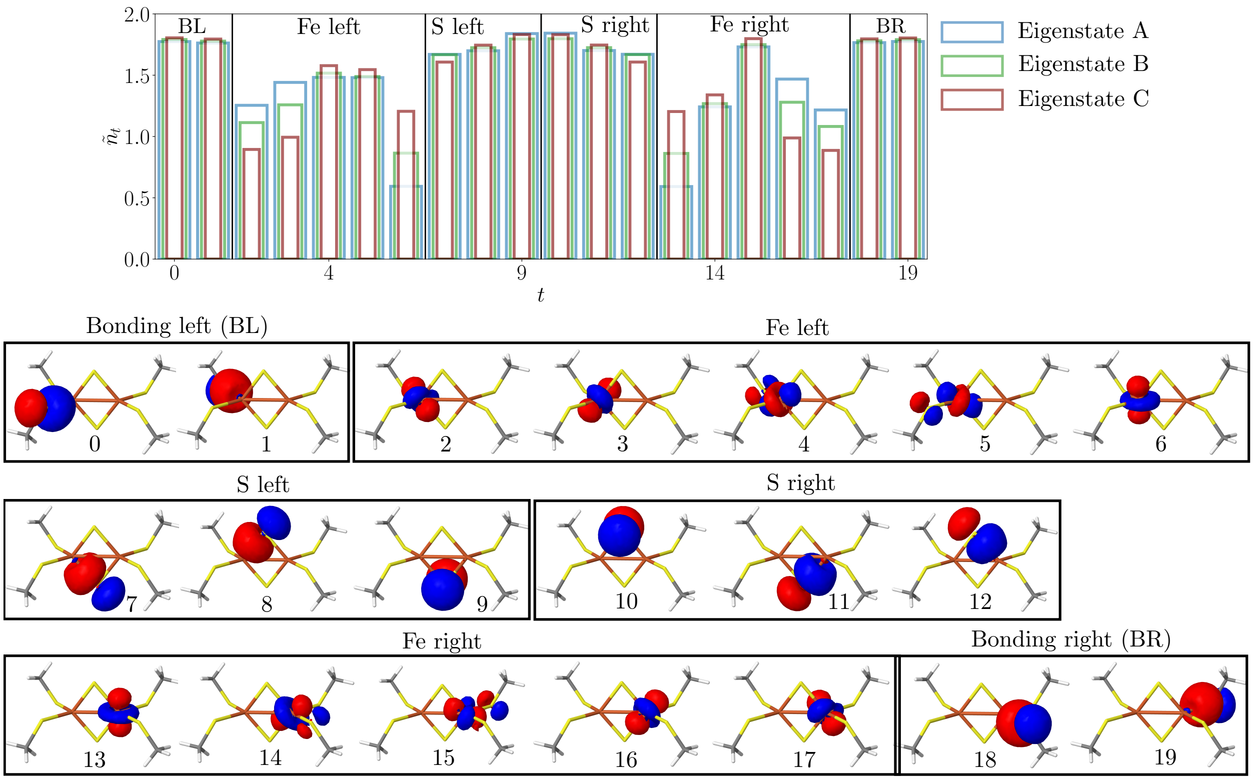

Figure S15: **Orbital occupancy profile ñ for the [2Fe-2S], produced by SQD with configuration recovery.** The increasing index t labels localized orbitals as shown in the bottom of the panel. The occupancy profile is shown for the three eigenstates identified in the energy-variance analysis (see Fig. 4 (B) in the main text). The representation of the orbitals is obtained from the repository (*106*) from Ref. (*41*).

the bonding orbitals of the SCH_3 groups and the occupancy of the $3p$ orbitals of the S atoms is similar for the three eigenstates. The occupancy of the $3d$ orbitals of Fe has the largest differences among the three different eigenstates. The more notable discrepancy in the d-orbitals of Fe may be a consequence of the fact that the low-energy physics of this molecule is dominated by the antiferromagnetic coupling of the electrons in the Fe $3d$ orbitals (*41, 43*).

4.5 Quantum signal in the experiments

In this section we assess the quality of the quantum signal for all our experiments, going beyond the [4Fe-4S] analysis presented in the main text.. Given the large circuit sizes of our experiments:

- [2Fe-2S]: 40 qubits (not including auxiliary), 1100 two-qubit gates, 3170 total gates,
- N_2 : 52 qubits (not including auxiliary), 1792 two-qubit gates, 5204 total gates,
- [4Fe-4S]: 72 qubits (not including auxiliary), 3590 two-qubit gates, 10570 total gates,

one should verify whether there is a useful signal coming out of the quantum circuits, comparing samples from $\tilde{P}_\Psi$ and uniform random samples. We first investigate the fraction of sampled configurations that live in the correct particle sector p_s^{hw} and compare it to the fraction that would be obtained if the samples came from the uniform distribution p_s^{unif}. The values for p_s^{hw} alongside their 95% confidence interval, and p_s^{unif} are reported in Table S1. For the 40-, 52-, and 72-qubit experiments, we observe that p_s^{hw} is ~ 20, $\sim 10^3$, and ~ 40 times larger than p_s^{unif} respectively. This provides a first indication that configurations sampled from the quantum processor contain a signal that is distinguishable from white noise.

The second set of tests that we conduct is the comparison of the ground-state energy obtained by SQD with and without applying the configuration recovery procedure. Note that not applying configuration recovery makes the estimator equivalent to the QSCI framework (*26*) . Having reasonable energies (lower than or in the vicinity of the RHF energy) without applying configuration recovery reveals that the set $\mathcal{X}_N$ already contains reasonable configurations to construct a non-random ground state.

The third set of tests involves comparing the performance of SQD with configuration recovery run on configurations obtained from the quantum processor and samples obtained from the uniform distribution. We consider the uniform distribution over all possible configurations in the Fock space, as well as the uniform distribution over configurations with the correct particle number, as in Fig. 4 (C) in the main text. When considering samples from the uniform distribution over electronic configurations on the correct particle sector, the configuration recovery method cannot be applied since it only recovers configurations with the wrong particle number. The results for these last two sets of tests are shown in the coming subsections for the 40-, 52- and 72-qubit experiments.

4.5.1 N_2 experiments

Effect of configuration recovery on the accuracy of the HPC quantum estimator.– In this section, we compare the accuracy of the estimator without configuration recovery (applied to

$\mathcal{X}_N \subset \tilde{\mathcal{X}}$) to the accuracy of the estimator with configuration recovery (applied to $\tilde{\mathcal{X}}$). As for all N_2 experiments, $|\tilde{\mathcal{X}}| = 100 \cdot 10^3$. From the experimental measurements, we observe that the average (over the bondlengths considered in this study) size of the $\mathcal{X}_N$ set is $|\mathcal{X}_N| = 165$. The maximum number of determinants that can be extracted to perform the projection and diagonalization is $d_N^{hw} = 110$K. Since typical values of d used for the N_2 molecule exceed d_N^{hw} we perform the projection and diagonalization using all the configurations obtained from $\mathcal{X}_N$ (number of batches of configurations is $K = 1$). For the estimator run with configuration recovery we use $K = 10$. In both cases, we report the $\min_k \left(E^{(k)}\right)$ energy for the potential energy surface. When running the estimator with configuration recovery we use 10 self-consistent recovery iterations.

Fig. S16 shows the comparison between SQD run with and without configuration recovery. The ground-state energy is shown as a function of the bond length. The estimator run on $\mathcal{X}_N$, i.e. without configuration recovery, obtains energies comparable to RHF energies. A level of accuracy comparable to RHF is not desirable for correlated electronic structure methods. However, since no mitigation techniques have been used in the large circuits producing the samples, this is an indication that a useful quantum signal (mostly of mean-field character) is present in $\tilde{\mathcal{X}}$. As expected, the estimator with configuration recovery shows higher levels of accuracy as compared to not using configuration recoivery, obtaining a qualitatively correct dissociation curve upon increasing d.

The accuracy of the energies obtained by SQD without configuration recovery could be improved by obtaining more measurement outcomes and thus more configurations on the right particle sector. The typical values of d used for the estimator with configuration recovery are larger than d_N^{hw}. With current noise rates, obtaining a larger size of $|\mathcal{X}_N|$ to run the estimator without configuration recovery, requires the collection of many more measurement outcomes, at an efficiency where approximately only 1 in 1000 measured configurations has the right particle number (see Tab S1 for the precise fraction). Configuration recovery allows improving the accuracy of SQD without the collection of more measurement outcomes from the quantum processor. This is desirable as it reduces the overall cost of the calculation.

Comparison of the quality of the estimator with configuration recovery, applied to quantum data and configurations drawn from the uniform distribution.– We first compare the performance of the estimator with configuration recovery applied to measurement outcomes from the

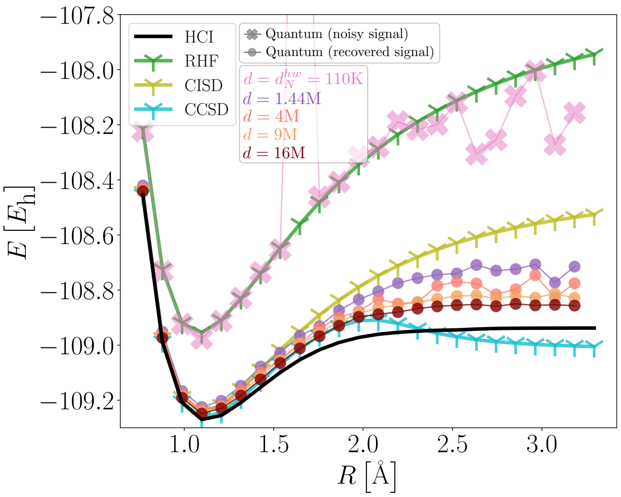

Figure S16: **Comparison of the performance of SQD, with and without configuration recovery, run on the same set of measurement outcomes from the quantum processor.** The dissociation of N_2 (cc-pVDZ) is considered. We compare the performance of the estimator with no configuration recovery (crosses) to the performance of the estimator with configuration recovery (points). Different numbers of configurations d are considered, labeled by different colors. d_N^{hw} refers to the number of configurations on the right particle sector of N electrons extracted from the measurement outcomes, used for the estimator without configuration recovery (see the text for more details). We note that with current noise rates, $d_N^{hw} < 1.44$ M. Different classical methods are shown for reference, as indicated in the legend.

quantum processor versus its application to configurations sampled from the uniform distribution in the right particle sector. The estimator is applied to 10^5 samples in both scenarios and we use the same value of d. In both cases, we take $K = 10$ batches of samples and report $\min_k \left(E^{(k)}\right)$ for the potential energy surface. When running the estimator with configuration recovery we use 10 self-consistent iterations. Recall that the configuration recovery procedure recovers configurations that have the wrong particle number due to noise, thus the application of the estimator to configurations sampled from the uniform distribution in the right particle sector is equivalent to applying the SCI eigenstate solver to the random configurations. Panel (A) in Fig. S17 shows the comparison. We observe that the potential energy surface obtained from the measurement outcomes from the quantum processor is both qualitatively and quantitatively more accurate than the one obtained from the uniform distribution over configurations in the right particle sector. The potential energy surface obtained from the configurations sampled from the uniform distribution shows a very prominent non-smooth behavior with energies worse than the RHF energies for a number of bond lengths.

The comparison with samples obtained from the configurations uniformly sampled over the full Fock space and the quantum samples is shown in Fig. S17 (B). We remark that 10^5 samples over the uniform distribution in the Fock space of electronic configurations are unlikely to reveal any configurations in the correct particle sector, since the probability of a uniformly random bitstring falling in the correct particle sector is $\binom{N_{\mathrm{MO}}}{N_\uparrow}\binom{N_{\mathrm{MO}}}{N_\downarrow}/2^M = 9.6 \cdot 10^{-7} < 10^{-5}$. Recall that the configurations on the right particle sector are required to start the configuration recovery procedure by providing the initial average occupancies. Therefore, we take $50 \cdot 10^6$ configurations sampled from the uniform distribution over configurations of length M for this comparison. Note that the use of more samples from the uniform distribution as compared to the number of measurement outcomes from quantum constitutes a comparison that favors the uniform distribution scenario.

In spite of this, Fig. S17 (B) shows a higher level of accuracy obtained by the estimator with configuration recovery run using measurement outcomes from the quantum processor. These results indicate that the quantum processor has a signal that is amplified by the configuration recovery procedure and used by SQD to procedure more accurate ground-state representations than running the same estimator on uniform noise.

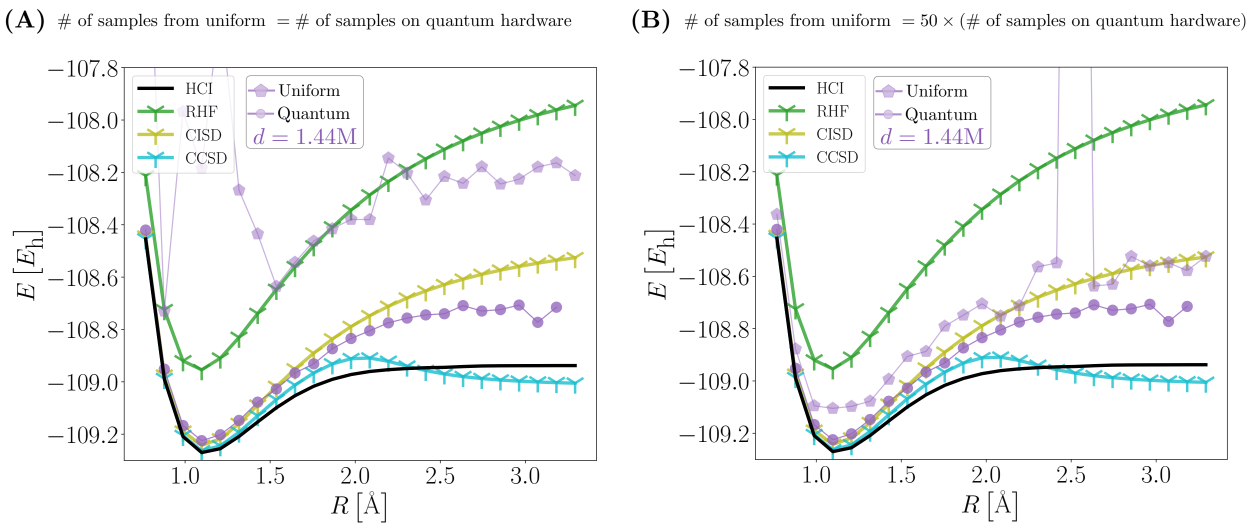

Figure S17: **Comparison of the estimator with configuration recovery applied to measurement outcomes from the quantum processor and configurations drawn from the uniform distribution, in the dissociation of N_2 (cc-pVDZ).** Pentagons correspond to the energies obtained with the configurations sampled from the uniform distribution while dots correspond to the energies obtained from configurations sampled from a quantum processor. Different classical methods are shown for reference, as indicated in the legend. **(A)** Uniform samples of configurations in the right particle sector. **(B)** Uniform samples of configurations over Fock space.

4.5.2 [2Fe-2S] experiments

Effect of configuration recovery on the accuracy of the HPC quantum estimator.– In this section, we compare the accuracy of the estimator without configuration recovery (i.e., applied to $\mathcal{X}_N \subset \tilde{\mathcal{X}}$) to the accuracy of the estimator with configuration recovery (applied to $\tilde{\mathcal{X}}$). As for all [2Fe-2S] experiments, $|\tilde{\mathcal{X}}| = 2.4576 \cdot 10^6$. In both cases, we take $K = 10$ batches of samples. When running the configuration recovery procedure, we use 10 self-consistent recovery iterations.

Fig. S18 shows the *Kernel Density Estimation* (KDE) of the $E^{(k)}$ distribution running SQD both with and without configuration recovery for different values of d. The qualitative behavior of the KDE for the estimator with and without vonfiguration recovery is similar. Upon increasing the value of d, the distributions shift towards lower values of the energy. However, the distributions of $E^{(k)}$ for the estimator with configuraiton recovery have lower energy than those without. Without configuration recovery, the best ground-state energy obtained is between the RHF and CISD energies, whereas when using the configuration recovery procedure, the energy is decreased even below the CCSD estimate. Again, note that the fact that the estimator without configuration recovery still has energies below RHF is a promising indication that the raw data from the processor contains a signal that the configuration recovery procedure can amplify.

Comparison of the quality of the estimator with configuration recovery, applied to quantum data and configurations drawn from the uniform distribution.– We first compare the performance of SQD with configuration recovery applied to measurement outcomes from the quantum processor versus configurations drawn from the uniform distribution over configurations in the right particle sector. In both cases we consider $K = 10$ batches of electronic configurations and the estimator is applied to $2.4576 \cdot 10^6$ sampled configurations. The self-consistent recovery procedure is warm-started from the orbital occupancy vector $\mathbf{n}$ obtained after running ten self-consitent iterations with $d = 250\text{K}$. Since the configuration recovery is warm-started, for $d > 250\text{K}$ we apply two self-consistent iterations. In both scenarios, the same values of d are considered. Recall that configuration recovery procedure recovers configurations that have the wrong particle number due to noise, thus the application of the estimator to configurations sampled from the uniform distribution in the right particle sector is equivalent to applying the SCI eigenstate solver to the random configurations. Fig. S19 shows a comparison of the energy-variance analysis obtained from

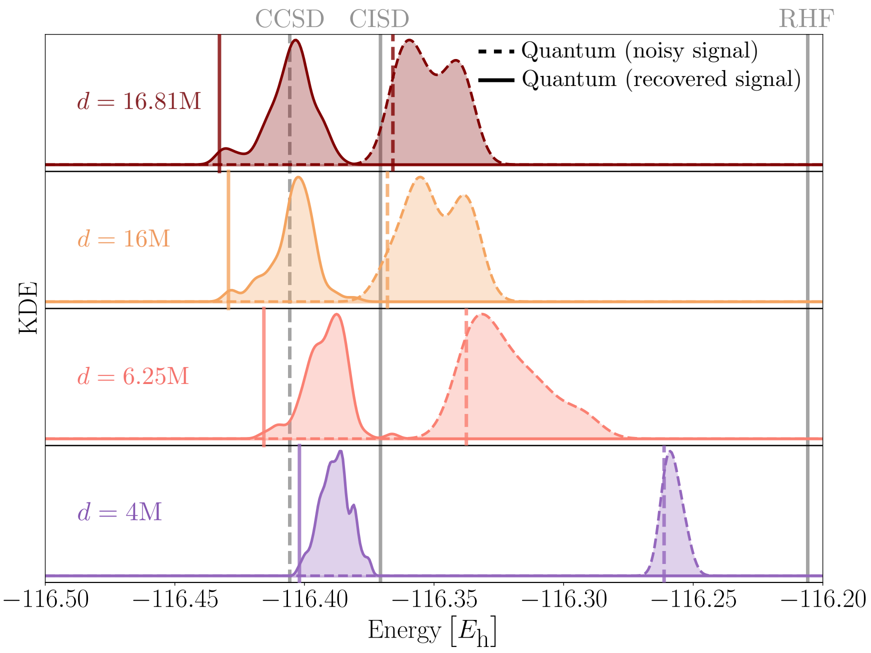

Figure S18: **Comparison of the performance of SQD, with and without configuration recovery, run on the same set of measurement outcomes for the ground state of [2Fe-2S].** The *kernel density estimation* (KDE) of the distribution of ground state energies obtained from the different batches of configurations is shown. The performance of the estimator with no configuration recovery (dashed lines) is compared to the performance of the estimator with configuration recovery (solid lines). Different panels correspond to different subspace dimensions d, as indicated in each panel. In each panel, the solid and dashed vertical lines matching the color of the KDE shows the lowest value of the energy amongst the different batches of configurations. The grey vertical lines show the value of the ground-state energy obtained from different classical methods, as indicated at the top of the panel.

the estimator on measurement outcomes from the quantum processor and on uniformly random configurations with the right particle number. When the configurations come from measurement outcomes from a quantum processor, the energy-variance analysis shows two clusters of points following a linear relation. The two clusters correspond to two eigenstates of the Hamiltonian. The extrapolated energy is in good agreement with the energy extrapolation from HCI calculations. The energy-variance analysis on the estimator run on the random configurations with the right particle number shows a different behavior. The energy-variance pairs are not clustered as clearly as in the previous scenario. For the largest values of d considered, we observe that a first cluster begins to form, which coincides with the eigenstate of the highest energy that both HCI and SQD applied to quantum measurement outcomes reveal. However, for these values of d the HPC estimator run on random samples is not capable of revealing the second eigenstate of lower energy that the estimator run on quantum data obtains.

We also compare the performance of the HPC estimator with configuration recovery run on the data obtained from the quantum processor versus on configurations drawn from the uniform distribution over the full Fock space. In both cases, we consider $K = 10$ batches of electronic configurations and the estimator is applied to $2.4576 \cdot 10^6$ sampled configurations . The self-consistent recovery procedure is warm-started from the orbital occupancy vector $\mathbf{n}$ obtained after running ten configuration recovery iterations with $d = 250$K. Since the configuration recovery procedure is warm-started, for $d > 250$K we apply two self-consistent operations. Fig. S20 (A) shows the comparison of the energy-variance analyses in both scenarios for values of the subspace dimension up to $d = 16$M. The qualitative behavior is similar, showing a cluster of points following a line that coincides with Eigenstate A of the Hamiltonian. However, $d \approx 16$M is enough for the estimator run on quantum samples to reveal the second cluster corresponding to Eigenstate B, while greater values of d are required in the case where the samples are obtained from the uniform distribution over the full Fock space. Being able to resolve eigenstates of lower energy with smaller values of d shows a more efficient description of the low-energy physics of the problem.

To quantify more precisely for which value of d the Eigenstate B cluster of energy-variance pairs appears, we study the evolution of $\Delta H/E^2$ as a function of d. A jump in $\Delta H/E^2$ reveals the discovery of a cluster in the energy-variance plane. Fig. S20 (B) shows the $\Delta H/E^2$ as a function of d in both scenarios. While a large enough value of d allows resolving Eigenstate B from the estimator

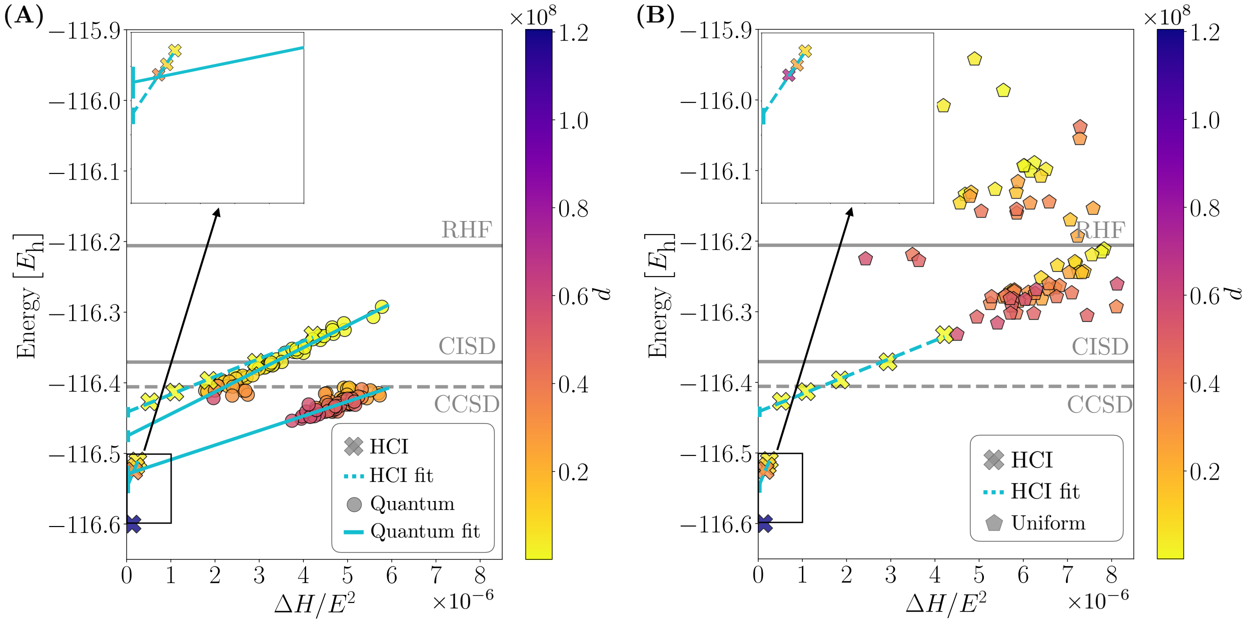

Figure S19: **Comparison of the energy-variance plane obtained from SQD run on samples from the quantum computer and samples from the uniform distribution.** Energy-variance analysis in [2Fe-2S] of the approximate eigenstates obtained by running SQD with configuration recovery on measurement outcomes from the quantum processor (panel **(A)**) and from the uniform distribution over the set of configurations that have the right particle number (panel **(B)**). Crosses show the energy-variance relation of HCI for reference, alongside the fit to a dashed line. Dots and pentagons correspond to energy-variance points obtained from the estimator applied to different batches of configurations. The number of determinants in each batch is encoded in the color bar. The inset is a zoom into the HCI extrapolation of the second Eigenstate.

with configuration recovery applied to the uniformly random samples, this transition requires the projection and diagonalization of the Hamiltonian in a subspace whose dimension is $d > 16$M. The observation of the same transition on the configurations sampled from the quantum processor requires at most $d = 12.25$M configurations. The difference in these dimensions is of at least 3.75M electronic configurations.

These results indicate that the quantum processor provides a useful signal that is used by SQD to procedure more accurate ground-state representations than using the same estimator on samples from uniform distribution.

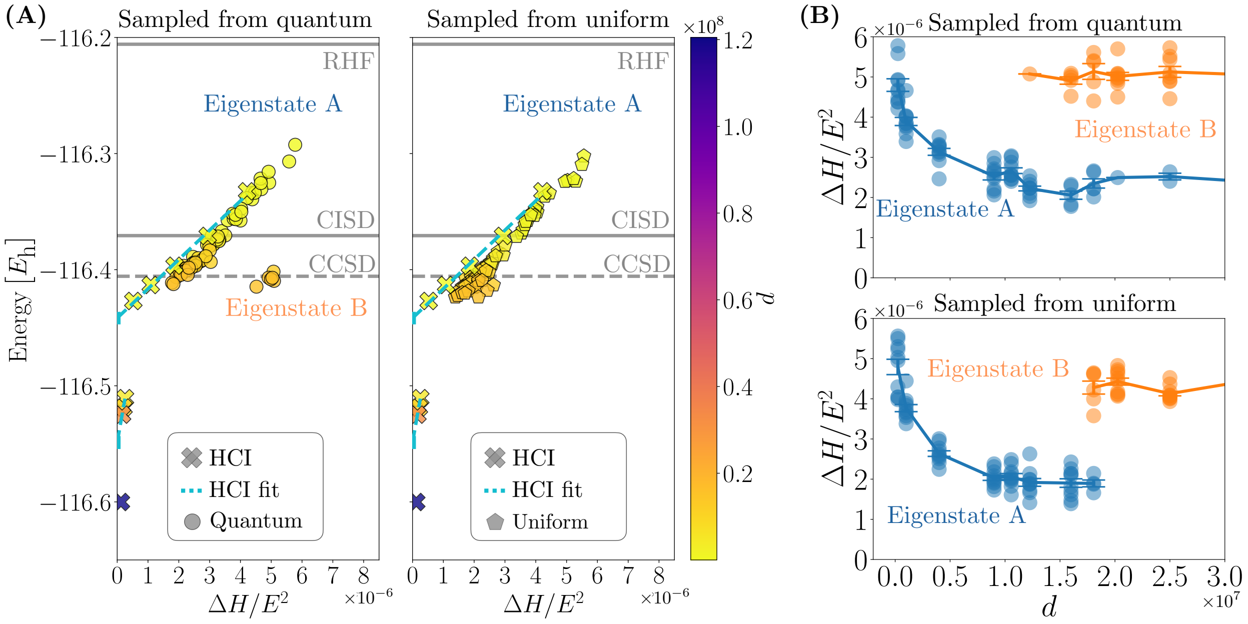

Figure S20: **Comparison of the energy-variance analysis of the low-energy spectrum of [2Fe-2S], obtained from the estimator with configuration recovery applied to quantum data and configurations drawn from the uniform distribution.** **(A)** The left panel shows energy-variance analysis of the low energy spectrum obtained by applying the estimator with configuration recovery to measurement outcomes from the quantum computer, while the right panel shows the same for samples from the uniform distribution on the Fock space. Crosses show the energy-variance relation of HCI for reference, alongside the fit to a (blue, dashed) line. Dots and pentagons correspond energy-variance points obtained from different batches of samples from the estimator. Subspace dimensions d are indicated by the colors of the points (corresponding to the colorbar), up to $d = 16$M, which is in the neighbourhood of the transition from Eigenstate A to B in the analysis applied to quantum samples. **(B)** Scatter plots of $\Delta H/E^2$ (horizontal axis on energy-variance plots) obtained from the estimator with configuration recovery on different batches of configurations, as a function of of the number of determinants d in each batch. The solid lines show the mean value of $\Delta H/E^2$ as a function of d, and the error bars correspond to the standard error of the mean. Different groups of data points are color coded according to which eigenstate they come from. The grey region indicates the interval of d where the transition from Eigenstate A to Eigenstate B occurs. Top panel corresponds to the application of the estimator with configuration recovery to configurations sampled from a quantum processor while the bottom panel corresponds to the application of the estimator with configuration recovery to configurations sampled from a uniform distribution over the Fock space.
